# First-in-class inhibitors of Nsp15 endoribonuclease of SARS-CoV-2: Modeling, synthesis, and enzymatic assay of thiazolidinedione and rhodanine analogs

**DOI:** 10.1016/j.jbc.2025.110409

**Published:** 2025-06-23

**Authors:** Nimer Mehyar, Nosaibah Samman, Shatha Al Gheribi, Abdullah Mashhour, Pearl Chan, Rabih O. Al-Kaysi, Stanley Perlman, Mohamed Boudjelal, Imadul Islam

**Affiliations:** 1 King Saud Bin Abdulaziz University for Health Sciences, Ministry of National Guard-Health Affairs, Riyadh, Saudi Arabia; 2 King Abdullah International Medical Research Center, Ministry of National Guards-Health Affairs, Riyadh, Saudi Arabia; 3 King Abdulaziz Medical City, Ministry of National Guard-Health Affairs, Riyadh, Saudi Arabia; 4 Department of Microbiology and Immunology, University of Iowa, Iowa City, Iowa, USA

**Keywords:** docking, endoribonuclease, enzyme mutation, FRET, inhibition mechanism, inhibitor, Nsp15, rhodanine, SARS-CoV-2, thiazolidinedione

## Abstract

During infection, the coronavirus nonstructural protein 15 (Nsp15), a uridine-specific endoribonuclease, suppresses the host cell’s antiviral response. Recently, researchers have paid more attention to this relatively underexplored yet potentially viable drug target. In this study, we employed FRET-based screening assays to identify potent Nsp15 inhibitors. Subsequently, we used active-site *in silico* docking methods to design new molecules with enhanced inhibitory properties. Solution assays were used to measure the potency and determine the mechanism of these inhibitors. We identified a novel class of thiazolidinedione and rhodanine analogs that inhibit severe acute respiratory syndrome coronavirus 2 (SARS-CoV-2) Nsp15. Docking these compounds into the uridine-binding site shows that most analogs form two hydrogen bonds with Ser294. The most potent inhibitors are compounds 5-(3-quinolin-4-yl-allylidene)-thiazolidine-2,4-dione (KCO237) and 5-(3-isoquinolin-4-yl-allylidene)-2-thioxo-thiazolidin-4-one (KCO251) (IC_50_: 0.304 μM and 0.931 μM, respectively). The inhibition kinetics of KCO237 and KCO251 best align with a reversible mixed inhibition model. Mutating Ser294 did not completely abolish Nsp15 activity or the inhibitory effect of KCO237 or KCO251. These findings suggest that thiazolidinedione and rhodanine analogs likely inhibit Nsp15 by binding to the uridine active site while also implicating a possible secondary allosteric-binding site. The ability of these compounds to inhibit VERO 6 cell infection with SARS-CoV-2 at subtoxic levels highlights their potential for development as novel antiviral treatments for SARS-CoV-2 and other coronavirus-related diseases.

Severe acute respiratory syndrome coronavirus 2 (SARS-CoV-2) is an enveloped, positive-sense RNA virus belonging to the Betacoronavirus genus of the Coronaviridae family within the order Nidovirales (1). In late 2019, SARS-CoV-2 triggered a global outbreak that resulted in the infection and death of nearly 18 million people (2). The symptoms of SARS-CoV-2 include coughing, shortness of breath, and fever; in severe cases, it can cause pneumonia, kidney failure, dyspnea, and death (3). The spike (S) protein of SARS-CoV-2 binds to the host cell receptor angiotensin-converting enzyme 2, which in turn activates the transmembrane serine protease TMPRSS2. These events facilitate viral fusion with host cell endosomes and entry into the host cell. Following entry, SARS-CoV-2 releases its genomic RNA into the host cell cytosol (4). The SARS-CoV-2 genome contains 14 ORFs that encode four structural proteins, 16 nonstructural proteins (nsps), and several accessory proteins (5). In the cytosol, host enzymes translate ORF1a and ORF1b into the viral polyproteins pp1a and pp1ab. These polyproteins are subsequently cleaved by viral proteases—3C-like protease (or nsp5) and papain-like protease (or nsp3)—to release multiple nsps (6, 7). These nsps assemble into replication–transcription complexes (RTCs). Nsp12, nsp13, nsp14, and nsp16 mediate the replication and transcription activities of the RTC (8, 9, 10). The RTCs facilitate the synthesis of new viral genomes and the translation of viral structural proteins. These viral components assemble with the genomic RNA to form new virions, which are secreted from the host cell *via* exocytosis, thereby spreading the infection to neighboring cells (9, 11, 12).

During the outbreak of the SARS-CoV-2 pandemic, drug discovery efforts were focused on developing vaccines to prevent the spread of the disease and alleviate symptoms (13, 14). Most of these vaccines were developed by raising neutralizing antibodies against the spike protein to block virus entry to the host cells (15). Other drug discovery efforts were oriented to small molecules targeting essential viral proteins like nirmatrelvir/ritonavir, which targets 3C-like protease, and molnupiravir and remdesivir, which target RdRp. Despite their effectiveness, the frequent mutations in the genes encoding the targeted proteins raise the risk of developing new drug-resistant SARS-CoV-2 variants (16). This emphasizes the need for discovering novel antiviral agents that act on alternative SARS-CoV-2 targets. Nsp15 stands out as a promising yet relatively less explored antiviral drug target (13).

Upon viral entry, RTCs transcribe the 3′-poly(A) tail of positive-sense RNA into a 5′-poly(U) region, which folds into stem–loop structures with dsRNA regions (17, 18, 19). The melanoma differentiation–associated protein 5 (MDA5) of the host cell identifies these dsRNA-like loops as pathogen-associated molecular patterns and forms a complex with them (17). The newly formed MDA5–dsRNA complex triggers a signaling cascade that induces type I interferon (IFN-1) and other antiviral genes (19). Nsp15 is a uridine-specific endoribonuclease (NendoU) that cleaves single-stranded RNA and dsRNA at uridine residues (17). Nsp15 cleaves the 5′-poly(U) on negative-sense RNA and reduces their length (17). Alternatively, it cleaves the 3′-poly(A) tail of positive-sense RNA and prevents their transcription to lengthy 5′-poly(U) (20). In both cases, Nsp15 prevents the formation of dsRNA-like loops and interferes with the MDA5–dsRNA complex assembly, thereby preventing the host cell’s IFN-1-mediated antiviral response (17, 20, 21, 22). Clearly, Nsp15 is not directly involved in viral replication/transcription; however, it is critical for infection as it enables the entering virus to evade the host cell’s IFN-1-mediated antiviral response (23). Evidently, mutations in the Nsp15 gene attenuated the virus's effects in both cell and animal models (24, 25). For that reason, Nsp15 represents a viable target; nonetheless, the value of targeting Nsp15 increases significantly when integrated into comprehensive therapeutic strategies that engage Nsp15 alongside multiple essential viral pathways, such as entry, protein processing, and replication (26).

The elucidation of the SARS-CoV-2 Nsp15 molecular structure enabled the discovery of several new inhibitors (23, 27, 28, 29, 30). One study has identified three natural compounds—epigallocatechin gallate, baicalin, and quercetin—as Nsp15 inhibitors. Among these, epigallocatechin gallate’s antiviral activity was confirmed through virtual docking, enzymatic activity, and plaque reduction neutralization tests (31). Another study reported that tipiracil, a uracil derivative, inhibits Nsp15 through a competitive mechanism; however, its antiviral activity was not evaluated. The crystal structure of Nsp15 bound to tipiracil revealed several key residues involved in binding, including His235, Gln245, Gly48, His250, Lys290, Val292, Ser294, Trp333, Thr341, Tyr343, and Lys345 (27). *In silico* screening of synthetic compound libraries has led to the identification of multiple potential Nsp15 inhibitors. One such study identified exebryl-1, a β-amyloid antiaggregation agent, as a potential inhibitor. Its inhibitory effect was confirmed *via in vitro* assays, and its antiviral activity was demonstrated in Vero 76, Caco-2, and Calu-3 cells (23). Another virtual screening effort identified CNP0111740 as a promising inhibitor, showing antiviral efficacy in Vero E6 cells at micromolar concentrations (28). A third study identified hexachlorophene, IPA-3, and CID5675221 as irreversible inhibitors predicted to covalently bind to cysteine residues in Nsp15. Their inhibitory effects were measured using an FRET-based assay, and antiviral activity was validated in Vero CCL-81 cells (29). A fourth virtual screening study identified NSC95397 as an Nsp15 inhibitor; however, it did not inhibit viral replication in Vero E6 cells (30). Finally, the aim of the present study is to employ FRET-based high-throughput screening and virtual docking to develop novel inhibitors of Nsp15. Despite their promising properties, none of these compounds have progressed to clinical trial phases.

As stated previously, the statement underscores the continuous need to discover new Nsp15 inhibitors that could successfully advance to clinical applications. To achieve this objective, we initially conducted an FRET-based high-throughput screen of in-house synthesized compounds. Then, we docked the most inhibitory compounds to the Nsp15 active site to reveal their interactions with the binding site. Afterward, we used these interactions as a guide to chemically modify the compounds with the purpose of enhancing their inhibitory effects. Subsequently, we validated our findings by mutation analysis. Finally, we characterized the properties of these compounds, including inhibition potency, reversibility, inhibition kinetic mechanism, viral infection inhibition, and cytotoxicity. We identified several novel SARS-CoV-2 Nsp15 inhibitors that hold potential for development into coronavirus disease 2019 treatments.

## Results

### SARS-CoV-2 Nsp15 purification and FRET-based assay screening

**Figure 1:**
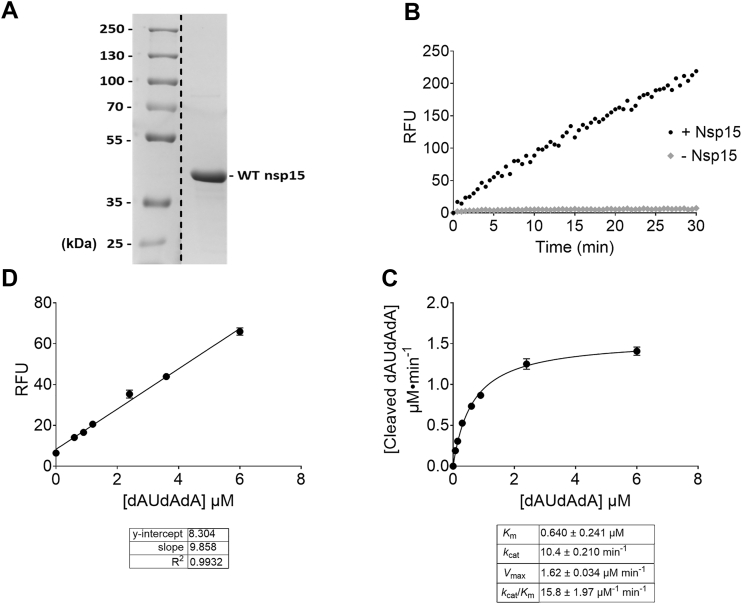
**Expression, activity, and inhibition of SARS-CoV-2 Nsp15**. *A*, gel showing purified wildtype SARS-CoV-2 Nsp15 protein, resolved by SDS-PAGE and detected by Coomassie staining. *Lane 1*, prestained molecular standards (Bio-Rad). *Lane 9*, eluted SARS-CoV-2 Nsp15 from Ni–NTA column (lanes 2–8 of purification steps were removed for clarity; a *dashed line* indicates the splice). See Figure S1 for full gel image. *B*, plot showing the change in fluorescence (excitation 495 nm and emission 520 nm) because of the cleavage of the 5′-6-FAM-dArUdAdA-6-TAMRA-3′ substrate. Reactions were allowed to proceed for 30 min at 25 °C with 1.2 μM substrate, either without (♦) or with 150 nM SARS-CoV-2 Nsp15 (•). *C*, standard curve (linear fit) showing the relationship between 5′-6-FAM-dAUdAdA with no TAMRA quencher and fluorescence. *D*, plot showing the relationship between wildtype SARS-CoV-2 Nsp15 activity rate and the concentration of the substrate 5′-6-FAM-dArUdAdA-6-TAMRA-3’. The enzymatic activity was measured by monitoring fluorescence changes (excitation 495 nm and emission 520 nm) over a 30-min incubation at 25 °C. Reactions were performed with 150 nM enzyme and increasing concentrations of the fluorogenic substrate 5′-6-FAM-dArUdAdA-6-TAMRA-3’. *K*_*m*_, *V*_max_, and *k*_cat_ values (mean ± SD) were calculated by fitting the curve into the Michaelis–Menten equation using GraphPad Prism (v = *V*_max_[S]/(KM + [S])), with the enzyme concentration (Et) fixed at 0.15 μM. The catalytic efficiency (*k*_cat_/*K*_*m*_) value (mean ± SD) was subsequently calculated. *Error bars* represent the standard deviation of duplicate samples. FAM, carboxyfluorescein; Nsp, nonstructural protein; SARS-CoV-2, severe acute respiratory syndrome coronavirus 2; TAMRA, carboxytetramethylrhodamine.

Following expression in *Escherichia coli* BL21 (DE3) cells and purification using a Ni–NTA–agarose column, 1 l of culture media yielded approximately 1 mg of SARS-CoV-2 Nsp15. The purity of the enzyme was confirmed by SDS-PAGE (Fig. 1*A* and Fig. S1). To assess Nsp15 endoribonuclease activity, we monitored fluorescence emission at 520 nm (excitation at 495 nm) resulting from the hydrolysis of the FRET-labeled substrate 5′-6-FAM-dArUdAdA-6-TAMRA-3′ (Fig. 1*B*). To convert the relative fluorescence unit into the concentration of cleaved dAUdAdA per unit time, we generated a standard curve using a carboxyfluorescein (FAM)-labeled tetranucleotide lacking the carboxytetramethylrhodamine (TAMRA) quencher. The resulting linear equation was used for relative fluorescence unit-to-concentration conversion (Fig. 1*C*). Control experiments were conducted in parallel to rule out potential signal interference from assay components (Fig. S2*A*). Kinetic parameters of SARS-CoV-2 Nsp15 were determined by measuring FRET signal changes across a range of substrate concentrations. Then, we fitted the results by the Michaelis–Menten equation to obtain the *K*_*m*_, *V*_max_, and *k*_cat_ using GraphPad Prism (GraphPad Software, Inc) and calculated the specificity constant (Fig. 1*D*).

**Figure 2:**
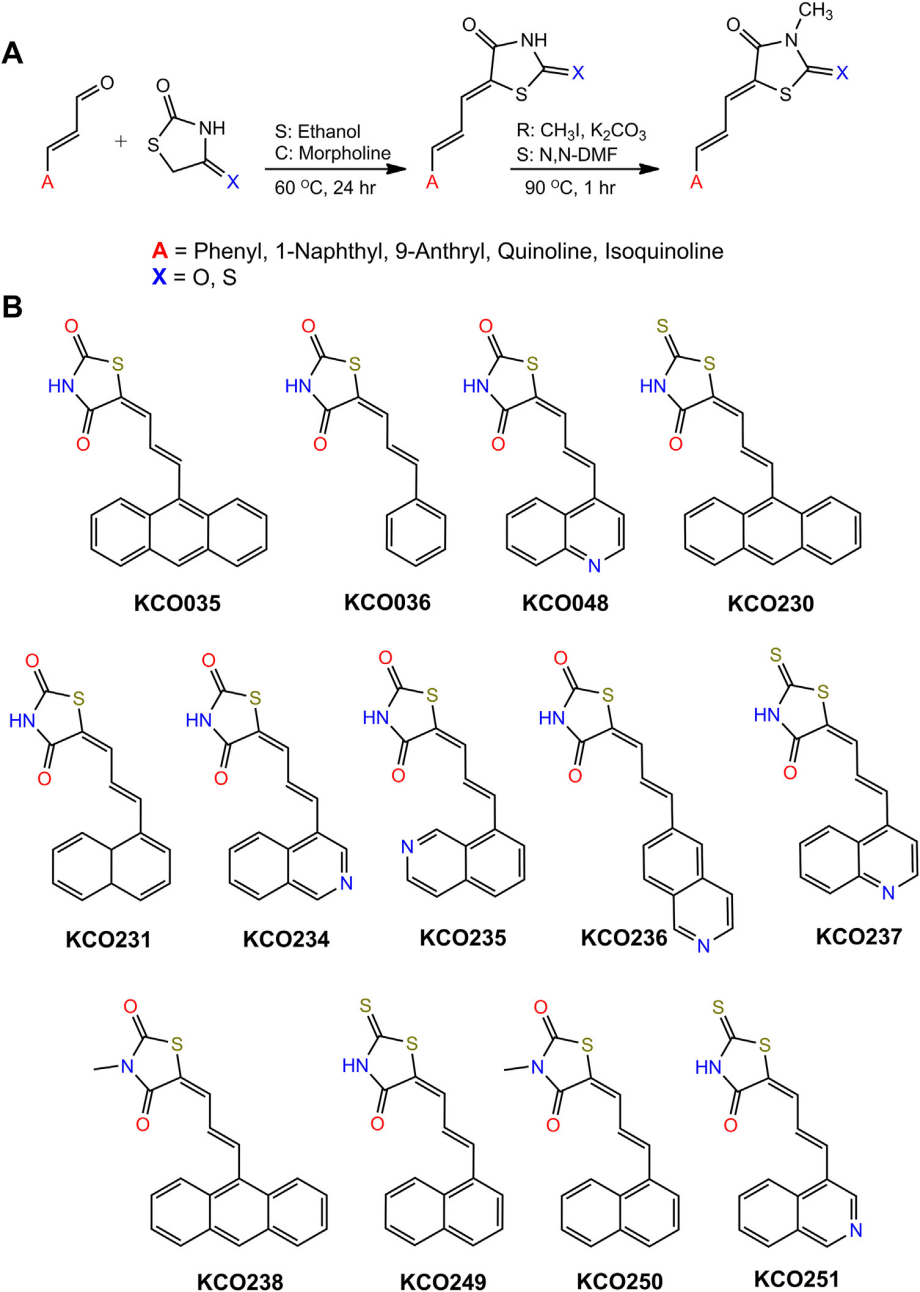
**Chemical synthesis and structure of new anthracene and rhodanine (RH) analogs.***A*, general scheme of thiazolidinedione (TZD) and RH analog synthesis. *B*, molecular structures of anthracene and RH analogs.

In our laboratory’s pursuit of potential antiviral agents against SARS-CoV-2, we adapted a 96-well FRET-based assay platform to screen a library of in-house synthesized compounds representing diverse chemical classes, each tested at a concentration of 100 μM. While several compounds exhibited inhibitory effects on Nsp15 activity at this concentration, none exceeded 25% inhibition—except for one standout molecule, KCO035. At 100 μM, KCO035 inhibited nearly 80% of Nsp15 activity (Fig. S3). KCO035 belongs to a class of hybrid molecules characterized by an anthracene base and a thiazolidinedione head group. The potent inhibitory effect observed suggests that the anthracene moiety, the thiazolidinedione moiety, or a combination of both structural components contributes to the compound’s ability to inhibit Nsp15. We synthesized several analogs of KCO035 and characterized them by NMR spectroscopy (Fig. 2, *A* and *B*; Fig. S4).

### Docking of inhibitors in the SARS-CoV-2 Nsp15 active site

**Figure 3:**
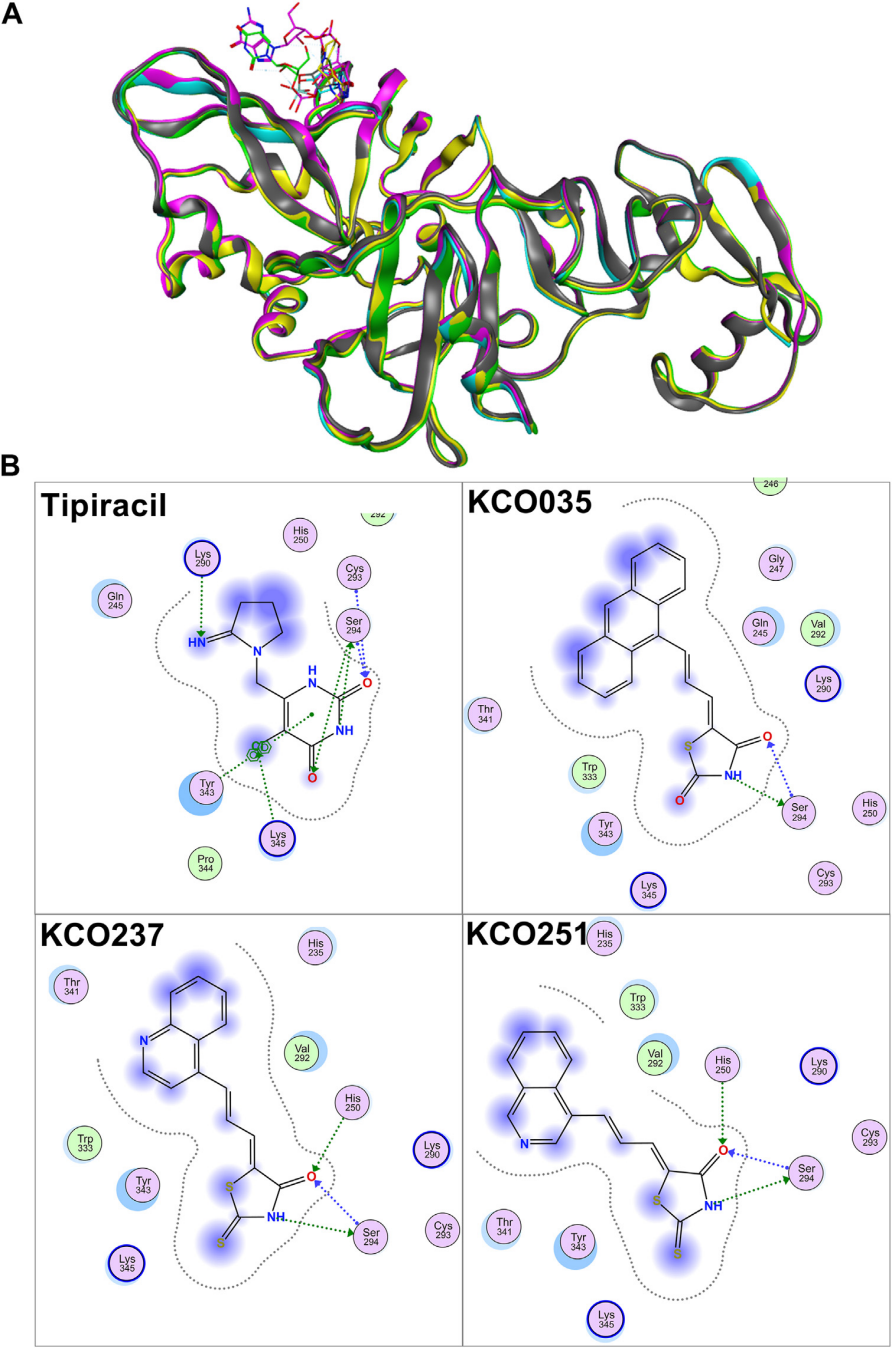
**Docking of inhibitors in the SARS-CoV-2 Nsp15 active site**. *A*, model showing the superimposition of the atomic structures of a monomer of SARS-CoV-2 Nsp15; PDB_ID: 6WLC (*cyan*), PDB_ID: 6WXC (yellow), PDB_ID: 6X1B (*magenta*), PDB_ID: 6X4I (*green*), and PDB_ID: 7K1L (*gray*) and the atomic structure of SARS-CoV-2 Nsp15 (*magenta*; PDB_ID: 5WWP). *B*, diagram showing the interaction maps of tipiracil and rhodanine analogs docked to a rigid SARS-CoV-2 Nsp15-binding site using MOE. The acidic, basic, and polar amino acids are represented by the *pink spheres* with *blue*, *red*, and *black outlines*, respectively. Hydrophobic residues are presented in *green*. *Blue and green arrows* indicate hydrogen bonding to backbone and side-chain atoms, respectively. The *fuzzy blue spheres* represent the ligand atoms exposed to solvent as determined by the docking process. *Light-blue shadows* around residues indicate the degree of interaction with ligand atoms. The *dotted contour* reflects steric. The steric room for methyl substitution is presented by *dotted contours*. MOE, molecular operating environment; Nsp, nonstructural protein; PDB, Protein Data Bank; SARS-CoV-2, severe acute respiratory syndrome coronavirus 2.

A previous study demonstrated that tipiracil, a Food and Drug Administration–approved drug for colorectal cancer, binds to the uridine recognition site of SARS-CoV-2 Nsp15 and acts as a competitive inhibitor (27). To investigate whether KCO035 and related analogs interact within the same binding pocket, we first identified the active site of Nsp15 by superimposing multiple crystal structures: Protein Data Bank (PDB) IDs 6WLC, 6WXC, 6X1B, 6X4I, and 7K1L. Structural alignment revealed moderate variation in the enzyme’s overall conformation (RMSD = 1.88 Å); nevertheless, residues comprising the binding pocket remained largely conserved across all structures (RMSD = 0.33 Å) (Fig. 3*A*). Using the Nsp15 crystal structure cocrystallized with tipiracil (PDB_ID: 6WXC), we carried out molecular docking studies in the molecular operating environment (MOE). As an initial validation step, we redocked tipiracil into the Nsp15 active site (Fig. 3*B*). Tipiracil forms three direct hydrogen bonds with Ser294 *via* its pyrimidine atoms O2, O4, and N3. An additional hydrogen bond was observed between the O_2_ atom and Cys293. The pyrimidine ring also established hydrogen bonds with Tyr343 and Lys345, whereas a strong polar interaction was predicted with Gln245. Although the model recapitulated many interactions, it did not fully capture all contacts seen in the experimental structure (27).

Next, we docked KCO035 and other thiazolidinedione and rhodanine analogs in the tipiracil-binding site (Figs. 3*B* and S5). Ten of the docked analogs formed two hydrogen bonds with Ser294 *via* their N3 and O4 atoms, closely resembling the interaction pattern observed between tipiracil’s pyrimidine ring and Ser294. Among these, analogs 3-methyl-5-(3-anthracen-9-yl-allylidene)-thiazolidine-2,4-dione (KCO238) and 3-methyl-5-(3-naphthalen-1-yl-allylidene)-thiazolidine-2,4-dione (KCO250) retained the O4–Ser294 hydrogen bond; however, the N3 interaction was lost because of the presence of a tertiary amine, which likely disrupted hydrogen bond formation. Notably, 5-(3-isoquinolin-6-yl-allylidene)-thiazolidine-2,4-dione (KCO236) was the only analog that formed a hydrogen bond with Ser294 through its S1 atom. The flipped position of the thiazolidinedione ring of KCO236 enabled this bonding. Finally, the anthracene base of KCO035 appears exposed to the solvent. This is similar to iminopyrroldine ring exposure in the tipiracil docking experiment. However, it is important to highlight that in the crystal structure, the iminopyrrolidine ring is not solvent exposed. Instead, it is shielded by a phosphate group located within a cavity formed by His235, His250, and Thr341. Compounds containing smaller aromatic bases, such as naphthalene, quinoline, or isoquinoline, also exhibited similar solvent exposure patterns, consistent with the docking pose of KCO035 and the modeled behavior of tipiracil. Overall, the similarity of tipiracil interactions in the uridine site of Nsp15 with the interactions of compounds that comprise an anthracene base connected to a thiazolidinedione head suggests that these compounds could inhibit Nsp15 competitively. In addition, these docking experiments reaffirmed the critical role of Ser294 in the uridine-binding site of SARS-CoV-2 Nsp15.

For each compound, we quantified the number of hydrogen bonds, hydrophobic interactions, and ionic interactions with key active-site residues. The total number of these interactions was then calculated to assess binding strength and complementarity. We conducted two docking protocols: rigid and flexible. The docked molecules were categorized into four classes based on the number of interactions and the overall shape similarity of their poses to the X-ray structure of tipiracil. Using this classification, we identified compounds 5-(3-isoquinolin-8-yl-allylidene)-thiazolidine-2,4-dione (KCO235) and 5-(3-quinolin-4-yl-allylidene)-2-thioxo-thiazolidine-4-one (KCO237) as category 1. These compounds exhibited the highest number of interactions with Ser294 and showed the best shape overlap with the Nsp15–tipiracil X-ray structure. In contrast, compounds 5-(3-anthracen-9-yl-allylidene)-thiazolidine-2,4-dione (KCO35), KCO238, and KCO250 were placed in category 4 because of their minimal interactions with Ser294 and the lowest structural similarity. The remaining compounds were classified under categories 2 and 3.

We applied similar criteria for classifying molecules in the flexible docking protocol. Notably, some compounds displayed altered interaction patterns under flexible docking conditions (Table 1). The analog KCO036 failed to dock within the active site and was therefore excluded from categorization. In addition, docking experiments were not performed for compounds KCO058 and KCO233 as they belonged to different chemical families. Compared with rigid docking, flexible docking did not alter most ligand interactions with Ser294 or the shape of the binding site. This consistency strengthens confidence in the reliability of the predicted interactions. Although flexible docking did not increase interactions with Ser294 for 5-(3-naphthalen-1-yl-allylidene)-thiazolidine-2,4-dione (KCO231), 5-(3-naphthalen-1-yl-allylidene)-2-thioxo-thiazolidin-4-one (KCO249), KCO236, and KCO035, their category assignment improved. This enhancement was attributed to greater shape similarity of their docked poses to the X-ray structure under flexible docking conditions.

**Table 1:** Docking categories and IC_50_ values of tipiracil and rhodanine analogs

Compound	Binding category^a^		IC_50_, μM^b^
	Rigid docking	Flexible docking	
Tipiracil	1	1	>1.00 × 10^3^
KCO235	1	1	4.17 (0.716)
KCO237	1	1	0.304 (0.102)
KCO231	2	1^c^	4.41 (0.312)
KCO251	2	2	0.931 (0.555)
KCO048	2	2	1.35 (0.511)
KCO234	2	2	7.38 (2.11)
KCO249	3	1^c^	1.35 (0.511)
KCO236	3	2^c^	0.932 (0.0210)
KCO230	3	3	4.58 (1.88)
KCO035	4	3^c^	7.21 (1.54)
KCO238	4	4	106 (12.3)
KCO250	4	4	>1.00 × 10^3^
KCO036	-	-	9.40 (2.17)

a MOE was used to dock compounds into the active site of Nsp15 (PDB_ID: 6WXC). Prior to docking, water molecules and anions were removed. Both the enzyme and ligands were protonated at pH 8.0. Rigid docking was performed with the protein held fixed and ligands allowed to be flexible. In contrast, flexible docking allowed both enzyme and ligand flexibility. Categories are ranked from 1 to 4, with 1 having the most interactions with Ser294 and the best structural overlap.

b The enzymatic activity of wildtype SARS-CoV-2 Nsp15 was measured by monitoring fluorescence changes (excitation at 495 nm and emission at 520 nm) over a 30-min incubation at 25 °C. Reactions were performed with 150 nM enzyme, 1.2 μM of the fluorogenic substrate (5′-6-FAM-dArUdAdA-6-TAMRA-3′), and increasing concentrations of rhodanine inhibitors. IC_50_ values (mean ± SD) were determined by fitting dose–response curves to a four-parameter logistic model (log[inhibitor] *versus* response) using GraphPad Prism: (Y = bottom + (top–bottom)/(1 + 10 ((LogIC50-X) ∗ HillSlope))). Standard deviations are for duplicate samples.

c Docking to a flexible binding site improved ligand binding.

### SARS-CoV-2 Nsp15 inhibition

**Figure 4:**
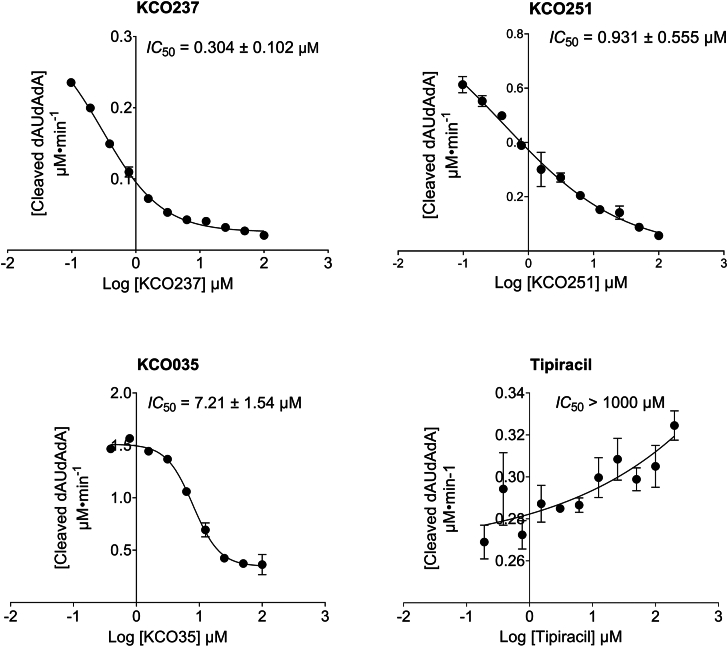
***IC*_50_ determination using the FRET-based Nsp15 activity assay**. Plots showing the change in fluorescence (excitation 495 nm and emission 520 nm) because of the cleavage of 5′-6-FAM-dArUdAdA-6-TAMRA-3′ substrate by wildtype SARS-CoV-2 Nsp15. Reactions were allowed to proceed for 30 min at 25 °C in the presence of 150 Nm of SARS-CoV-2 Nsp15, 1.2 mM substrate, and increasing concentrations of various rhodanine analogs. IC_50_ values (mean ± SD) were calculated by fitting dose–response curves to log[inhibitor] *versus* response–variable slope curves (four parameters) in GraphPad Prism Y = bottom + (top–bottom)/(1 + 10 ((LogIC50-X)∗HillSlope)). Error bars represent the standard deviation of duplicate samples. FAM, carboxyfluorescein; TAMRA, carboxytetramethylrhodamine; Nsp15, nonstructural protein 5; SARS-CoV-2, severe acute respiratory syndrome coronavirus 2.

To evaluate the inhibitory effects of the study compounds on SARS-CoV-2 Nsp15 activity, we employed an FRET-based assay. These assays were performed in 96-well plates using varying concentrations of the test compounds (Fig. 4 and Fig. S6). The resulting inhibition data were then fitted to a dose–response curve to determine the IC_50_ values (Table 1). Control experiments were conducted in parallel to rule out potential signal interference from the tested compounds (Fig. S2, *B* and *C*). Contrary to a previously published study, tipiracil did not inhibit Nsp15 activity (27). Subsequently, we assessed the inhibitory effect of compound KCO035 on Nsp15. KCO035, which contains an anthracene base group and a thiazolidinedione head group, showed a mild inhibitory effect. Modifying the head group to rhodanine while retaining the anthracene base, as in 5-(3-anthracen-9-yl-allylidene)-2-thioxo-thiazolidin-4-one (KCO230), nearly doubled the compound’s inhibitory activity. Conversely, replacing the anthracene base with a naphthalene analog while maintaining the thiazolidinedione head group, as in KCO231, also doubled the inhibitory effect. However, further structural simplification by removing an additional ring, as seen in KCO036, led to a reduced inhibitory effect (IC_50_ = 9.4 μM). The use of naphthalene as a base and rhodanine as a head group in KCO249 further enhanced its inhibitory effect by sixfold. Encouraged by the increased potency observed when transitioning from anthracene to naphthalene, we introduced a nitrogen atom into the naphthalene ring to form the quinoline base. The compound KCO048, which comprised a quinoline base and thiazolidinedione head group, exhibited a threefold greater inhibitory effect than KCO231. Replacing the thiazolidinedione head with rhodanine while retaining the quinoline base, as in KCO237, resulted in a fivefold increase in potency compared with KCO048. In contrast, substituting the quinoline base with isoquinoline in 5-(3-isoquinolin-4-yl-allylidene)-thiazolidine-2,4-dione (KCO234) did not enhance inhibitory activity. However, replacing thiazolidinedione with rhodanine in 5-(3-isoquinolin-4-yl-allylidene)-2-thioxo-thiazolidin-4-one (KCO251) led to an eightfold increase in inhibition potency. Modifying the position of the nitrogen atom within the isoquinoline base, as in KCO235 and KCO236, produced a moderate increase in inhibitory effect. These findings suggest that the “dance of nitrogen” in the lower part of the molecule substantially influences Nsp15 inhibition. Docking experiments indicated a strong hydrogen bond between the –NH group of the thiazolidinedione or rhodanine moiety and the residue Ser294, prompting further investigation of this interaction. Accordingly, we synthesized N-methylated analogs, KCO238 and KCO250. As predicted, these compounds did not inhibit Nsp15 activity.

Contrary to a previously published study, tipiracil did not inhibit Nsp15 activity in our assay conditions (27). We validated these findings by replicating the experiment using the assay conditions from the mentioned study, substituting our FAM/TAMRA-labeled RNA for the 3′-32P-labeled RNA octamer substrate and using 10 nM Nsp15. Tipiracil did inhibit Nsp15 under these conditions but to a lesser extent than KCO237 and KCO251 (Fig. S7).

**Figure 5:**
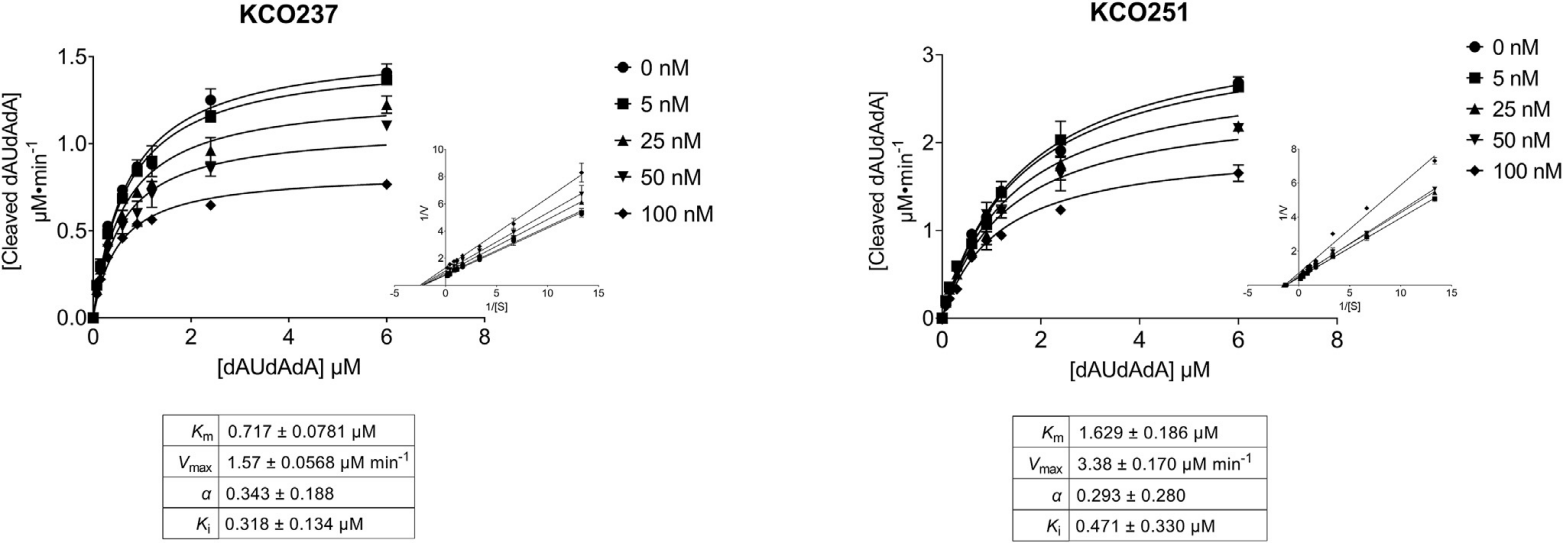
**Mechanism of SARS-CoV-2 Nsp15 inhibition**. *A* and *B*, plots showing the change in fluorescence (excitation 495 nm and emission 520 nm) because of the cleavage of 5′-6-FAM-dArUdAdA-6-TAMRA-3′ substrate by wildtype SARS-CoV-2 Nsp15. Reactions were allowed to proceed for 30 min at 25 °C in the presence of 150 nM of SARS-CoV-2 Nsp15, 1.2 mM substrate, and increasing concentrations of KCO237 or KCO251. The values of kinetic parameters *K*_*m*_, *V*_max_, *α*, and *K*_i_ (mean ± SD) were determined by fitting data points to a mixed inhibition model using GraphPad Prism, based on the following equations: *V*_maxapp_ = *V*_max_/(1 + [I]/(α × Ki)), *K*_mapp_ = *K*_*m*_ × (1 + [I]/Ki)/(1 + [I]/(α × Ki)), and Y = *V*_maxapp_ × [S]/(Km_app_ + [S]). The fitness of the mixed inhibition model was confirmed using the extra sum-of-squares *F* test in GraphPad Prism with statistical significance (*p <* 0.05) (Tables S1 and S2). *Subsets*: plots represent the Lineweaver–Burk transformation of inhibition data presented in the main panel. *Error bars* represent the standard deviation of duplicate samples. FAM, carboxyfluorescein; Nsp15, nonstructural protein 15; SARS-CoV-2, severe acute respiratory syndrome coronavirus 2; TAMRA, carboxytetramethylrhodamine.

Researchers of the previous study demonstrated that tipiracil binds to the uridine active site of SARS-CoV-2 Nsp15, leading them to reasonably predict that tipiracil acts as a competitive inhibitor. In the current study, docking experiments clearly showed that thiazolidinedione and rhodanine analogs interact with the tipiracil-binding site in Nsp15. Therefore, we anticipated that the compounds KCO237 and KCO251 would also inhibit Nsp15 competitively. However, to our surprise, when we fitted the inhibition curves of these compounds to various inhibition models, they aligned best with the mixed inhibition model (Fig. 5; Table S1 and Table S2). This finding is consistent with a previous study on a rhodanine scaffold–based molecule, which predicted the involvement of an allosteric binding site—potentially explaining the mixed inhibition behavior (29).

**Figure 6:**
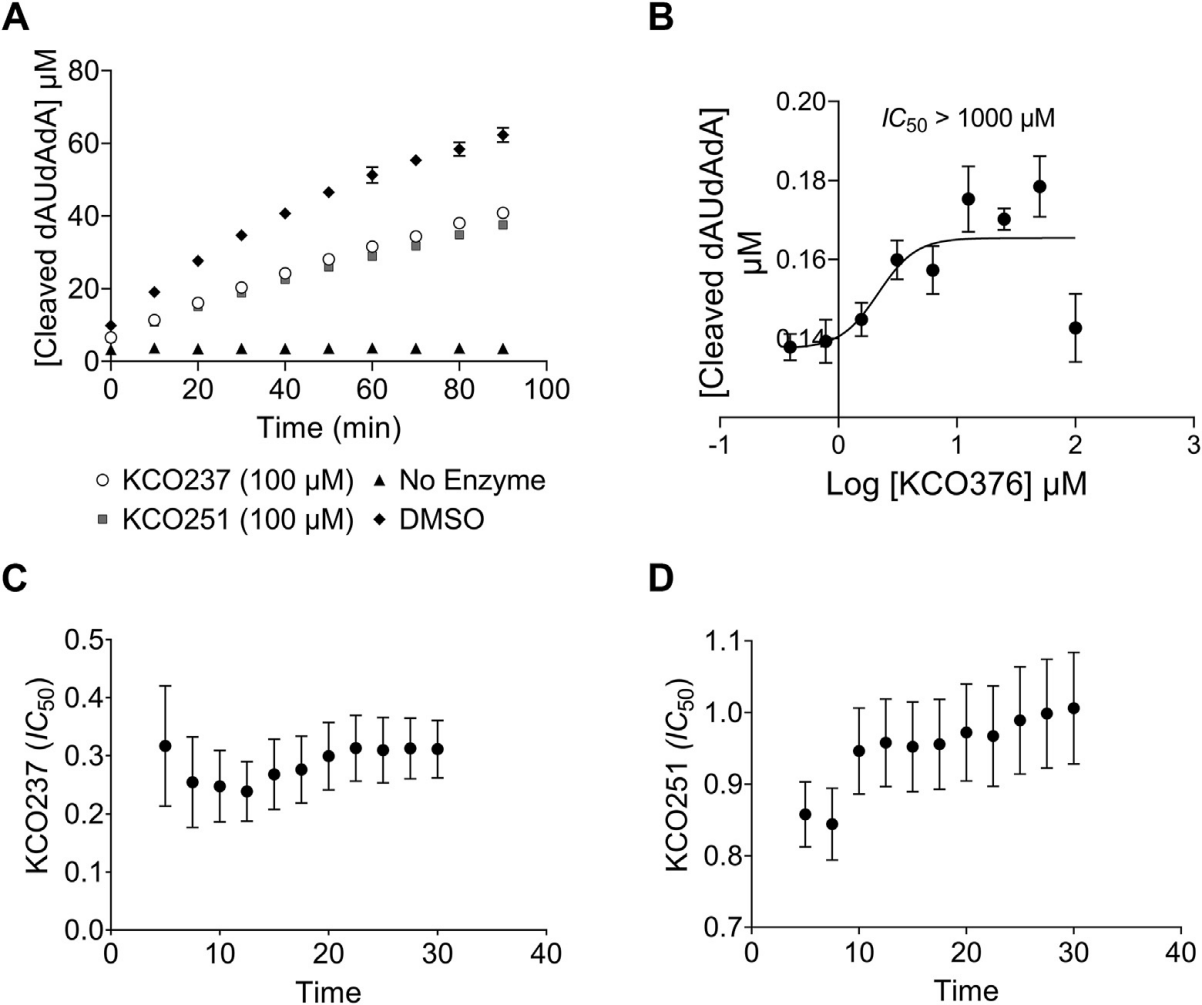
**Analysis of inhibitor reversibility**. *A*, plot showing Nsp15 activity over time after inhibitor dilution. Nsp15 (1 μg) was preincubated with the indicated concentrations of inhibitors in a cold room for 60 min and subsequently diluted 1:100 into reaction buffer containing 1 μM substrate. Fluorescence measurement was taken (*t* = 0) and recorded every 5 min for a total of 90 min. *B*, plot showing the change in fluorescence (excitation 495 nm and emission 520 nm) because of the cleavage of 5′-6-FAM-dArUdAdA-6-TAMRA-3′ substrate by wildtype SARS-CoV-2 Nsp15. Reactions were allowed to proceed for 30 min at 25 °C in the presence of 150 nM of SARS-CoV-2 Nsp15, 1.2 μM substrate, and increasing concentrations of saturated KCO237 analog (KCO376). IC_50_ value (mean ± SD) was calculated by fitting dose–response curves to log[inhibitor] *versus* response–variable slope curves (four parameters) in GraphPad Prism Y = bottom + (top–bottom)/1 + 10 ((LogIC50-X)∗HillSlope)). *C* and *D*, plots showing IC_50_ values of KCO237 and KCO251 *versus* time. Reactions were allowed to proceed for 30 min at 25 °C in the presence of 150 nM of SARS-CoV-2 Nsp15, 1.2 μM substrate, and increasing concentrations of various rhodanine analogs. IC_50_ values were calculated every 5 min for a total of 30 min by fitting dose–response curves to log[inhibitor] *versus* response–variable slope curves (four parameters) in GraphPad Prism Y = bottom + (top–bottom)/(1 + 10 ((LogIC50-X)∗HillSlope)). *Error bars* represent the standard deviation of duplicate samples. FAM, carboxyfluorescein; Nsp15, nonstructural protein 15; SARS-CoV-2, severe acute respiratory syndrome coronavirus 2; TAMRA, carboxytetramethylrhodamine.

Finally, it is important to note that these inhibitors contain an electron-deficient diene system conjugated to a carbonyl group within the thiazolidinone ring. This system can act as a Michael acceptor, particularly with nearby residues Cys293 or Lys345 (32). The formation of a covalent bond between these residues and the inhibitor implies that the inhibition is irreversible. We examined the reversibility of the enzymatic reaction using the dilution method. We monitored Nsp15 activity after a high-fold dilution of the inhibitor (Fig. 6*A*). Nsp15 recovered its activity when we significantly diluted KCO237 and KCO251. In addition, we saturated the olefins in KCO237 and verified it by mass spectrometer. We tested the saturated KCO237 effect on Nsp15 activity (Fig. 6*B*). The saturated-olefin analog of KCO237 did not exhibit any inhibitory effect. This outcome confirms the importance of the olefin moiety for inhibitor interaction with the binding site. The rigid nature of the double bond could be essential for holding the ligand in the proper orientation. However, we cannot exclude the possibility of forming a reversible covalent bond with Cys293 or Lys345. Studies indicate that the inhibition of reversible covalent inhibitors changes with time (33). This phenomenon is usually characterized by a decreasing IC_50_ value with time. We investigated the effect of time on the IC_50_s of KCO237 and KCO251 and found that their IC_50_s were consistent through the period of the assay (Fig. 6*C*; Fig. S8). This result supports the conclusion that KCO237 is a reversible, noncovalent inhibitor.

### Mutation analysis of SARS-CoV-2 Nsp15

**Figure 7:**
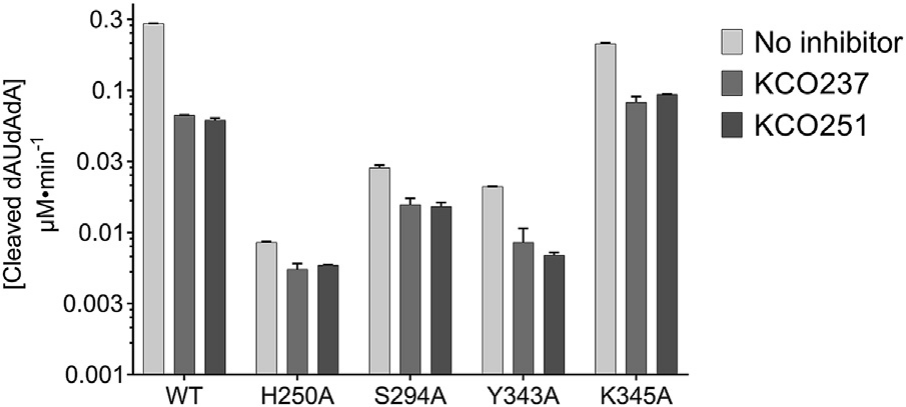
**Mutant Nsp15 inhibition**. Bar graph and plots showing the change in fluorescence (excitation 495 nm and emission 520 nm) because of the cleavage of 5′-6-FAM-dArUdAdA-6-TAMRA-3′ substrate by wildtype, H250A, S294A, Y343A, or K345A SARS-CoV-2 Nsp15. Reactions were allowed to proceed for 30 min at 25 °C in the presence of 150 Nm of mutant or wildtype SARS-CoV-2 Nsp15, 1.2 mM substrate, and 100 mM of KCO237 or KCO251. *Error bars* represent the standard deviation of duplicate samples. FAM, carboxyfluorescein; KCO237, 5-(3-quinolin-4-yl-allylidene)-thiazolidine-2,4-dione; KCO251, 5-(3-isoquinolin-4-yl-allylidene)-2-thioxo-thiazolidin-4-one; Nsp15, nonstructural protein 15; SARS-CoV-2, severe acute respiratory syndrome coronavirus 2; TAMRA, carboxytetramethylrhodamine.

Docking experiments revealed direct interactions between the residues His250 and Ser294 and most of the thiazolidinedione and rhodanine analogs, whereas fewer analogs interacted with Tyr343 and Lys345. To further investigate these interactions, we performed mutation analysis on these residues. We expressed and purified the mutants Tyr343Ala, His250Ala, Lys345Ala, and Ser294Ala using the same procedures previously applied for the wildtype protein (Fig. S1). We then determined the kinetic parameters *K*_*m*_ and *k*_cat_ of these mutants by fitting their activity curves to the Michaelis–Menten equation (Table 2; Fig. S9). In addition, we analyzed the inhibition data of these mutants using dose–response curves to obtain the IC_50_ for each inhibitor (Table 2). Mutants His250Ala, Ser294Ala, and Tyr343Ala exhibited a significant loss of activity, as reflected by markedly reduced *k*_cat_ values. Mutations affected the ability of Nsp15 to bind to the substrate. The moderate increase of mutants’ *K*_*m*_ confirms this conclusion. These results highlight the essential roles of His250, Ser294, and Tyr343 in both substrate binding and the catalytic mechanism. Despite this significant loss of these mutants’ activity, some residual activity remains detectable. This residual activity suggests that these mutants are capable of adopting an alternative, albeit less efficient, catalytic pathway that bypasses the involvement of the mutated residues. Compared with the wildtype, the catalytic efficiency of the His250Ala, Ser294Ala, and Tyr343Ala mutants decreased significantly—by 150-, 85-, and 40-fold, respectively. This residual activity also implies that while these residues are crucial for substrate binding, their mutation did not completely abolish it. In contrast, the catalytic efficiency of the Lys345Ala mutant decreased by only threefold, suggesting a more limited role for Lys345 in both substrate binding and the catalytic mechanism. The residual activities of these mutants allowed us to examine the inhibitory effect of KCO237 (Table 2). However, we were unable to fit the inhibition data of the mutants to dose–response curves and, therefore, could not accurately calculate the IC_50_ values (Figs. S10; S11). Nevertheless, all four mutants—His250Ala, Ser294Ala, Tyr343Ala, and Lys345Ala—retained approximately 50% of their residual activity at 100 μM KCO237. This observation indicates that mutating these residues did not entirely eliminate the inhibitory effect of KCO237 (Fig. 7).

**Table 2:** Kinetic parameters of activity and inhibition of mutant SARS-CoV-2 mutants

Mutant	*K*_*m*_, μM^a^	*k*_cat_, min^−1^^a^	*k*_cat_/*K*_*m*_, μM^−1^ min^−1^^a^	*IC*_50_, μM (KCO237 or KCO251)^b^
His250Ala	5.55 (1.27)	0.554 (0.771)	0.0998 (0.141)	>1.00 × 10^3^
Ser294Ala	10.5 (4.17)	1.90 (0.557)	0.182 (0.0900)	>1.00 × 10^3^
Tyr343Ala	3.54 (0.867)	1.29 (0.167)	0.364 (0.101)	>1.00 × 10^3^
Lys345Ala	2.60 (0.854)	12.2 (2.05)	4.68 (0.141)	>1.00 × 10^3^

a The enzymatic activities of mutant Nsp15 variants were measured by monitoring fluorescence changes (excitation at 495 nm and emission at 520 nm) during a 30-min incubation at 25 °C with 150 nM enzyme and increasing concentrations of the fluorogenic substrate (5′-6-FAM-dArUdAdA-6-TAMRA-3′). Values (mean ± SD) of the kinetic parameters *K*_*m*_, *V*_max_, and *k*_cat_ were determined by fitting the data to the Michaelis–Menten equation using GraphPad Prism (v = *V*_max_[S]/(*K*_*m*_ + [S])), with the enzyme concentration (Et) fixed at 0.15 μM. The catalytic efficiency (*k*_cat_/*K*_*m*_) value (mean ± SD) was subsequently calculated.

b The enzymatic activities of mutant Nsp15 variants were measured under similar conditions using 150 nM enzyme, 1.2 μM substrate, and increasing concentrations of KCO237 or KCO251. IC_50_ values (mean ± SD) were determined by fitting dose–response curves to a four-parameter logistic model (log[inhibitor] *versus* response) in GraphPad Prism: Y = bottom + (top − bottom)/(1 + 10 ((LogIC_50_ − X) × HillSlope)).

### Antiviral efficacy and cytotoxicity of potent Nsp15 inhibitors

**Figure 8:**
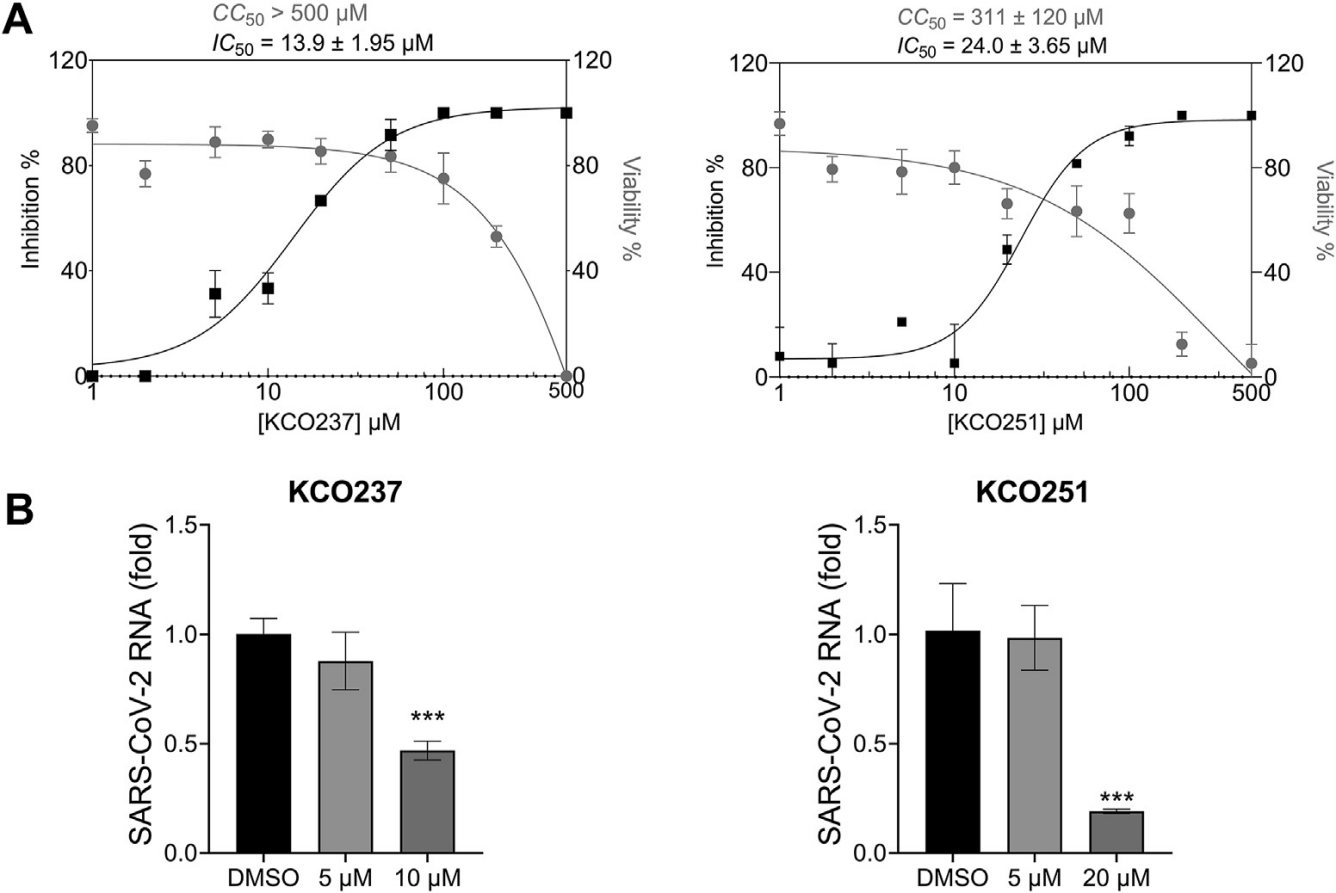
**Activity of SARS-CoV-2 Nsp15 inhibitors in cells**. *A*, plots showing the effect of increasing concentrations of Nsp15 inhibitors on viral inhibition (*gray curve*) and cell viability (*black curve*) relative to DMSO control sample. To determine viral inhibition, viral titers were measured by plaque assay following a 24 h infection of Vero E6 cells with SARS-CoV-2 (MOI = 0.1) in the presence of increasing concentrations of the Nsp15 inhibitors. Cell viability was determined by measuring the intracellular concentrations of ATP in the presence of similar increasing concentrations of Nsp15 inhibitors. IC_50_ and CC_50_ values (mean ± SD) were calculated by fitting dose–response curves to [inhibitor] *versus* response–variable slope curves (four parameters) in GraphPad Prism Y = bottom + (top–bottom)/(1+(IC_50_/X) HillSlope) in GraphPad Prism. *B*, bar graphs showing the fold of viral gene expression in VERO E6 cells in the absence and presence of different doses of Nsp15 inhibitor. SARS-CoV-2 RNA and human GAPDH mRNA (housekeeping gene) were measured by qRT–PCR following a 24 h infection of Vero E6 cells with SARS-CoV-2 (MOI = 0.1). Viral gene expression fold was calculated using the 2(−ΔΔCT) method. Error bars represent the standard deviation of duplicate samples. ∗∗ denotes *p* < 0.01 and ∗∗∗ denotes *p* < 0.001 significance using a one-tailed Student’s *t* test *versus* the DMSO control. DMSO, dimethyl sulfoxide; MOI, multiplicity of infection; Nsp15, nonstructural protein 15; qRT–PCR, quantitative RT–PCR; SARS-CoV-2, severe acute respiratory syndrome coronavirus 2.

FRET-based inhibition assays showed that compounds KCO236, KCO237, and KCO251 are the most potent inhibitors of SARS-CoV-2 Nsp15. To establish an approximate selectivity index (SI) for each inhibitor, we ran two parallel dose titrations: a viral plaque assay to measure the inhibitors' effect on SARS-CoV-2 replication in VERO cells and a luminescent 3-[4,5-dimethylthiazol-2-yl]-2,5 diphenyl tetrazolium bromide cell–based viability assay to measure their cytotoxic effects on uninfected VERO cells (Fig. 8*A*; Fig. S12). The IC_50_ values for KCO237 and KCO251 were 14 μM and 24 μM, whereas the CC_50_ values for the same compounds were >500 μM and >300 μM, respectively. Then, we calculated the SI of each inhibitor by dividing the CC_50_ over IC_50_ values. The SI values for KCO237 and KCO251 were ∼78 and ∼13, respectively. Finally, we used the quantitative RT–PCR (qRT–PCR) to measure the intracellular SARS-CoV-2 RNA in the absence or the presence of each compound. Both compounds significantly reduced viral RNA in a dose-dependent manner (Fig. 8*B*; Fig. S12). These findings show that compounds KCO237 and KCO251 inhibit SARS-CoV-2 replication at nontoxic concentrations.

## Discussion

### Kinetic properties

Although SARS-CoV-2 Nsp15 does not directly participate in RTC formation, it attenuates the host cell’s IFN-1-mediated antiviral response. Its involvement ultimately facilitates viral replication (21, 22). This critical role in the SARS-CoV-2 infection process identifies Nsp15 as a potential antiviral target (29). We expressed the enzyme in BL21 (DE3) cells and purified it to high purity using a Ni–NTA–agarose column, as confirmed by SDS-PAGE. SDS-PAGE also verified the molecular weight of the His-tagged Nsp15 as 41 kDa (34). We monitored enzymatic activity by measuring changes in fluorescence. The enzyme cleaves the substrate oligo 5′-6-FAM-dArUdAdA-6-TAMRA-3′, separating the FAM fluorophore from the TAMRA quencher, resulting in increased fluorescence. None of the assay conditions, including the enzyme or buffer, interfered with fluorescence measurement. To convert relative fluorescence changes into rates of hydrolyzed substrate, we used a standard curve generated from unquenched substrate, 5′-6-FAM-dArUdAdA-3′. Previous studies indicate that this substrate is among the most sensitive for detecting Nsp15 activity (35). We plotted the concentration of intact substrate against the amount of cleaved product and fitted the data to a Michaelis–Menten hyperbolic curve. From this, we determined the kinetic parameters *K*_*m*_, *V*_max_, *k*_cat_, and *k*_cat_/*K*_*m*_. We compared the kinetic parameters reported in the current study with those from previous studies using the same substrate. A recent study reported a ∼10-fold higher *K*_*m*_ value (7.4 μM) for SARS-CoV-2 Nsp15 compared with our result (36). Another group observed a significantly lower *k*_cat_ (0.0071 s^−1^) than what we report here (10.4 min^−1^ = 0.173 s^−1^) (37). A separate study used the same substrate to examine the kinetic properties of Nsp15 from other coronaviruses, estimating a much lower *V*_max_ (1.25 nMs^−1^) for Middle East respiratory syndrome-CoV Nsp15 compared with our value for SARS-CoV-2 Nsp15 (1.62 μM min^−1^ = 27 nMs^−1^) (38). One report described a markedly higher *K*_*m*_ (169 μM) for SARS-CoV Nsp15 (39). In addition, another study reported a *k*_cat_ of 0.2 s^−1^ and a *K*_*m*_ of 24 μM for mouse hepatitis virus nsp15 (40). While the *k*_cat_ aligns closely with our findings, the *K*_*m*_ is notably higher. Notably, although these studies used a substrate similar to ours, their assays were conducted in the absence of Mn^2+^, a known activator of Nsp15 (41). The presence of Mn^2+^ in our assays likely accounts for the enhanced enzymatic activity observed. Other investigations have employed various substrates to characterize SARS-CoV-2 Nsp15 kinetics. While substrate differences complicate direct comparisons, the similar lengths and sequences of these oligos allow for meaningful relative assessments. Two studies reported *K*_*m*_ and *V*_max_ values for SARS-CoV-2 Nsp15 of (2.1 μM, 2.7 μM/min^-1^) and (3 μM, 0.3 μM/min^-1^), respectively (29, 30), which are in close agreement with our findings. Notably, both studies conducted their assays in the presence of Mn^2+^. Overall, the kinetic parameters obtained in the current study suggest higher enzymatic activity because of Mn^2+^ inclusion.

### Virtual docking

The original crystal structure used for virtual docking included interactions with water molecules and phosphate ions. However, these were excluded from the docking procedure in the current study, which explains their absence in our model. While the crystal structure revealed an interaction between tipiracil and the residue Gln245, our docking model did not detect this specific interaction. Nonetheless, it predicted a strong polar interaction in the vicinity of Gln245, suggesting a potential role for this residue in tipiracil binding. Similarly, the docking model did not identify the hydrogen bond between tipiracil and His250 that was observed in the crystal structure. It is important to note that the imidazole group was kept in its deprotonated form in our docking experiments, as the pH was set to 8.0. This deprotonation may explain the absence of hydrogen bonding. In contrast, the crystallography study was conducted at pH 6.4, which maintained the imidazole group in a protonated state, thereby enabling hydrogen bonding. Consistent with the crystal structure, the docking model highlights the critical role of Ser294 within the uridine-binding site.

### IC_50_ determinations

The results of the current study demonstrate that the rhodanine head group exerts stronger inhibition than the thiazolidinedione head group, suggesting that rhodanine engages in more robust interactions with the binding site. The *pK*_a_ values of rhodanine and thiazolidinedione indicate that rhodanine is a stronger proton donor, increasing its likelihood of interacting effectively within the active site (42, 43). A previous study reported similar findings (44), investigating the interactions of thiazolidinedione and rhodanine groups with the active site of human pancreatic alpha-amylase. In the study, atom O_2_ in thiazolidinedione and O_4_ in rhodanine, each formed two hydrogen bonds with residues Arg197 and His299. A third hydrogen bond was formed by the -N3H3 group of either thiazolidinedione or rhodanine with Asp197. Despite these structural similarities, rhodanine demonstrated more potent inhibition than thiazolidinedione. The study showed that the -N3H3 group of rhodanine forms a stronger and more stable hydrogen bond with Asp197, resulting in a 200-fold higher binding constant for rhodanine compared with thiazolidinedione, which forms a weaker bond. This significant difference explains rhodanine’s superior inhibitory effect. In the current docking experiments, thiazolidinedione and rhodanine displayed comparable interaction patterns: both formed hydrogen bonds with Ser294 through their -N3H3 group and two additional hydrogen bonds through O_2_ (thiazolidinedione) or O_4_ (rhodanine) with His250 and Ser294. It is reasonable to predict that rhodanine forms a more stable hydrogen bond between its -N3H3 and the residue Ser294 and thus binds more tightly than thiazolidinedione. This prediction is in agreement with the observed stronger inhibitory behavior of rhodanine. The compounds KCO250 and KCO238 confirm the essential role of these hydrogen bonds in inhibiting the enzyme. Compounds KCO250 and KCO238 support this conclusion; both have a methyl group attached to the -N3 of the thiazolidinedione head group, which disrupts hydrogen bonding with Ser294 and reduces inhibitory activity. Conversely, docking of the stronger inhibitors in this study reaffirms the critical role of hydrogen bonding with Ser294. Only two compounds, KCO230 and KCO236, deviate from this trend. KCO230 contains a rhodanine head group and shows full interaction with His250 and Ser294 but weaker inhibition than structurally similar analogs. KCO236, which has a thiazolidinedione head group, displays an irregular interaction between its S1 group and Ser294. Previous studies have attributed such discrepancies to inherent limitations in docking simulations, especially when modeling large, solvent-exposed active sites (45).

The current study indicates that the two-ring naphthalene base results in stronger inhibition than the three-ring anthracene base. In the docking experiments, the naphthalene or anthracene base occupied the same region as tipiracil’s iminopyrrolidine ring. In these simulations, the naphthalene, anthracene, and iminopyrrolidine rings were surrounded by solvent, which contrasts with the crystal structure, where a phosphate ion shields the iminopyrrolidine ring from solvent exposure. Notably, water and phosphate ions were excluded from the docking experiment. This omission makes it challenging to accurately predict the interactions of these nonpolar moieties under real assay conditions. Despite this limitation, it is reasonable to suggest that hydrophobicity governs how nonpolar moieties interact with water and the binding site. Lower hydrophobicity generally leads to fewer unfavorable interactions with water and dissolved ions. Fewer unfavorable interactions, in turn, enhance molecular stability within the uridine-binding site and strengthen inhibition. Naphthalene is smaller than anthracene and, as shown in the docking experiments, has less solvent exposure. It is also less hydrophobic (logP = 3.15) than anthracene (logP = 4.3) (46). Therefore, it is reasonable to infer that the naphthalene moiety experiences fewer unfavorable interactions under assay conditions than anthracene, contributing to stronger and more stable binding. This likely explains why naphthalene-based molecules are more potent inhibitors. Similarly, compounds with quinoline or isoquinoline bases exhibit even stronger inhibition than those with naphthalene. Quinoline and isoquinoline have lower hydrophobicity values (logP = 1.94 and 2.05, respectively) because of N atoms that introduce polarity and reduce overall hydrophobicity. This polarity enhances binding affinity at the active site. The compound KCO036 contradicts the previous argument. KCO036 has a small benzene ring base with relatively low hydrophobicity (logP = 1.94). Despite this, KCO036 is a weak inhibitor. The less effective thiazolidinedione head group may explain this low inhibitory effect.

Previous studies have reported a limited number of compounds capable of inhibiting SARS-CoV-2 and other coronavirus Nsp15 enzymes at low concentrations. In the current study, we present several thiazolidinedione and rhodanine analogs that inhibit nsp15 at micromolar and submicromolar concentrations. Notably, compounds KCO236, KCO237, and KCO251 inhibit SARS-CoV nsp15 at submicromolar levels, comparable to the potency of several previously reported small-molecule inhibitors (47). In addition, compounds such as KCO048 and KCO249 inhibit the enzyme at low micromolar concentrations, similar to exebryl-1 (IC_50_ = 1.28 μM) (23). It is important to note that these comparisons are somewhat valid, as the referenced studies used a similar substrate and were conducted in the presence of Mn^2+^. Other studies have reported Nsp15 inhibitors with relatively higher IC_50_ values than the thiazolidinedione and rhodanine analogs. These include tipiracil (IC_50_ = 7.5 μM) (27), hexachlorophene (IC_50_ = 1 μM), and IPA-3 (IC_50_ = 7 μM) (29). Still others, such as NSC95397 (IC_50_ = 43 μM) (30) and CO-02 (IC_50_ = 43.5 μM) (48), show significantly higher IC_50_ values. It is essential to recognize that later studies employed different substrates and enzyme concentrations than ours. In these cases, one should interpret IC_50_ comparisons cautiously, as assay conditions significantly affect their values. For example, tipiracil did not inhibit Nsp15 under our assay conditions. However, when we applied the conditions of the previous study, we were able to detect tipiracil inhibition. One can attribute this apparent discrepancy in tipiracil’s behavior to the differences in buffer, temperature, substrate, assay sensitivity, and protein concentration. The final concentration of Nsp15 in the mentioned study is almost 15-fold less than the concentration in our assay. Expectedly, the higher enzyme concentration we used in our study required more tipiracil to inhibit Nsp15. In addition to differences in protein concentration, the structure of the substrates in the two assay conditions is inherently different. Tipiracil could differentially interact with the binding in the presence of each substrate. Nevertheless, KCO237 and KCO251 inhibited Nsp15 more potently than tipiracil under these conditions.

### Inhibition mechanism

Kinetic inhibition assays can provide critical insights into the inhibitor–enzyme interaction. However, the precise location of inhibitor binding remains unclear in the absence of structural evidence. In a recent study, the compound CID5675221, a rhodanine scaffold–based molecule, inhibited Nsp15 through a mixed inhibition mechanism (29). This study employed molecular modeling and molecular dynamics simulations to investigate the interactions of several compounds with nsp15-binding sites. It predicted the presence of an allosteric site located deeper within the protein structure. This site lies between residues Asn200, Asn273, Ser274, and Tyr279. Docking the compound to both active and allosteric sites suggested a noncompetitive inhibition mechanism. The docking model showed stronger binding affinity at the allosteric site. However, the molecular dynamics simulations were more consistent with solution-phase data and indicated mixed inhibition behavior. The study concludes that CID5675221 inhibits Nsp15 by binding to an allosteric site. The inhibitors examined in the current study are also rhodanine scaffold–based compounds. They inhibit Nsp15 through a mixed inhibition mode. Therefore, it is reasonable to infer that these compounds inhibit Nsp15 by targeting the allosteric site, similar to CID5675221. However, as with the MOE study, we cannot exclude the possibility of these molecules binding to the active site.

The same study indicated that the compound IPA3 irreversibly inhibited Nsp15 (29). Evidence suggested that the reactive disulfide group of IPA3 might act as a Michael acceptor, covalently interacting with the thiol group of a cysteine in the binding site. The inhibitors in our investigation contain an olefin moiety connecting the thiazolidinedione and rhodanine groups. This electron-deficient diene system can act as a Michael acceptor, forming covalent bonds with either the thiol of Cys293 or the amine of Lys345. This covalent bonding significantly affects the reversibility of the inhibition. Nsp15, preincubated with a high concentration of inhibitor, restored its activity after substantial dilution of the inhibitor, clearly demonstrating the reversible nature of inhibition by KCO237 and KCO251. When the olefin moiety of KCO237 was saturated (analog KCO376), the analog lost its inhibitory effect. While this outcome does not rule out covalent interaction with Cys293 or Lys345, it emphasizes the critical role of the double bond in the inhibition mechanism. Olefins' double bond appears to be essential to properly position the inhibitor in the binding site. Reversible covalent inhibitors usually take a long time to establish their covalent modification equilibrium because they bind in two steps: a fast reversible first step and a slower covalent second step. This inhibition mechanism is time dependent, and the IC_50_ of the inhibitor most properly drops as the reaction progresses. We traced the IC_50_s of KCO237 and KCO251 and noticed that they remained constant over the time of the assay. The stability of IC_50_ over time implies that KCO237 and KCO251 bind to Nsp15 in a reversible manner. Taken together, these results suggest that the olefin is critical for the inhibition action but most likely not through covalent interaction.

Changing the secondary amine in the rhodanine ring to a tertiary amine caused the analogs KCO238 and KCO250 to lose their inhibitory effect. CID5675221 is a rhodanine scaffold–based analog that contains a tertiary amine. In contrast, KCO237, which retains a secondary amine in its rhodanine scaffold, exhibits potent inhibitory activity. Therefore, it is reasonable to predict that CID5675221 is a weaker inhibitor than KCO237. However, the assay conditions in this study differ from those in the current study. As previously mentioned, this makes it difficult to directly compare the IC_50_ values of CID5675221 and KCO237. To enable a relatively meaningful comparison, we used the Cheng–Prusoff equation to calculate the predicted *K*_*i*_ values of these compounds (Table S3) (49, 50). These calculations were performed under the assumption of noncompetitive inhibition. The predicted *K*_*i*_ for CID5675221 was approximately 20 μM, which is significantly higher than the predicted value for KCO237 (0.23 μM). This suggests that KCO237 is potentially a stronger noncompetitive inhibitor than CID5675221. If KCO237 binds with such high affinity to the allosteric site, one would expect it to follow a noncompetitive mechanism in solution studies. However, solution inhibition data indicated that KCO237 fits better with a mixed inhibition mechanism. Altogether, these results support the idea that KCO237 binds to the active site, although allosteric binding cannot be entirely ruled out.

There are several possible explanations for why an active site–directed inhibitor may exhibit noncompetitive behavior (51, 52). Some of these scenarios could apply to Nsp15. The presence of two slowly interconverting enzyme conformations can account for the apparent mixed inhibition observed with active site–directed inhibitors. In this conformational selection model, an inhibitor preferably targets one of the two conformations more effectively than the other. A recent study investigated the single-turnover kinetics of SARS-CoV-2 Nsp15 and reported biphasic behavior. It identified *k*_c,1_ and *k*_c,2_ values of 0.36 min^-1^ and >1 min^-1^, respectively, in the presence of M^2+^ (53). The study proposed that the existence of two slowly reversible conformations of Nsp15 explains this biphasic behavior. In this model, the inhibitor acts more strongly on one of the conformers, and the slow interconversion between less- and more-inhibited states results in a mixed inhibition pattern. Other researchers have suggested that nucleases generally interact with their large macromolecular substrates at multiple sites beyond the active site where the scissile bond resides. In this model, a small inhibitor binding at the active site may interfere with substrate interactions but cannot fully overcome the stabilization and recognition processes occurring outside the active site. Consequently, the substrate may still bind even in the presence of an inhibitor at the active site, giving rise to apparent mixed inhibition (51, 52). However, in the absence of structural data for Nsp15 bound to a substrate longer than a dinucleotide, it is difficult to apply this model with confidence (54). A crystal structure of RNase A in complex with the oligonucleotide d(ApTpApApG) revealed multiple interactions with ribose bases located two nucleotides away from the scissile bond. That study proposed that the enzyme–nucleic acid complex utilizes these interactions primarily for stabilization (51, 52, 53). Similar interactions have been observed in the active site of Nsp15 (27, 37, 55). One study showed that the interaction of residue Trp333 in Nsp15 serves as a main stabilizing force for the ribose moiety positioned two nucleotides from the scissile phosphate. This interaction did not contribute to substrate specificity (54). It is important to note that these enzyme–substrate interactions occur near the active site; however, it is also plausible that similar stabilizing interactions exist outside the active site to maintain the integrity of the enzyme–substrate complex.

### Mutation analysis

Mutating His250A retained ∼4% of the activity. This significant loss agrees with previous mutation analysis studies, which reported that mutation of His250 caused a complete loss of activity (38, 56, 57, 58). The minor but detectable activity aligns with one report showing that His250Ala Nsp15 retained low, nonspecific activity (34). The substantial loss of activity in the His250Ala mutant confirms the role of this histidine in the catalytic triad of Nsp15. The study demonstrated that His250 acts as a base to activate the ribose 2′OH, facilitating nucleophilic attack (56, 59). The Ser294A mutant retained partial activity (∼12%), consistent with earlier studies showing that mutating Ser294 reduces but does not abolish Nsp15 activity. This finding also supports the conclusion that Ser294 is essential for uridine specificity, though not required for RNA cleavage activity (34, 59). Studies have reported conflicting effects of the Tyr343Ala mutation. The current study observed ∼10% activity retention. One report found no detectable activity for Tyr343Ala (58), whereas another study reported a doubling of activity in the mutant compared with wildtype Nsp15 (34). Yet another study concluded that Tyr343 is critical for proper ribose orientation in the active site but not essential for substrate binding (56). Last, the Lys345Ala mutant retained ∼82% of its activity, suggesting that this residue is involved in tipiracil binding, as shown by crystal structures, but not in substrate binding (27). The reduced inhibitory effect of KCO237 on the residual activities of the Tyr343Ala, His250Ala, Ser294Ala, and Lys345Ala mutants indicates the importance of these residues in inhibitor binding at the uridine-binding site. Nevertheless, the possibility that the inhibitor binds through an unmutated allosteric site remains valid.

### Bioavailability and efficacy

We employed SwissADME to evaluate the drug likeness and oral bioavailability potential of the rhodanine compounds (60). None of these compounds violated Lipinski’s Rule of Five or Veber’s criteria (Table S4). We used the CC_50_ and IC_50_ values to calculate the SI of potent rhodanine inhibitors. KCO237 and KCO251 exhibited high SI values (>10), indicating strong antiviral activity with minimal toxic effect. Overall, KCO237 and KCO251 possess the necessary properties to function as effective and orally bioavailable treatment of SARS-CoV-2.

In conclusion, this study explores Nsp15 as a potential drug target for treating SARS-CoV-2 and other emerging coronavirus infections. We utilized molecular docking to design structure-based inhibitors and subsequently applied an FRET-based screening assay to validate and characterize them. The study identifies a novel class of potent Nsp15 inhibitors, comprising a quinoline, isoquinoline, and naphthalene base linked to thiazolidinedione and rhodanine head group.

## Experimental procedures

### Materials

We outsourced the synthesis and cloning of wildtype SARS-CoV-2 Nsp15 and the mutants Ser294Ala, Lys345Ala, His250Ala, Lys290Ala, Ser94Ala/Cys293Ala, and Lys345Ala/Tyr343Ala into the bacterial expression plasmid pET-15b (+) with an N-terminal His-tag through GeneScript. The expressed recombinant proteins were purified using Ni–NTA–agarose resin (ThermoFisher). The oligonucleotide 5′-6-FAM-dArUdAdA-6-TAMRA-3′ was purchased from Integrated DNA Technologies. FRET assays were performed using black 96-well flat-bottom plates (Corning). All organic solvents used were reagent grade. Spectroscopic-grade solvents were used as received. TLC was conducted on 5 × 2.5 cm silica gel GF254 plates precoated on aluminum backing. TLC spots were visualized under UV light at 254 nm and further developed using chemical staining with potassium permanganate (KMnO_4_). Reagents, including starting material aldehydes for the Wittig reaction, bromosubstituted starting materials for Heck reactions with extended aldehydes, thiazolidine-2,4-dione, rhodanine, K_2_CO_3_, and all other solvents, were purchased from TCI America and Santa Cruz Biotechnology. All reagents were used without further purification.

### Synthesis of thiazolidinedione and rhodanine analogs

Proton (^1^H) NMR spectra were acquired at 298 K using a JEOL spectrometer operating at 400 MHz. Chemical shifts are reported in ppm (δ), using dimethyl sulfoxide (DMSO-d_6_) as the reference solvent at δ 2.50 ppm. Coupling constants (J values) are reported in hertz (Hz). To improve analyte solubility and prevent freezing of DMSO-d_6_, a 25% (v/v) mixture of DMSO-d_6_ and CCl_4_ was used instead of pure DMSO-d_6_.

### General procedure for the synthesis of aldehydes and Knoevenagel condensation

Extended aldehydes with general formula 1 were prepared using either the Wittig or Heck reaction, as previously reported in the literature (61, 62). The Wittig reaction was used to synthesize aldehydes for compounds 1, 3, and 7, whereas the Heck reaction was employed for aldehydes used in compounds 9, 11, and 12 (Fig. 2*A*). The appropriate aldehyde (1 mmol, 1 eq) was dissolved in ethanol (15 ml), followed by the addition of thiazolidinedione or rhodanine (1.5 eq). Morpholine (1.5 eq) was then added, and the reaction mixture was stirred at 60 °C for 8 h. The resulting precipitate was filtered and washed with ethanol to remove unreacted aldehydes. The final product was dried under vacuum, yielding a solid with a yield of 50% to 76%.

### Methylation of thiazolidinedione analogs

To synthesize KCO250, we combined 5-(3-naphthalen-1-yl-allylidene)-thiazolidine-2,4-dione (0.5 mmol) with potassium carbonate (5 mmol, 10 eq) and dried *N*,*N* dimethylformamide (2 ml) in a 40 ml reaction vial. The mixture was stirred gently before adding an excess of iodomethane (1.5 mmol, 3 eq). The vial was then sealed, and the reaction mixture was stirred at 90 °C for several minutes until the starting material was fully consumed. The reaction was quenched by diluting the mixture with 20 ml of water, leading to product precipitation. The precipitate was collected by suction filtration and recrystallized from an appropriate solvent. A similar procedure was used to methylate the anthracene analog KCO35 (Fig. 2*A*).

### Characterization of rhodanine analogs

The thiazolidinedione analogs were characterized by ^1^H NMR spectroscopy (Figs. 2*B* and S4) as follows:

KCO231 (EXP 16.14): ^1^H NMR (400 MHz, DMSO-D_6_) δ 8.24 (d, J = 8.4 Hz, 1H), 8.08 (d, J = 15.0 Hz, 1H), 7.86 (dd, J = 11.5, 7.8 Hz, 3H), 7.70–7.63 (m, 1H), 7.58–7.41 (m, 3H), 6.83 (dd, J = 15.0, 11.4 Hz, 1H).

KCO230 (EXP-9.127): ^1^H NMR (400 MHz, DMSO-D_6_) δ 8.51 (s, 1H), 8.39–8.21 (m, 3H), 8.04 (dd, J = 7.1, 2.6 Hz, 2H), 7.70 (dd, J = 11.5, 0.9 Hz, 1H), 7.57–7.39 (m, 4H), 6.71 (dd, J = 15.4, 11.5 Hz, 1H).

KCO234 (II3-16): ^1^H NMR (400 MHz, DMSO-D_6_) δ 9.26 (s, 1H), 8.98 (s, 1H), 8.32 (d, J = 8.4 Hz, 1H), 8.17–8.06 (m, 2H), 7.90–7.81 (m, 1H), 7.71 (t, J = 7.3 Hz, 1H), 7.63 (d, J = 11.3 Hz, 1H), 7.12 (dd, J = 15.1, 11.4 Hz, 1H).

KCO235 (SH5-56): ^1^H NMR (400 MHz, DMSO-D_6_) δ 12.45 (s, 1H), 9.75 (s, 1H), 8.54 (d, J = 5.6 Hz, 1H), 8.38 (d, J = 14.9 Hz, 1H), 8.17 (d, J = 7.3 Hz, 1H), 7.96 (d, J = 8.2 Hz, 1H), 7.83 (d, J = 5.3 Hz, 1H), 7.77 (t, J = 7.8 Hz, 1H), 7.72–7.64 (m, 1H), 7.10 (dd, J = 14.9, 11.4 Hz, 1H).

KCO238 (EXP 16.20): ^1^H-NMR (400 MHz, DMSO-D_6_) δ 8.51 (s, 1H), 8.29 (dd, J = 9.6, 7.6 Hz, 3H), 8.14–8.01 (m, 2H), 7.98 (d, J = 11.4 Hz, 1H), 7.61–7.43 (m, 4H), 6.70 (dd, J = 15.4, 11.4 Hz, 1H), 3.18 (s, 3H).

KCO250 (EXP 16.38): ^1^H-NMR (400 MHz, DMSO-D_6_) δ 8.26 (d, J = 8.4 Hz, 1H), 8.16 (d, J = 14.9 Hz, 1H), 7.91 (d, J = 7.2 Hz, 1H), 7.80 (dd, J = 11.4, 0.7 Hz, 2H), 7.59–7.40 (m, 4H), 6.89 (dd, J = 14.9, 11.5 Hz, 1H), 3.12 (s, 3H).

KCO249 (EXP 16.31): ^1^H-NMR (400 MHz, DMSO-D_6_) δ 8.26 (d, J = 8.5 Hz, 1H), 8.15 (d, J = 14.9 Hz, 1H), 7.92 (d, J = 7.3 Hz, 1H), 7.86 (dd, J = 7.4, 4.6 Hz, 2H), 7.60–7.40 (m, 4H), 6.91 (dd, J = 14.9, 11.6 Hz, 1H). ^13^C NMR (101 MHz, DMSO-D_6_) δ 194.84, 168.99, 141.10, 133.95, 132.85, 132.23, 131.44, 130.51, 128.99, 127.85, 127.10, 126.39, 125.86, 124.91, 123.64.

KCO237 (SH5-58: ^1^H-NMR (400 MHz, DMSO-D_6_) δ 8.89 (d, J = 4.7 Hz, 1H), 8.37 (d, J = 8.0 Hz, 1H), 8.21 (d, J = 14.7 Hz, 1H), 8.08–7.91 (m, 2H), 7.77 (t, J = 6.9 Hz, 1H), 7.66 (t, J = 7.7 Hz, 1H), 7.48 (d, J = 11.3 Hz, 1H), 7.30 (dd, J = 14.8, 11.6 Hz, 1H).

KCO251 ((II3-25): ^1^H-NMR (400 MHz, DMSO-D_6_) δ 8.88 (d, J = 4.6 Hz, 1H), 8.37 (d, J = 8.2 Hz, 1H), 8.20 (d, J = 14.9 Hz, 1H), 8.02 (d, J = 7.8 Hz, 1H), 7.96 (d, J = 4.7 Hz, 1H), 7.84 – 7.71 (m, 1H), 7.66 (t, J = 7.0 Hz, 1H), 7.47 (d, J = 11.5 Hz, 1H), 7.30 (dd, J = 14.9, 11.5 Hz, 1H).

KCO236 (SH5-57): ^1^H-NMR (400 MHz, DMSO-D_6_) δ 9.26 (s, 1H), 8.48 (d, J = 5.7 Hz, 1H), 8.08 (d, J = 8.4 Hz, 2H), 8.03 (dd, J = 8.7, 1.3 Hz, 1H), 7.79 (d, J = 5.8 Hz, 1H), 7.48 (d, J = 11.4 Hz, 1H), 7.41 (d, J = 15.2 Hz, 1H), 7.14 (dd, J = 15.2, 11.4 Hz, 1H).

KCO35 (EXP 9.104): ^1^H-NMR (400 MHz, acetone-D_6_) δ 8.58 (s, 1H), 8.33 to 8.37 (m, 2H), 8.26 (d, J = 15.6 Hz, 1H), 8.04 to 8.13 (m, 2H), 7.81 to 7.87 (m, 1H), 7.51 to 7.58 (m, 4H), 6.77 (dd, J = 15.5, 11.3 Hz, 1H).

KCO48 (EXP 9.121): ^1^H-NMR (400 MHz, DMSO-D_6_) δ 9.86 (d, J = 7.3 Hz, 1H), 9.70 (s, 0H), 8.88 (d, J = 4.6 Hz, 1H), 8.81 (t, J = 4.8 Hz, 1H), 8.44 (d, J = 15.8 Hz, 1H), 8.15 to 8.28 (m, 2H), 8.04 to 8.07 (m, 1H), 6.89 to 7.03 (m, 2H).

5-(3-Phenyl-allylidene)-thiazolidine-2,4-dione (KCO36, EXP 9.105): ^1^H-NMR (400 MHz, DMSO-D_6_) δ 7.47 to 7.49 (m, 2H), 7.38 (dd, J = 11.4, 0.7 Hz, 1H), 7.24 to 7.33 (m, 3H), 7.05 (d, J = 15.3 Hz, 1H), 6.68 (dd, J = 15.3, 11.4 Hz, 1H).

### SARS-CoV-2 Nsp15 active-site modeling

We identified the active site of SARS-CoV-2 Nsp15 using five recently published X-ray crystal structures (PDB_IDs: 6WLC, 6WXC, 6X1B, 6X4I, and 7K1L). Structural alignment of these crystal structures showed minimal backbone displacement, indicating overall structural consistency. For docking simulations, we selected the crystal structure of Nsp15 complexed with tipiracil (PDB_ID: 6WXC) as it contains a ligand bound in the active site. Several in-house synthesized compounds were docked into the Nsp15 active site using the MOE, version 2019.0102 (Chemical Computing Group ULC). Prior to docking, we removed water molecules and anions from the protein structure. Both the enzyme and ligands were protonated at pH 8.0 to match the assay conditions. We employed two docking protocols. In the first, the protein structure was kept rigid while allowing full conformational flexibility of the ligands. In the second protocol, both the enzyme and ligands were treated flexibly, with a 9 Å radius around the ligand-binding site permitted to move during docking. For each compound, 10 docking poses were generated and analyzed for interactions with key amino acid residues in the active site. To select a subset of compounds for biological evaluation, we considered ligand–residue interactions and complementarity with the active-site topology. Docked poses showing one or more interactions with Ser294 were prioritized and included in the final set of candidate inhibitors.

### Protein expression and purification

SARS-CoV-2 pET-15b-nsp15 plasmids were transformed into *E*. *coli* BL21 (DE3)-competent cells and cultured in LB broth supplemented with 100 μg/ml ampicillin. Protein expression and purification were carried out as previously described, with minor modifications (56). Briefly, transformed cultures were grown at 37 °C until they reached an absorbance (at 600 nm) of 0.8 to 1.0. Protein expression was induced by adding 0.1 mM IPTG, and cultures were incubated overnight at 16 °C. Cells were harvested by centrifugation at 6000*g* for 10 min at 4 °C and stored at −80 °C until further use. Cell pellets were thawed and resuspended in 100 ml lysis buffer (20 mM Tris, pH 8.0; 100 mM NaCl; 5 mM β-mercaptoethanol [β-ME]; 5 mM imidazole; and 1 mM PMSF). Cells were lysed *via* sonication at 75% amplitude using a 15:15 s pulse cycle for a total of 2 min. The lysate was clarified by centrifugation at 12,500*g* for 1 h at 4 °C. The supernatant was applied to a Ni–NTA–agarose column pre-equilibrated with three column volumes of lysis buffer. The lysate was allowed to flow through the column under gravity. Nonspecifically bound proteins were removed by washing with five column volumes of wash buffer (20 mM Tris, pH 8.0; 100 mM NaCl; 5 mM β-ME; and 30 mM imidazole). Nsp15 protein was eluted using elution buffer containing 250 mM imidazole (20 mM Tris, pH 8.0; 100 mM NaCl; and 5 mM β-ME). Protein concentration was determined using the Bradford assay, and the purified protein was used in subsequent activity and kinetic assays.

### Nsp15 activity assay

We measured the activity of purified wildtype and mutant SARS-CoV-2 Nsp15 using a previously described FRET-based assay, with minor modifications, on a 96-well platform (40). The substrate used was the oligonucleotide 5′-6-FAM-dArUdAdA-6-TAMRA-3′ (Integrated DNA Technologies). Endonuclease activity was monitored by measuring the increase in fluorescence resulting from the separation of the FAM fluorophore from the TAMRA quencher. To prepare test compounds, lyophilized thiazolidinedione and rhodanine analogs were dissolved in 100% (v/v) DMSO to a final concentration of 10 mM (source plate). Serial dilutions were prepared from the source plate and stored at −80 °C. Each compound was initially tested at a final concentration of 100 μM by adding 1 μl of the 10 mM stock solution to a 100 μl final assay volume. Fluorescence was measured in Microfluor 2 black U-bottom 96-well plates (Fisher Scientific). Compounds were preincubated at room temperature with 300 nM Nsp15 in 50 μl of assay solution for 5 min. Reactions were initiated by adding 50 μl of assay buffer containing 4.2 μM labeled substrate, resulting in a final volume of 100 μl. The final assay buffer composition was 20 mM Tris (pH 8.0), 100 mM NaCl, 10 mM MnCl_2_, 5 mM β-ME, 150 nM Nsp15, 1.2 μM FAM- and TAMRA-labeled substrate, and varying concentrations of inhibitors. Reactions were incubated for 30 min at 25 °C. Fluorescence was measured using a SpectraMax M5 plate reader (Molecular Devices) with excitation at 495 nm and emission at 520 nm. All experiments were conducted in duplicate.

### Kinetics and IC_50_ determinations

To determine the Michaelis–Menten constant (*K*_*m*_), we measured changes in fluorescence using various concentrations of the labeled substrate (0.0, 0.075, 0.15, 0.3, 0.6, 0.9, 1.2, 2.5, and 6 μM). Data were fitted to the Michaelis–Menten equation using GraphPad Prism (version 5.0). The same procedure was used to determine *K*_*m*_ values for the Nsp15 mutants. To identify compounds with strong inhibitory effects, we first screened each compound at 0, 1, 10, and 100 μM concentrations. Subsequently, we assessed Nsp15 activity using a range of inhibitor concentrations (0.38, 1.53, 3.063, 6.125, 12.5, 25, 50, and 100 μM). IC_50_s were calculated by fitting the inhibition data to a four-parameter logistic dose–response curve using GraphPad Prism. To determine the inhibition mechanism and the inhibitor constant (*K*_i_), we measured Nsp15 activity at various substrate concentrations (0.0–6 μM, as listed previously) in the presence of increasing concentrations of each inhibitor (0, 0.5, 2.5, 5, and 10 nM). The resulting data were fitted to competitive, noncompetitive, and mixed inhibition models. Model fit was evaluated using the extra sum-of-squares *F* test in GraphPad Prism to identify the best-fitting model. In addition, inhibition data were plotted as Lineweaver–Burk graphs for visual assessment of kinetic mechanisms. All kinetic and inhibition assays were conducted in duplicate.

### SARS-CoV-2 propagation

We maintained VeroE6/TMPRSS2 cells in Dulbecco’s modified Eagle’s medium (DMEM) supplemented with 2% fetal bovine serum, l-glutamine, and penicillin–streptomycin at 37 °C under a 5% CO_2_/95% air atmosphere. We propagated SARS-CoV-2 (Wuhan-Hu-1) in VeroE6/TMPRSS2 cells at the biosafety level 2 laboratory at the University of Iowa in compliance with approved protocols. Finally, we harvested the SARS-CoV-2 supernatant and stored it in aliquots at −80 °C for further VeroE6/TMPRSS2 cell infection. We determined the titer of the stock by plaque assay on VeroE6/TMPRSS2 cell monolayers.

### VERO cell cytotoxicity assay

Initially, we grew VeroE6/TMPRSS2 cells overnight in 96-well plates at 10,000 cells per well. The next day, we added increasing concentrations of the tested compound (500, 200, 100, 50, 20, 10, 5, 2, and 1 μM) and DMSO as a control. We let the cells incubate with inhibitors for 24 h. After 24 h, we measured the viability of these cells using the CellTiter-Glo Luminescent Cell Viability Assay (Promega), according to the manufacturer’s instructions. We incubated cells in 100 μl of media with 100 μl of CellTiter-Glo reagent, which contains Ultra-Glo luciferase and the substrate luciferin. The luciferase converts luciferin to luminescent oxyluciferin in the presence of ATP. Viable cells produce more ATP and generate a stronger luminescent signal. We mixed samples by shaking for 10 min and measured luminescence by a VICTOR Nivo Plate Reader (Avantor Science Central).

### SARS-CoV-2 plaque assay

To determine the plaque-forming units, we initially grew VeroE6/TMPRSS2 cells overnight in 12-well plates at 400,000 cells per well. The next day, we added 200 μl of 10-fold SARS-CoV-2 supernatant prepared by serial dilution in fresh media. Then, we incubated plates at 37 °C for 1 h and rocked them every 15 min. After this hour, we discarded the viral supernatant and subsequently added 1 ml of 2x DMEM (ThermoFisher) solution mixed with 1 ml of 1.2% agarose (Sigma–Aldrich). After the agarose overlay had set, we added 1 ml of DMEM with 10% fetal bovine serum on top. We maintained cells at 37 °C for 3 days to allow plaque development. At the end of the incubation time, we removed the media and fixed the cells by adding 4% paraformaldehyde directly to the wells and incubating at room temperature for at least 30 min; then we removed the paraformaldehyde solution and stained the cells with a 0.1% crystal violet in 20% methanol. Finally, we counted the number of plaques in each well to determine the viral titer.

To measure the efficacy of the tested inhibitors, we grew VeroE6/TMPRSS2 cells overnight in a 96-well plate as described previously. The next day, we infected the cells with SARS-CoV-2 at a multiplicity of infection = 0.1 in the presence of increasing concentrations of the tested compounds (9 points of 500, 200, 100, 50, 20, 10, 5, 2, and 1 μM) and DMSO as a control. Twenty-four hours after infection, we collected supernatants for virus titering by plaque assay.

## qRT–PCR

We prepared and infected the VeroE6/TMPRSS2 cells in the presence of tested inhibitors as described previously for 24 h. At the end of incubation, we removed the supernatant and extracted total RNA using the RNeasy Plus Mini Kit (Qiagen), following the manufacturer’s protocol. We performed qRT–PCR using PowerUp SYBR Green Master Mix (ThermoFisher) on a QuantStudio 3 Real-Time PCR System (ThermoFisher), following a cycling protocol of 40 cycles at 95 °C for 15 s and 60 °C for 60 s. To measure viral gene expression, we used SARS-CoV-2 Orf1 primers (5′-GGCGAAATACCAGTGGCTTA-3′ and 5′-TGAGTTCACGGGTAACACCA-3′) (63). For host cell gene expression, we used GAPDH primers (5′-GGAAGGTGAAGGTCGGAGTCAACGG-3′ and 5′-CTGTTGTCATACTTCTCATGGTTCAC-3′) (64). Finally, we used the 2(−ΔΔCT) method to calculate gene expression level.

## Data availability

All data associated with this work are contained within this article and supporting information.

## Supporting information

This article contains supporting information (49, 60).

## Conflict of interest

The authors declare that they have no conflicts of interest with the contents of this article.
