# Supplementary material for: First-in-class inhibitors of Nsp15 endoribonuclease of SARS-CoV-2: Modeling, synthesis, and enzymatic assay of thiazolidinedione and rhodanine analogs
Source: J Biol Chem. 2025 Jun 23;301(8):110409. doi: 10.1016/j.jbc.2025.110409 (PMC12303054; doi:10.1016/j.jbc.2025.110409)
Supplement: Supplementary Data 1 [file mmc1.pdf]

Supporting Information

First-in-class inhibitors of Nsp15 endoribonuclease of SARS-CoV-2: modeling, synthesis, and enzymatic assay of thiazolidinedione and rhodanine analogs

Nimer Mehyar, Nosaibah Samman, Shatha Al Gheribi, Abdullah Mashhour, Pearl Chan, Rabih O. Al-Kaysi, Stanley Perlman, Mohamed Boudjelal, and Imadul Islam

Supporting Information

Supplemental Figure 1	Expression and purification of wild-type and mutant variants SARS-CoV-2 Nsp15
Supplemental Figure 2	Control for assay conditions
Supplemental Figure 3	SARS-CoV-2 nsp15 inhibition by in-house compounds KCO035, KCO058, and KCO233
Supplemental Figure 4	NMR profiles of rhodanine analogs
Supplemental Figure 5	Docking of rhodanine compounds in the SARS-CoV-2 Nsp15 active site
Supplemental Figure 6	IC_{50} determination using the FRET-based Nsp15 activity assay
Supplemental Figure 7	Inhibition of wild-type SARS-CoV-2 Nsp15 activity in HEPES buffer assay conditions
Supplemental Figure 8	Effect of time on IC_{50}
Supplemental Figure 9	Kinetic parameters of mutant SARS-CoV-2 Nsp15 variants
Supplemental Figure 10	Mutant Nsp15 variants inhibition by KCO237
Supplemental Figure 11	Mutant Nsp15 variants inhibition by KCO251
Supplemental Figure 12	Activity of analog KCO236 in cells
Supplemental Table 1	Statistical test for inhibition models of analog KCO237
Supplemental Table 2	Statistical test for inhibition models of analog KCO251
Supplemental Table 3	Predicted K_i values for a noncompetitive inhibition model
Supplemental Table 4	Calculated Lipinski's rule of five and Veber's rule for rhodanine analogs

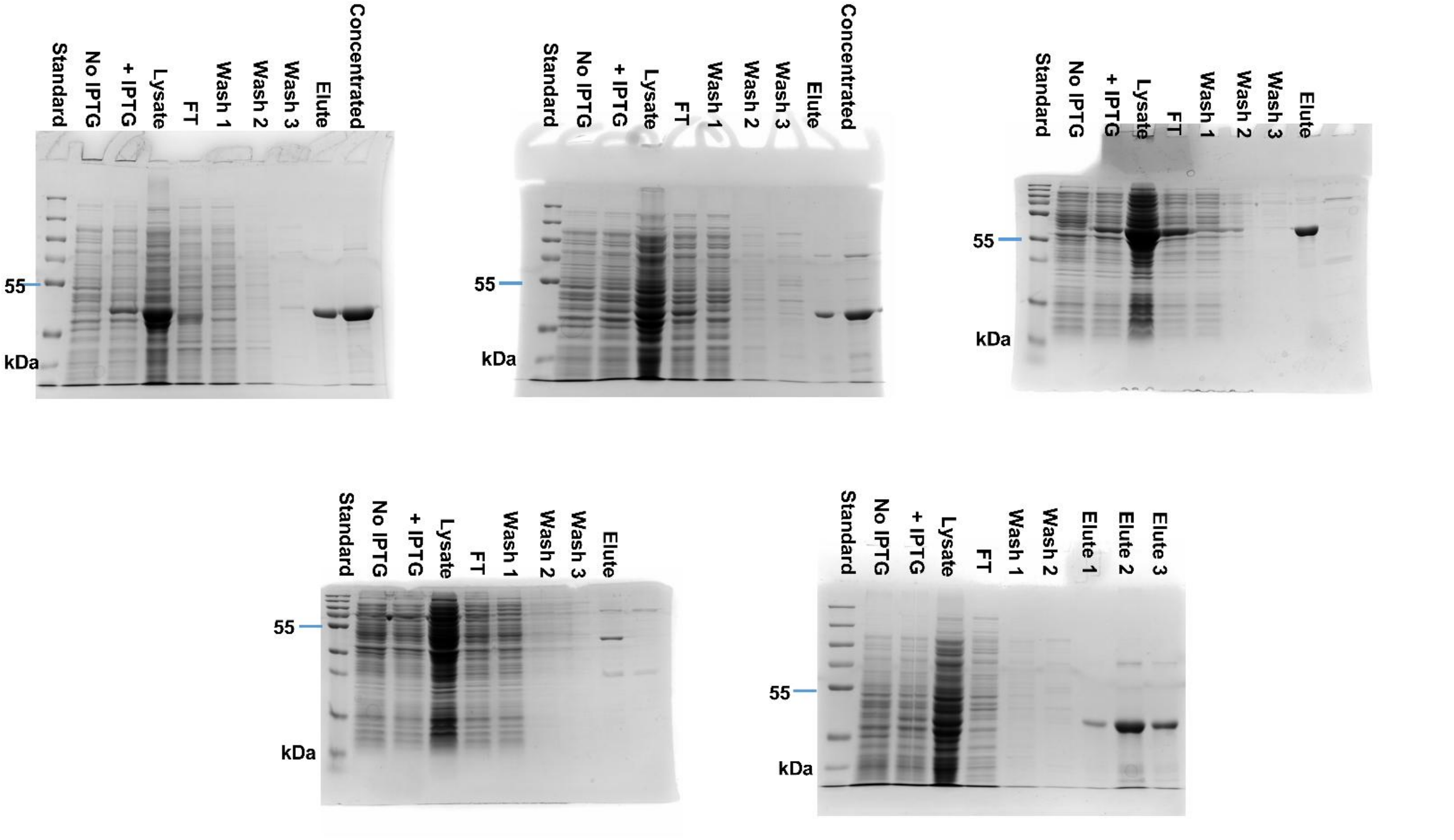

Figure S1. Expression and purification of wild-type and mutant variants SARS-CoV-2 Nsp15. Gels showing purified wild-type, Y343A, H250A, K345A, and S294 variants of SARS-CoV-2 Nsp15 protein, resolved by SDS-PAGE and detected by Coomassie staining. Standards: pre-stained molecular standards (BioRad); No IPTG: whole cell lysate before IPTG addition; +IPTG: whole cell lysate after IPTG addition; Lysate: cell lysate supernatant after sonication at 75% amplitude (15:15 s pulse cycle for a total of 2 min), and centrifugation at 12,500 g (1 h at 4°C); FT (filter through); Washes 1-3: each equals five column volumes of wash buffer (20 mM Tris, pH 8.0; 100 mM NaCl; 5 mM β-ME; and 30 mM imidazole); Elutes: one volume of elution buffer (20 mM Tris, pH 8.0; 100 mM NaCl; and 5 mM β-ME; and 250 mM imidazole).

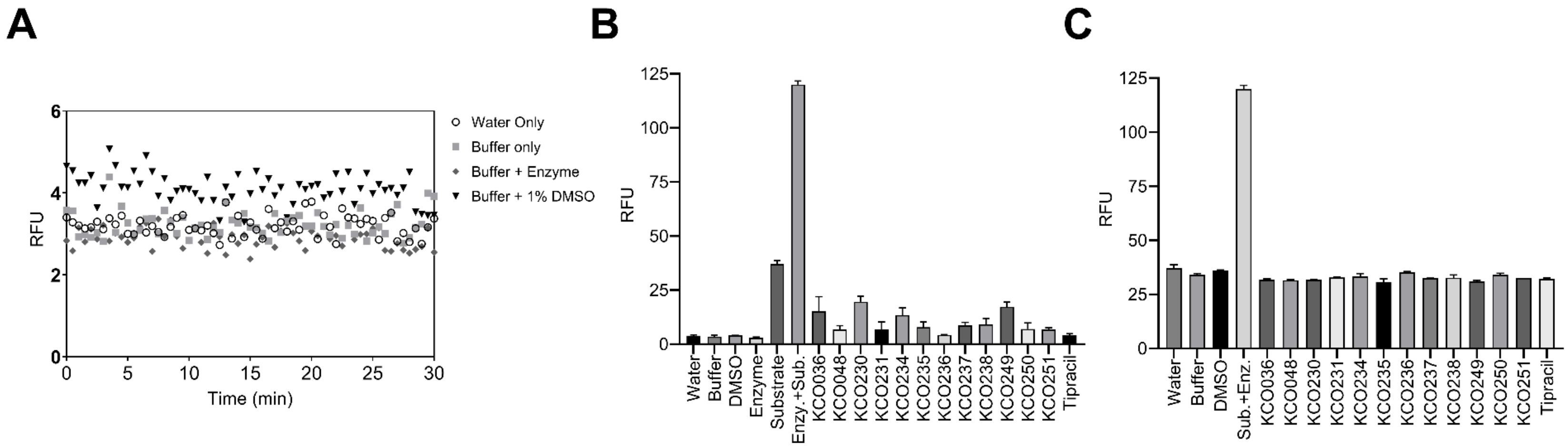

Figure S2. Control for assay conditions. *A,* plot showing the change in fluorescence (excitation 495 nm, emission 520 nm) in absence of the fluorogenic substrate. *B,C* bar graph showing the change in fluorescence (excitation 495 nm, emission 520 nm) of rhodanine compounds in assay buffer is the in absence (B) and presence (C) of the fluorogenic substrate. Reactions were allowed to proceed for 30 min at 25°C.

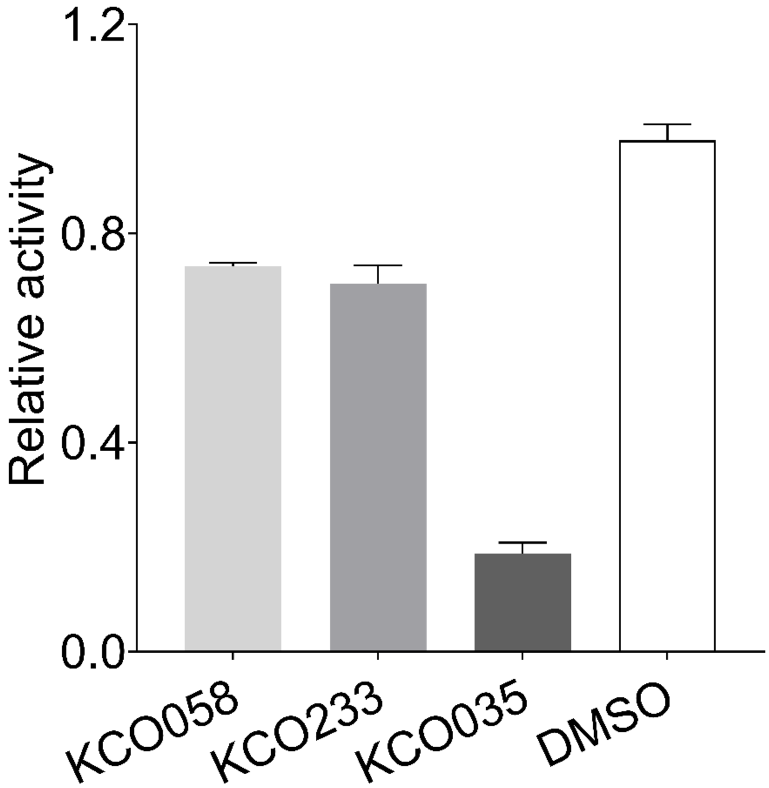

Figure S3. SARS-CoV-2 nsp15 inhibition by in-house compounds KCO035, KCO058, and KCO233. Bar graph showing the change in fluorescence (excitation 495 nm, emission 520 nm) due to the cleavage of 5'-6-FAM-dArUdAdA-6-TAMRA-3' substrate by wild-type SARS-CoV-2 Nsp15. Reactions were allowed to proceed for 30 min at 25°C in the presence of 150 nM of SARS-CoV-2 Nsp15, 1.2 μM substrate, and 100 μM of each compound. Error bars represent the standard deviation of duplicate samples.

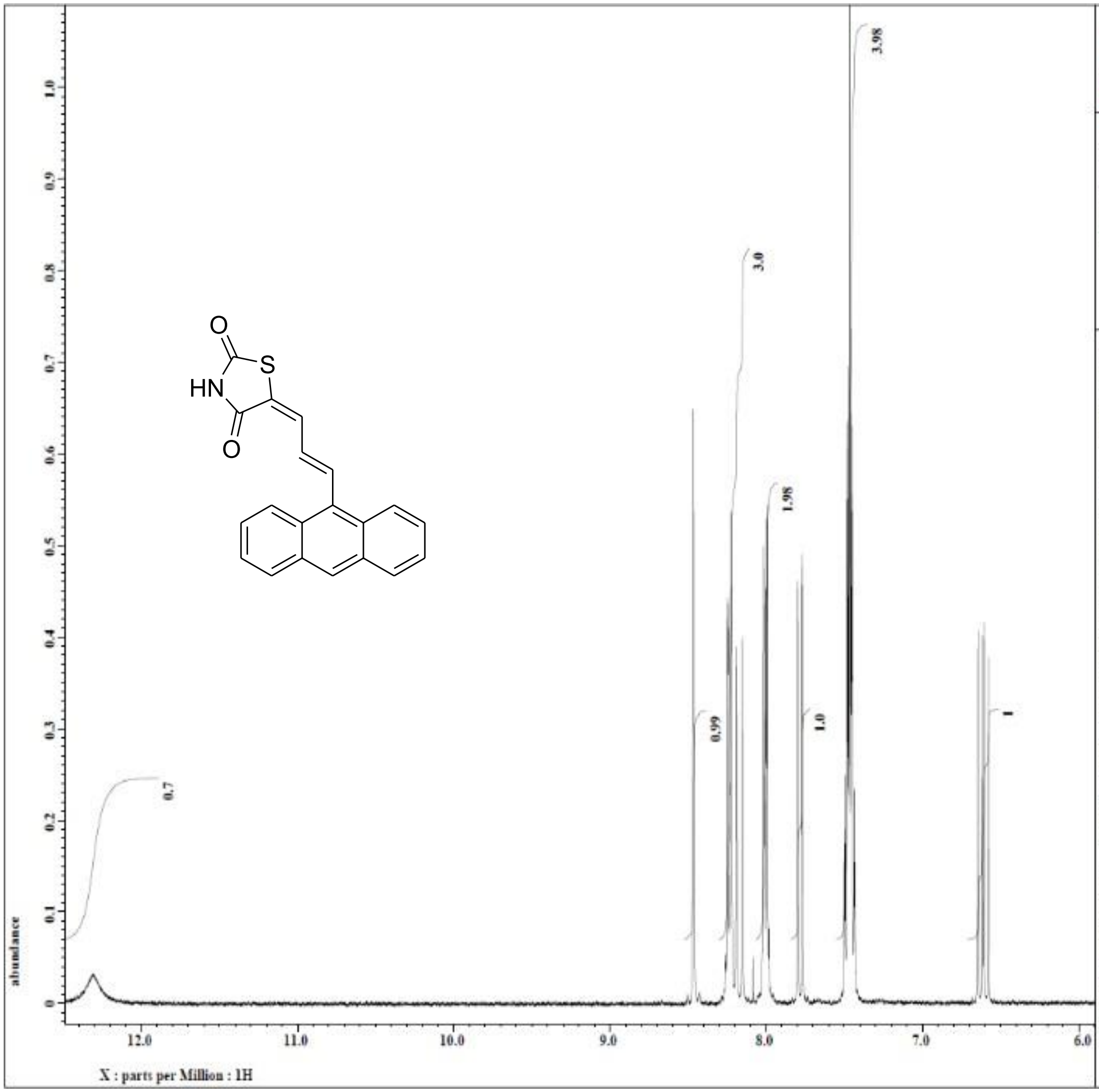

JEOL

---- PROCESSING PARAMETERS ----
dc_balance : 0 : FALSE
sexp : 0.2[Hz] : 0.0[s]
trapezoid3 : 0[%] : 80[%] : 100[%]
zerofill : 1
fft : 1 : TRUE : TRUE
machinephase
ppm

Derived from: Exp-9.104 new batch in 25%

Filename	= Exp-9.104 new batch 1
Author	= datum
Experiment	= single_pulse.ex2
Sample_id	= Exp-9.104 new batch 1
Solvent	= DMSO-D6
Creation_time	= 27-NOV-2022 12:37:11
Revision_time	= 27-NOV-2022 12:33:43
Current_time	= 27-NOV-2022 12:34:01
Comment	= single_pulse
Data_format	= 1D COMPLEX
Dim_size	= 26214
Dim_title	= 1H
Dim_units	= [ppm]
Dimensions	= X
Site	= ECS 400
Spectrometer	= JNM-ECS400
Field_strength	= 9.389766[T] (400[MHz]
X_acq_duration	= 4.36731904[s]
X_domain	= 1H
X_freq	= 399.78219838[MHz]
X_offset	= 5[ppm]
X_points	= 32768
X_prescans	= 1
X_resolution	= 0.22897343[Hz]
X_sweep	= 7.5030012[kHz]
Irr_domain	= 1H
Irr_freq	= 399.78219838[MHz]
Irr_offset	= 5[ppm]
Tri_domain	= 1H
Tri_freq	= 399.78219838[MHz]
Tri_offset	= 5[ppm]
Clipped	= FALSE
Mod_return	= 1
Scans	= 10
Total_scans	= 10
X_90_width	= 9.86[us]
X_acq_time	= 4.36731904[s]
X_angle	= 45[deg]
X_atn	= 0.2[dB]
X_pulse	= 4.93[us]
Irr_mode	= Off
Tri_mode	= Off
Dante_presat	= FALSE
Initial_wait	= 1[s]
Recvr_gain	= 52
Relaxation_delay	= 5[s]
Repetition_time	= 9.36731904[s]
Temp_get	= 21.3[dC]

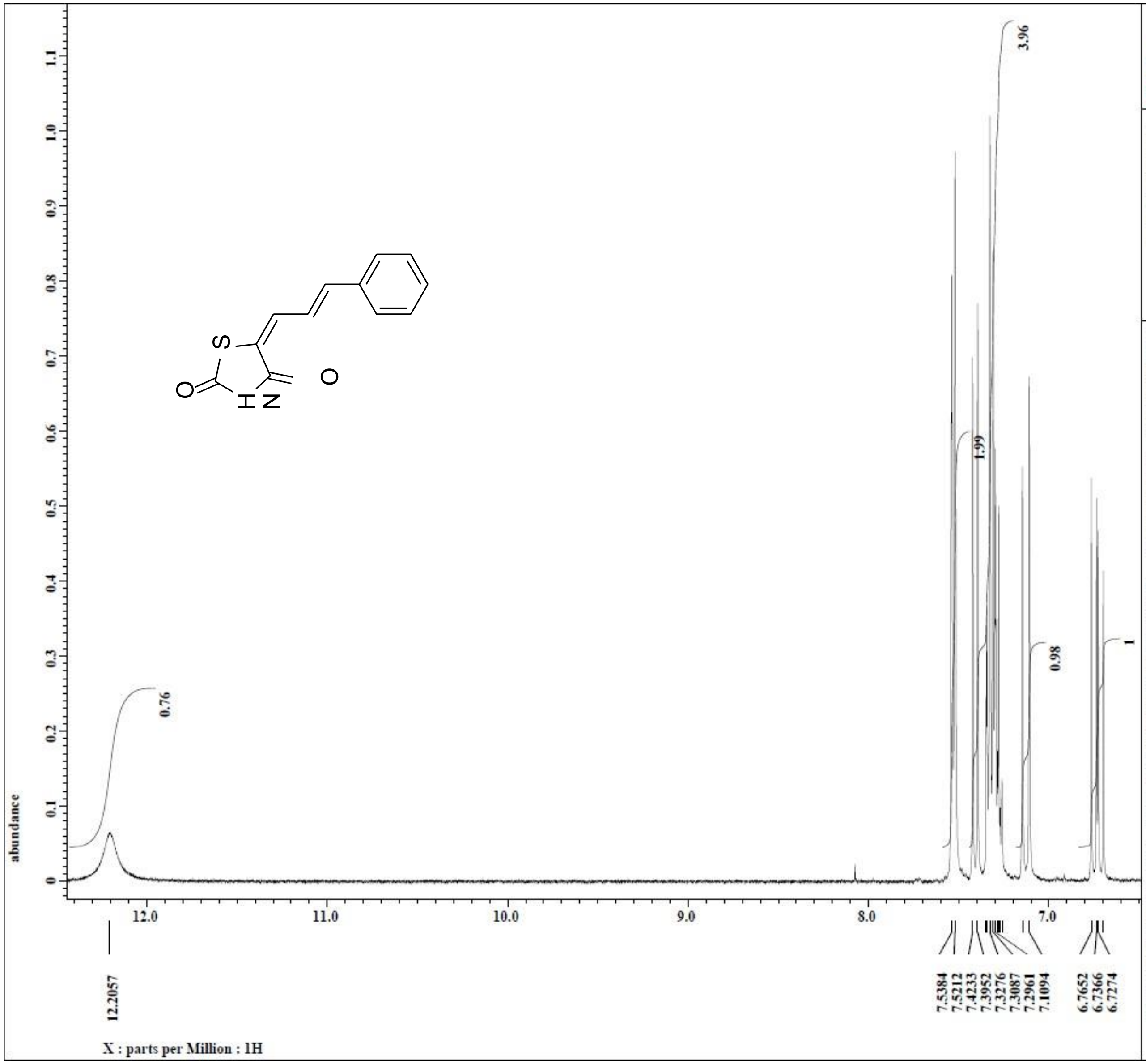

JEOL

```
---- PROCESSING PARAMETERS ----
dc_balance : 0 : FALSE
sexp : 0.2[Hz] : 0.0[s]
trapezoid3 : 0[%] : 80[%] : 100[%]
zerofill : 1
fft : 1 : TRUE : TRUE
machinephase
ppm

Derived from: Exp-9.105 proton in 33% DM
```

```
Filename          = Exp-9.105 proton in 3
Author            = datum
Experiment        = single_pulse.ex2
Sample_id         = Exp-9.105 proton in 3
Solvent           = DMSO-D6
Creation_time     = 15-MAY-2022 16:16:01
Revision_time     = 15-MAY-2022 16:12:05
Current_time      = 15-MAY-2022 16:12:22

Comment           = single_pulse
Data_format       = 1D COMPLEX
Dim_size          = 26214
Dim_title         = 1H
Dim_units         = [ppm]
Dimensions        = X
Site              = ECS 400
Spectrometer      = JNM-ECS400

Field_strength    = 9.389766[T] (400[MHz]
X_acq_duration    = 4.36731904[s]
X_domain          = 1H
X_freq            = 399.78219838[MHz]
X_offset          = 5[ppm]
X_points          = 32768
X_prescans        = 1
X_resolution      = 0.22897343[Hz]
X_sweep           = 7.5030012[kHz]
Irr_domain        = 1H
Irr_freq          = 399.78219838[MHz]
Irr_offset        = 5[ppm]
Tri_domain        = 1H
Tri_freq          = 399.78219838[MHz]
Tri_offset        = 5[ppm]
Clipped           = FALSE
Mod_return        = 1
Scans             = 8
Total_scans       = 8

X_90_width        = 9.86[us]
X_acq_time        = 4.36731904[s]
X_angle           = 45[deg]
X_atn             = 0.2[dB]
X_pulse           = 4.93[us]
Irr_mode          = Off
Tri_mode          = Off
Dante_presat      = FALSE
Initial_wait      = 1[s]
Recvr_gain        = 50
Relaxation_delay  = 5[s]
Repetition_time   = 9.36731904[s]
Temp_get          = 22.5[dC]
```

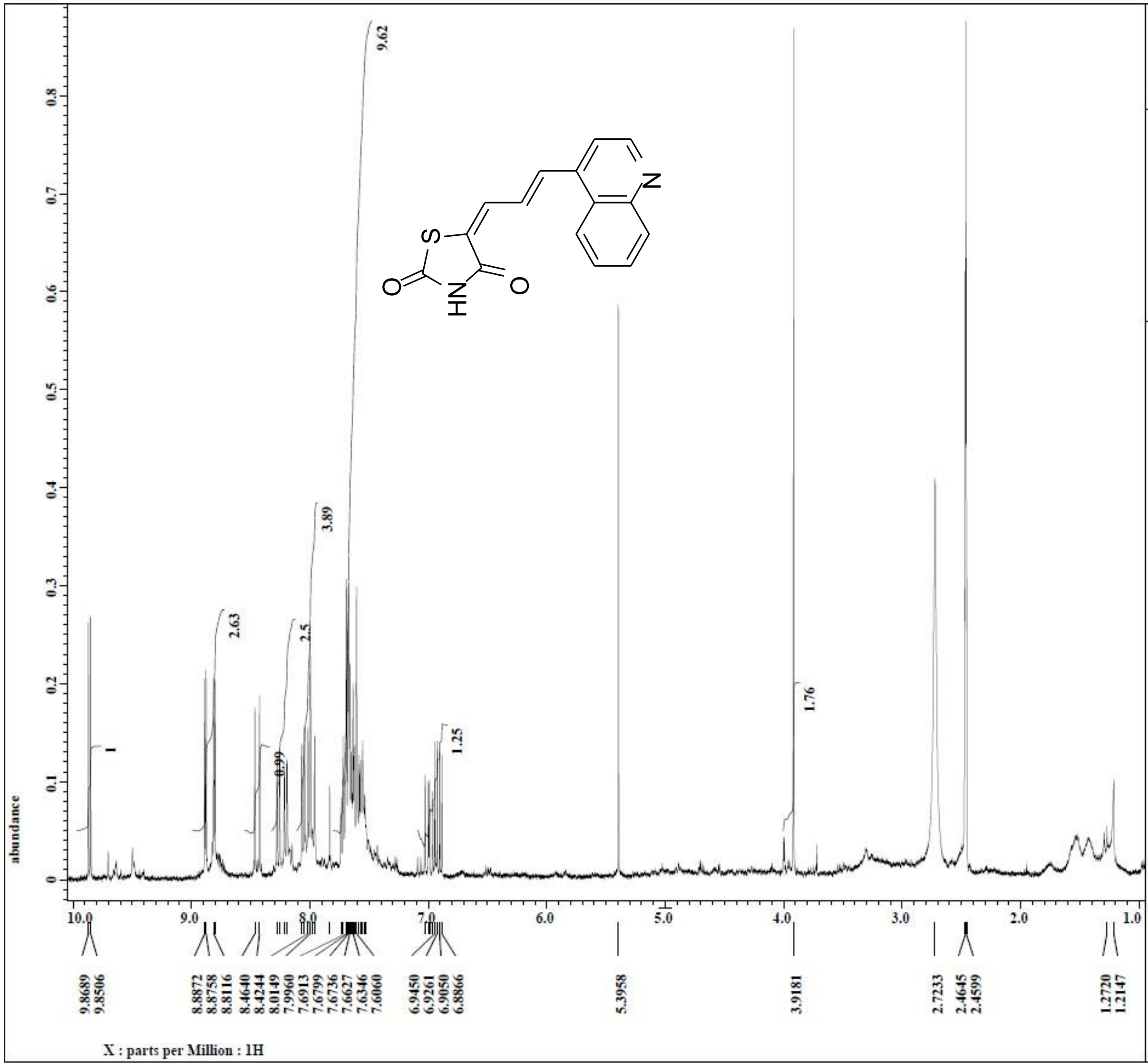


```
---- PROCESSING PARAMETERS ----
dc_balance : 0 : FALSE
sexp : 0.2[Hz] : 0.0[s]
trapezoid3 : 0[%] : 80[%] : 100[%]
zerofill : 1
fft : 1 : TRUE : TRUE
machinephase
ppm

Derived from: EXP 9.121 KCO-48 DMSO CCL4

Filename           = EXP 9.121 KCO-48 DMSO
Author             = datum
Experiment         = single_pulse.ex2
Sample_id          = EXP 9.121 KCO-48 DMSO
Solvent            = DMSO-D6
Creation_time      = 24-DEC-2019 14:35:40
Revision_time      = 24-DEC-2019 14:17:51
Current_time       = 24-DEC-2019 14:18:22

Comment            = single_pulse
Data_format        = 1D COMPLEX
Dim_size           = 26214
Dim_title          = 1H
Dim_units          = [ppm]
Dimensions         = X
Site               = ECS 400
Spectrometer       = JNM-ECS400

Field_strength     = 9.389766[T] (400[MHz]
X_acq_duration     = 4.36731904[s]
X_domain           = 1H
X_freq             = 399.78219838[MHz]
X_offset           = 5[ppm]
X_points           = 32768
X_prescans         = 1
X_resolution       = 0.22897343[Hz]
X_sweep            = 7.5030012[kHz]
Irr_domain         = 1H
Irr_freq           = 399.78219838[MHz]
Irr_offset         = 5[ppm]
Tri_domain         = 1H
Tri_freq           = 399.78219838[MHz]
Tri_offset         = 5[ppm]
Clipped            = FALSE
Mod_return         = 1
Scans              = 16
Total_scans        = 16

X_90_width         = 9.86[us]
X_acq_time         = 4.36731904[s]
X_angle            = 45[deg]
X_atn              = 0.2[dB]
X_pulse            = 4.93[us]
Irr_mode           = Off
Tri_mode           = Off
Dante_presat       = FALSE
Initial_wait       = 1[s]
Recvr_gain         = 56
Relaxation_delay   = 5[s]
Repetition_time    = 9.36731904[s]
Temp_get           = 20.4[dC]
```

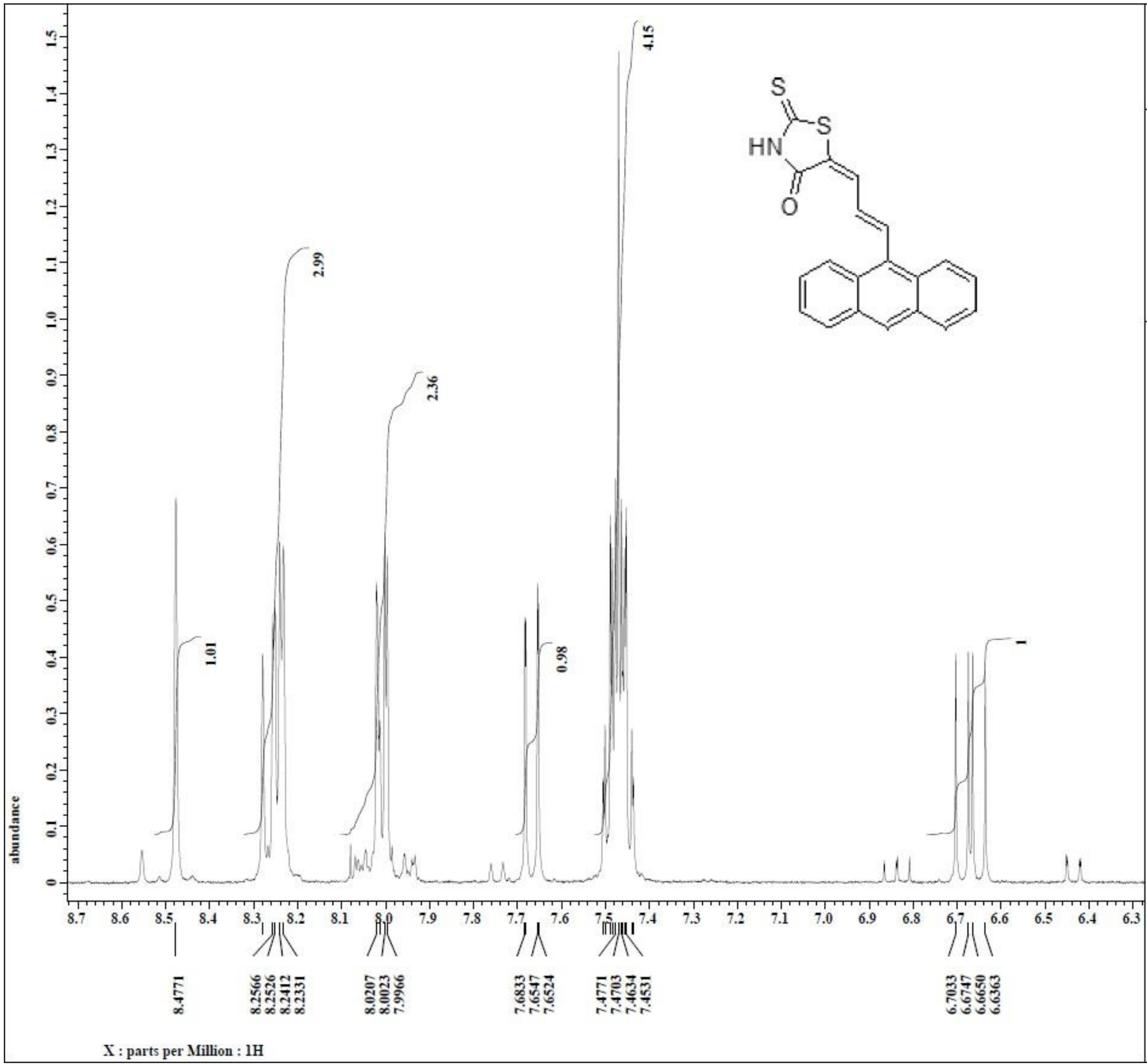

---- PROCESSING PARAMETERS ----
dc_balance : 0 : FALSE
sexp : 0.2[Hz] : 0.0[s]
trapezoid3 : 0[%] : 80[%] : 100[%]
zerofill : 1
fft : 1 : TRUE : TRUE
machinephase
ppm

Derived from: Exp-9.127 proton in 25% DM

Filename	= Exp-9.127 proton in 2
Author	= datum
Experiment	= single_pulse.ex2
Sample_id	= Exp-9.127 proton in 2
Solvent	= DMSO-D6
Creation_time	= 25-NOV-2022 12:07:13
Revision_time	= 25-NOV-2022 12:05:45
Current_time	= 25-NOV-2022 12:06:01
Comment	= single_pulse
Data_format	= 1D COMPLEX
Dim_size	= 26214
Dim_title	= 1H
Dim_units	= [ppm]
Dimensions	= X
Site	= ECS 400
Spectrometer	= JNM-ECS400
Field_strength	= 9.389766[T] (400[MHz]
X_acq_duration	= 4.36731904[s]
X_domain	= 1H
X_freq	= 399.78219838[MHz]
X_offset	= 5[ppm]
X_points	= 32768
X_prescans	= 1
X_resolution	= 0.22897343[Hz]
X_sweep	= 7.5030012[kHz]
Irr_domain	= 1H
Irr_freq	= 399.78219838[MHz]
Irr_offset	= 5[ppm]
Tri_domain	= 1H
Tri_freq	= 399.78219838[MHz]
Tri_offset	= 5[ppm]
Clipped	= FALSE
Mod_return	= 1
Scans	= 8
Total_scans	= 8
X_90_width	= 9.86[us]
X_acq_time	= 4.36731904[s]
X_angle	= 45[deg]
X_atn	= 0.2[dB]
X_pulse	= 4.93[us]
Irr_mode	= Off
Tri_mode	= Off
Dante_presat	= FALSE
Initial_wait	= 1[s]
Recvr_gain	= 50
Relaxation_delay	= 5[s]
Repetition_time	= 9.36731904[s]
Temp_get	= 20.8[dC]

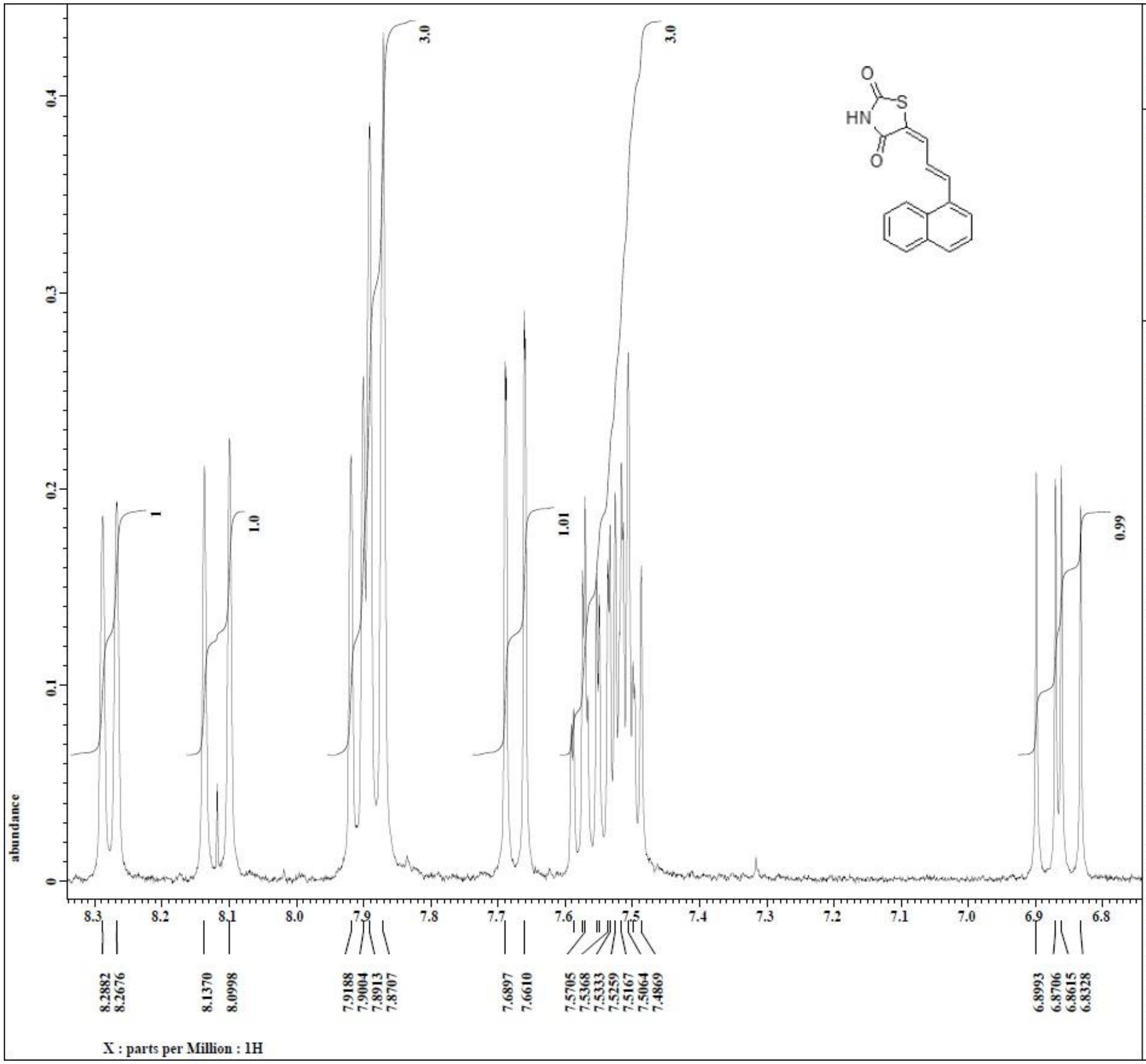


```
---- PROCESSING PARAMETERS ----
dc_balance : 0 : FALSE
sexp : 0.2[Hz] : 0.0[s]
trapezoid3 : 0[%] : 80[%] : 100[%]
zerofill : 1
fft : 1 : TRUE : TRUE
machinephase
ppm
reference : -0.035[ppm] : 0

Derived from: Exp-16.14 proton in 33% DM

Filename          = Exp-16.14 proton in 3
Author            = datum
Experiment        = single_pulse.ex2
Sample_id         = Exp-16.14 proton in 3
Solvent           = DMSO-D6
Creation_time     = 22-FEB-2022 12:09:43
Revision_time     = 22-FEB-2022 12:11:43
Current_time      = 22-FEB-2022 12:12:06

Comment           = single_pulse
Data_format       = 1D COMPLEX
Dim_size          = 26214
Dim_title         = 1H
Dim_units         = [ppm]
Dimensions        = X
Site              = ECS 400
Spectrometer      = JNM-ECS400

Field_strength    = 9.389766[T] (400[MHz]
X_acq_duration    = 4.36731904[s]
X_domain          = 1H
X_freq            = 399.78219838[MHz]
X_offset          = 5[ppm]
X_points          = 32768
X_prescans        = 1
X_resolution      = 0.22897343[Hz]
X_sweep           = 7.5030012[kHz]
Irr_domain        = 1H
Irr_freq          = 399.78219838[MHz]
Irr_offset        = 5[ppm]
Tri_domain        = 1H
Tri_freq          = 399.78219838[MHz]
Tri_offset        = 5[ppm]
Clipped           = FALSE
Mod_return        = 1
Scans             = 19
Total_scans       = 19

X_90_width        = 9.86[us]
X_acq_time        = 4.36731904[s]
X_angle           = 45[deg]
X_atn             = 0.2[dB]
X_pulse           = 4.93[us]
Irr_mode          = Off
Tri_mode          = Off
Dante_presat      = FALSE
Initial_wait      = 1[s]
Recvr_gain        = 52
Relaxation_delay  = 5[s]
Repetition_time   = 9.36731904[s]
Temp_get          = 21.3[dC]
```

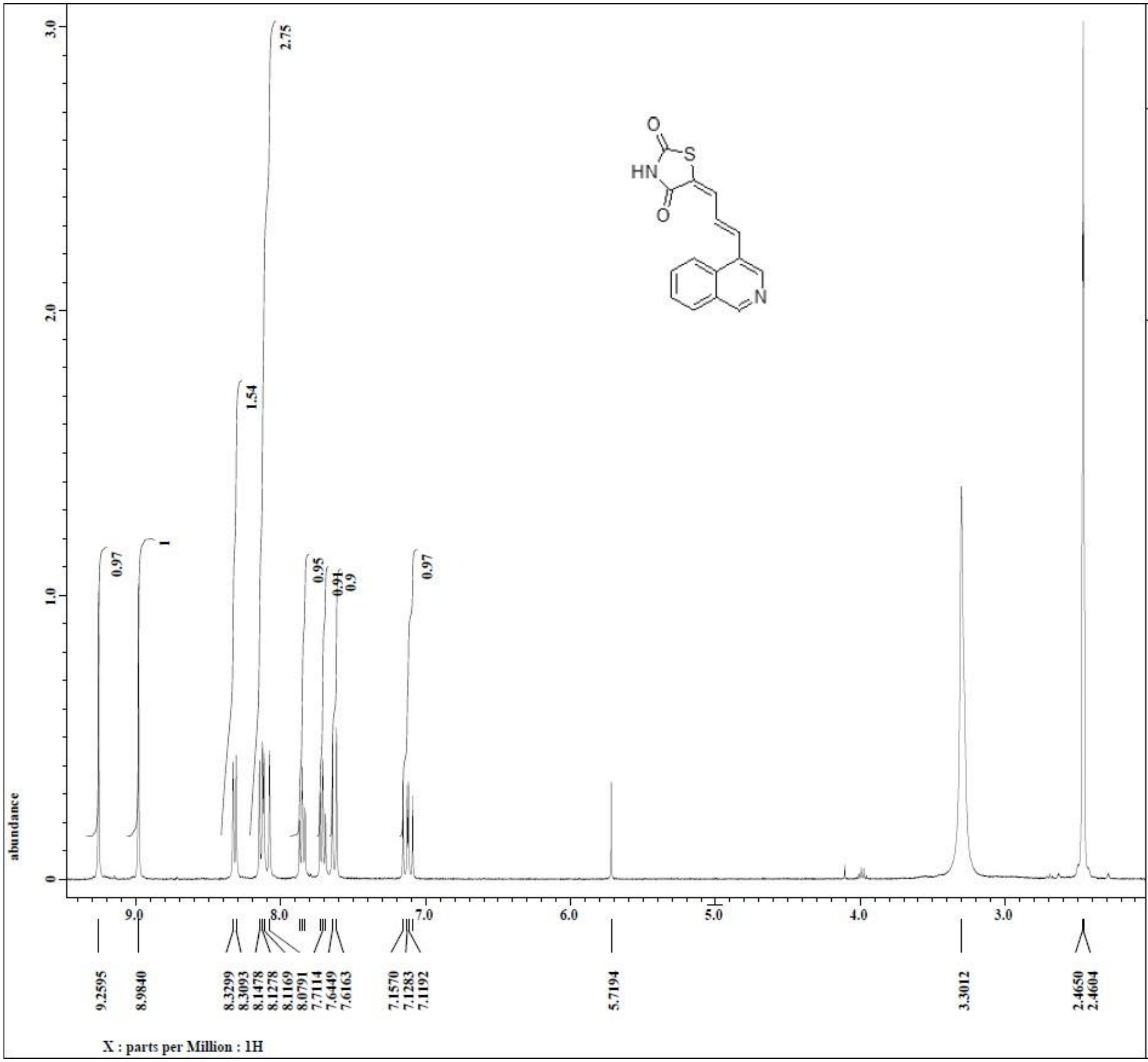

---- PROCESSING PARAMETERS ----
dc_balance : 0 : FALSE
sexp : 0.2[Hz] : 0.0[s]
trapezoid3 : 0[%] : 80[%] : 100[%]
zerofill : 1
fft : 1 : TRUE : TRUE
machinephase
ppm

Derived from: II3-16 DMSO PROTON-1.jdf

```
Filename            = II3-16 DMSO PROTON-3.
Author              = datum
Experiment          = single_pulse.ex2
Sample_id           = II3-16 DMSO PROTON
Solvent             = DMSO-D6
Creation_time       = 28-FEB-2022 12:25:01
Revision_time       = 28-FEB-2022 12:25:02
Current_time        = 28-FEB-2022 12:25:22

Comment             = single_pulse
Data_format         = 1D COMPLEX
Dim_size            = 26214
Dim_title           = 1H
Dim_units           = [ppm]
Dimensions          = X
Site                = ECS 400
Spectrometer        = JNM-ECS400

Field_strength      = 9.389766[T] (400[MHz]
X_acq_duration      = 4.36731904[s]
X_domain            = 1H
X_freq              = 399.78219838[MHz]
X_offset            = 5[ppm]
X_points            = 32768
X_prescans          = 1
X_resolution        = 0.22897343[Hz]
X_sweep             = 7.5030012[kHz]
Irr_domain          = 1H
Irr_freq            = 399.78219838[MHz]
Irr_offset          = 5[ppm]
Tri_domain          = 1H
Tri_freq            = 399.78219838[MHz]
Tri_offset          = 5[ppm]
Clipped             = FALSE
Mod_return          = 1
Scans               = 16
Total_scans         = 16

X_90_width          = 9.86[us]
X_acq_time          = 4.36731904[s]
X_angle             = 45[deg]
X_atn               = 0.2[dB]
X_pulse             = 4.93[us]
Irr_mode            = Off
Tri_mode            = Off
Dante_presat        = FALSE
Initial_wait        = 1[s]
Recvr_gain          = 52
Relaxation_delay    = 5[s]
Repetition_time     = 9.36731904[s]
Temp_get            = 20.1[dC]
```

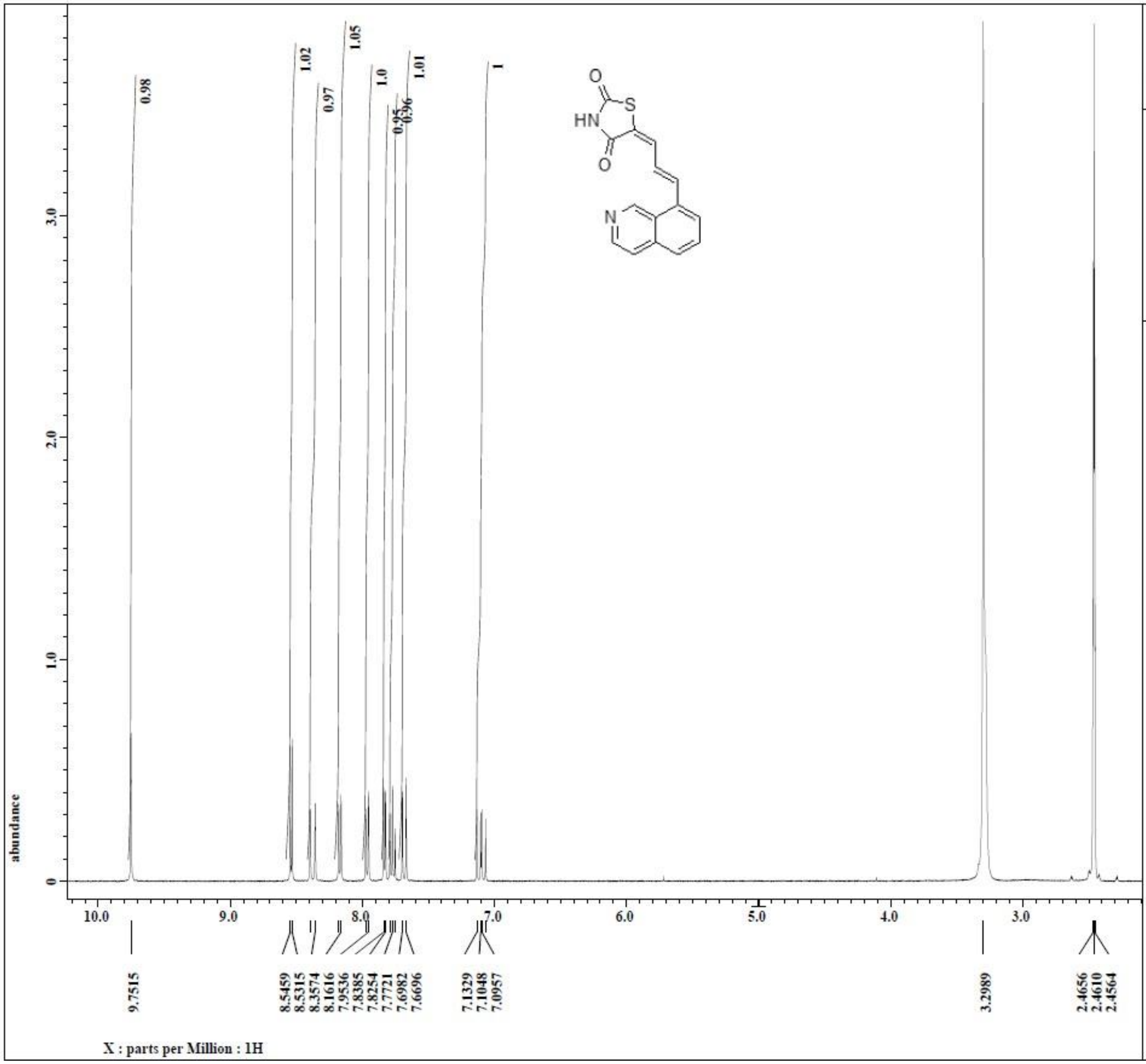


```
---- PROCESSING PARAMETERS ----
dc_balance : 0 : FALSE
sexp : 0.2[Hz] : 0.0[s]
trapezoid3 : 0[%] : 80[%] : 100[%]
zerofill : 1
fft : 1 : TRUE : TRUE
machinephase
ppm

Derived from: SH5-56 S DMSO-1.jdf

Filename          = SH5-56 S DMSO-3.jdf
Author            = datum
Experiment        = single_pulse.ex2
Sample_id         = SH5-56 S DMSO
Solvent           = DMSO-D6
Creation_time     = 28-FEB-2022 14:12:08
Revision_time     = 28-FEB-2022 14:11:56
Current_time      = 28-FEB-2022 14:12:42

Comment           = single_pulse
Data_format       = 1D COMPLEX
Dim_size          = 26214
Dim_title         = 1H
Dim_units         = [ppm]
Dimensions        = X
Site              = ECS 400
Spectrometer      = JNM-ECS400

Field_strength    = 9.389766[T] (400[MHz]
X_acq_duration    = 4.36731904[s]
X_domain          = 1H
X_freq            = 399.78219838[MHz]
X_offset          = 5[ppm]
X_points          = 32768
X_prescans        = 1
X_resolution      = 0.22897343[Hz]
X_sweep           = 7.5030012[kHz]
Irr_domain        = 1H
Irr_freq          = 399.78219838[MHz]
Irr_offset        = 5[ppm]
Tri_domain        = 1H
Tri_freq          = 399.78219838[MHz]
Tri_offset        = 5[ppm]
Clipped           = FALSE
Mod_return        = 1
Scans             = 16
Total_scans       = 16

X_90_width        = 9.86[us]
X_acq_time        = 4.36731904[s]
X_angle           = 45[deg]
X_atn             = 0.2[dB]
X_pulse           = 4.93[us]
Irr_mode          = Off
Tri_mode          = Off
Dante_presat      = FALSE
Initial_wait      = 1[s]
Recvr_gain        = 52
Relaxation_delay  = 5[s]
Repetition_time   = 9.36731904[s]
Temp_get          = 20.6[dC]
```

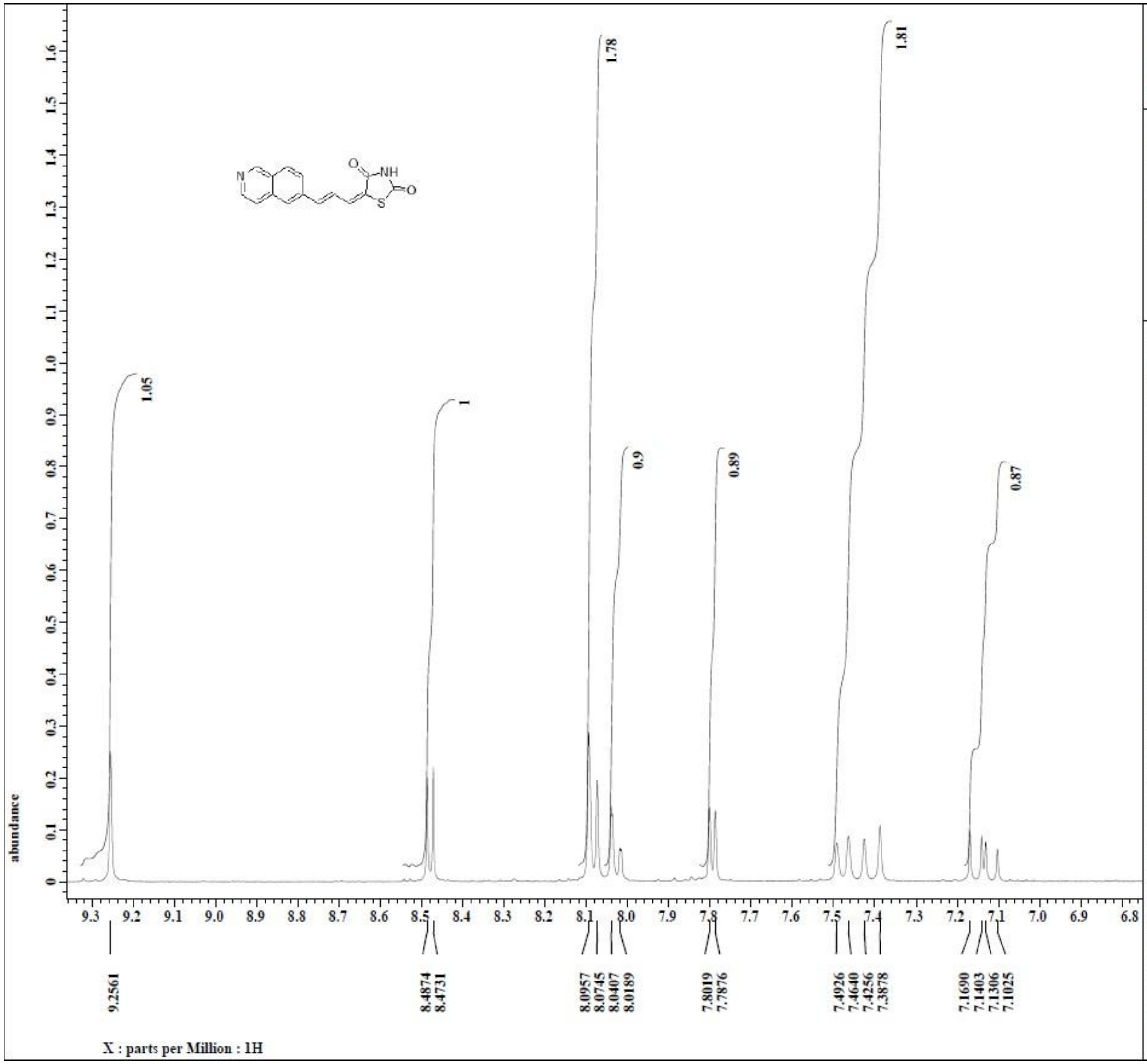


```
---- PROCESSING PARAMETERS ----
dc_balance : 0 : FALSE
sexp : 0.2[Hz] : 0.0[s]
trapezoid3 : 0[%] : 80[%] : 100[%]
zerofill : 1
fft : 1 : TRUE : TRUE
machinephase
ppm

Derived from: SH5-57 DMSO PROTON-1.jdf

Filename          = SH5-57 DMSO PROTON-3.
Author            = datum
Experiment        = single_pulse.ex2
Sample_id         = SH5-57 DMSO PROTON
Solvent           = DMSO-D6
Creation_time     =  1-MAR-2022 16:37:29
Revision_time     =  1-MAR-2022 16:37:50
Current_time      =  1-MAR-2022 16:40:19

Comment           = single_pulse
Data_format       = 1D COMPLEX
Dim_size          = 26214
Dim_title         = 1H
Dim_units         = [ppm]
Dimensions        = X
Site              = ECS 400
Spectrometer      = JNM-ECS400

Field_strength    = 9.389766[T] (400[MHz]
X_acq_duration    = 4.36731904[s]
X_domain          = 1H
X_freq            = 399.78219838[MHz]
X_offset          = 5[ppm]
X_points          = 32768
X_prescans        = 1
X_resolution      = 0.22897343[Hz]
X_sweep           = 7.5030012[kHz]
Irr_domain        = 1H
Irr_freq          = 399.78219838[MHz]
Irr_offset        = 5[ppm]
Tri_domain        = 1H
Tri_freq          = 399.78219838[MHz]
Tri_offset        = 5[ppm]
Clipped           = FALSE
Mod_return        = 1
Scans             = 270
Total_scans       = 270

X_90_width        = 9.86[us]
X_acq_time        = 4.36731904[s]
X_angle           = 45[deg]
X_atn             = 0.2[dB]
X_pulse           = 4.93[us]
Irr_mode          = Off
Tri_mode          = Off
Dante_presat      = FALSE
Initial_wait      = 1[s]
Recvr_gain        = 54
Relaxation_delay  = 5[s]
Repetition_time   = 9.36731904[s]
Temp_get          = 20.4[dC]
```

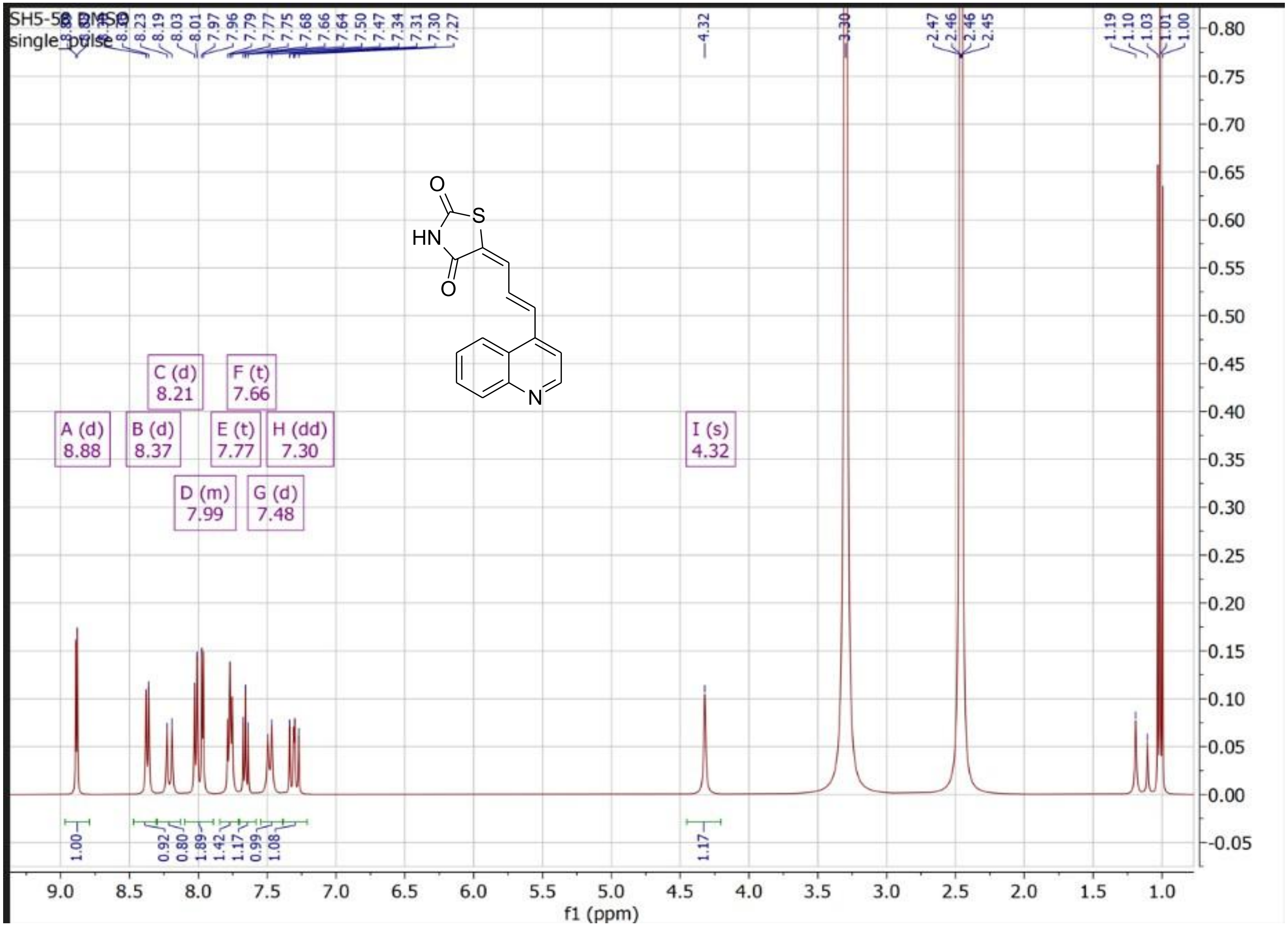

SH5-58 DMSO
single_pulse
A (d) 8.88
B (d) 8.37
C (d) 8.21
D (m) 7.99
E (t) 7.77
F (t) 7.66
G (d) 7.48
H (dd) 7.30
I (s) 4.32
HN
S
O
O
N
f1 (ppm)

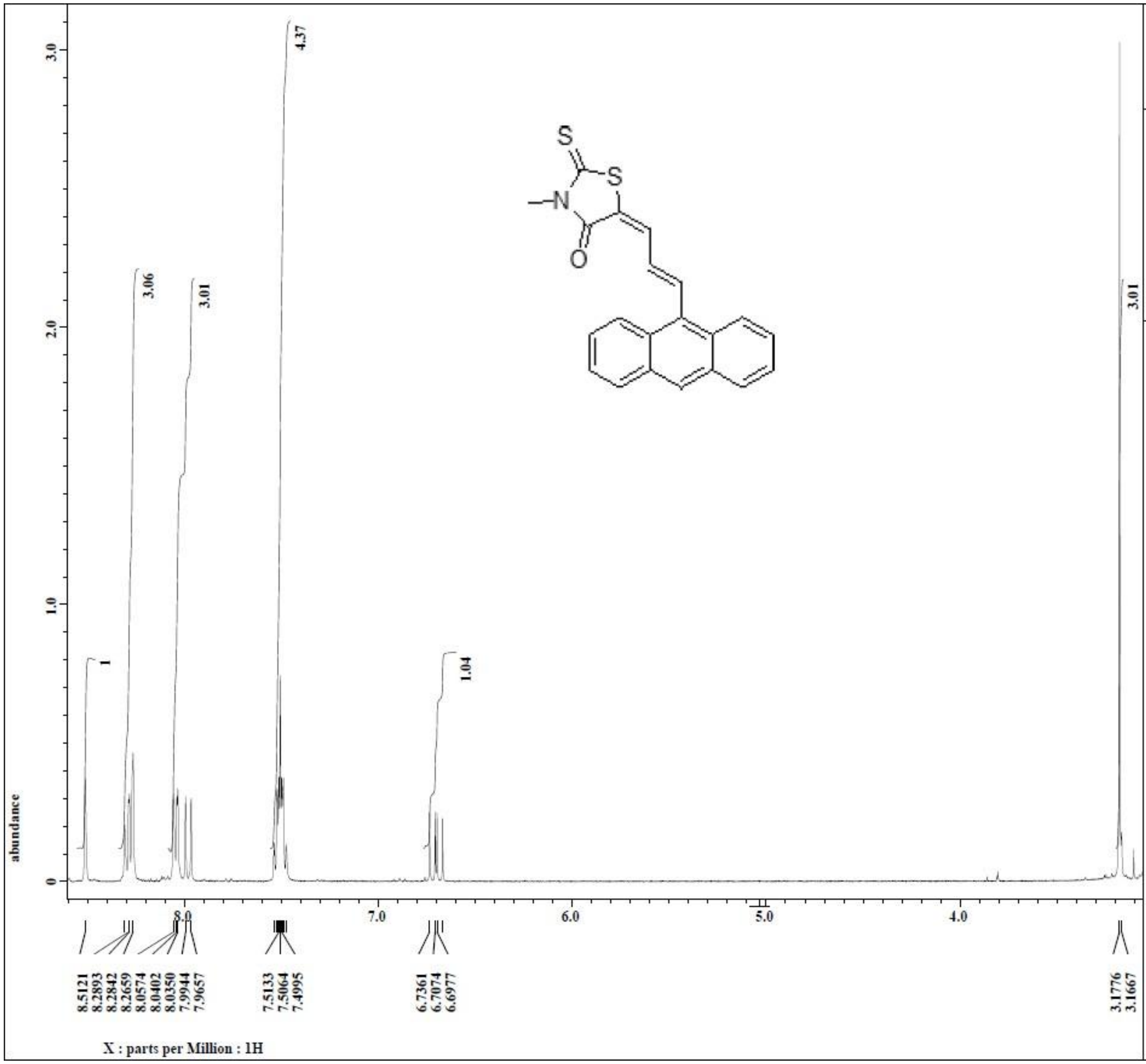


```
---- PROCESSING PARAMETERS ----
dc_balance : 0 : FALSE
sexp : 0.2[Hz] : 0.0[s]
trapezoid3 : 0[%] : 80[%] : 100[%]
zerofill : 1
fft : 1 : TRUE : TRUE
machinephase
ppm
reference : -0.035[ppm] : 0

Derived from: Exp-16.20 proton in 33% DM
```

```
Filename          = Exp-16.20 proton in 3
Author            = datum
Experiment        = single_pulse.ex2
Sample_id         = Exp-16.20 proton in 3
Solvent           = DMSO-D6
Creation_time     =  3-MAR-2022 09:14:58
Revision_time     =  3-MAR-2022 09:20:17
Current_time      =  3-MAR-2022 09:20:38

Comment           = single_pulse
Data_format       = 1D COMPLEX
Dim_size          = 26214
Dim_title         = 1H
Dim_units         = [ppm]
Dimensions        = X
Site              = ECS 400
Spectrometer      = JNM-ECS400

Field_strength    = 9.389766[T] (400[MHz]
X_acq_duration    = 4.36731904[s]
X_domain          = 1H
X_freq            = 399.78219838[MHz]
X_offset          = 5[ppm]
X_points          = 32768
X_prescans        = 1
X_resolution      = 0.22897343[Hz]
X_sweep           = 7.5030012[kHz]
Irr_domain        = 1H
Irr_freq          = 399.78219838[MHz]
Irr_offset        = 5[ppm]
Tri_domain        = 1H
Tri_freq          = 399.78219838[MHz]
Tri_offset        = 5[ppm]
Clipped           = FALSE
Mod_return        = 1
Scans             = 26
Total_scans       = 26

X_90_width        = 9.86[us]
X_acq_time        = 4.36731904[s]
X_angle           = 45[deg]
X_atn             = 0.2[dB]
X_pulse           = 4.93[us]
Irr_mode          = Off
Tri_mode          = Off
Dante_presat      = FALSE
Initial_wait      = 1[s]
Recvr_gain        = 54
Relaxation_delay  = 5[s]
Repetition_time   = 9.36731904[s]
Temp_get          = 20.4[dC]
```

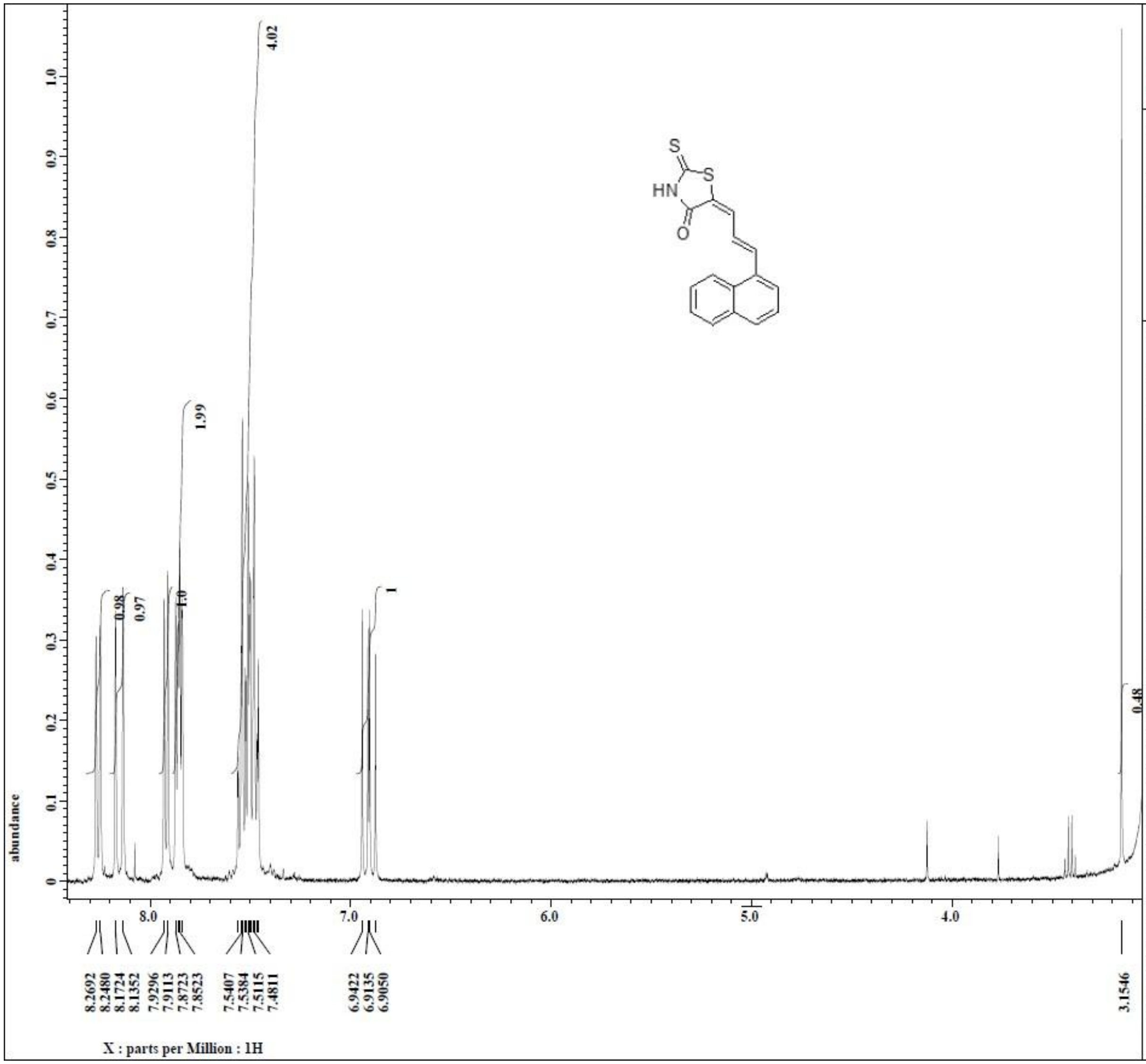

JEOL

---- PROCESSING PARAMETERS ----
dc_balance : 0 : FALSE
sexp : 0.2[Hz] : 0.0[s]
trapezoid3 : 0[%] : 80[%] : 100[%]
zerofill : 1
fft : 1 : TRUE : TRUE
machinephase
ppm

Derived from: Exp-16.31 proton 33% DMSO-

Filename	= Exp-16.31 proton 33%
Author	= datum
Experiment	= single_pulse.ex2
Sample_id	= Exp-16.31 proton 33%
Solvent	= DMSO-D6
Creation_time	= 6-APR-2022 14:58:11
Revision_time	= 6-APR-2022 14:56:26
Current_time	= 6-APR-2022 14:56:43
Comment	= single_pulse
Data_format	= 1D COMPLEX
Dim_size	= 26214
Dim_title	= 1H
Dim_units	= [ppm]
Dimensions	= X
Site	= ECS 400
Spectrometer	= JNM-ECS400
Field_strength	= 9.389766[T] (400[MHz]
X_acq_duration	= 4.36731904[s]
X_domain	= 1H
X_freq	= 399.78219838[MHz]
X_offset	= 5[ppm]
X_points	= 32768
X_prescans	= 1
X_resolution	= 0.22897343[Hz]
X_sweep	= 7.5030012[kHz]
Irr_domain	= 1H
Irr_freq	= 399.78219838[MHz]
Irr_offset	= 5[ppm]
Tri_domain	= 1H
Tri_freq	= 399.78219838[MHz]
Tri_offset	= 5[ppm]
Clipped	= FALSE
Mod_return	= 1
Scans	= 13
Total_scans	= 13
X_90_width	= 9.86[us]
X_acq_time	= 4.36731904[s]
X_angle	= 45[deg]
X_atn	= 0.2[dB]
X_pulse	= 4.93[us]
Irr_mode	= Off
Tri_mode	= Off
Dante_presat	= FALSE
Initial_wait	= 1[s]
Recvr_gain	= 54
Relaxation_delay	= 5[s]
Repetition_time	= 9.36731904[s]
Temp_get	= 22.7[dC]

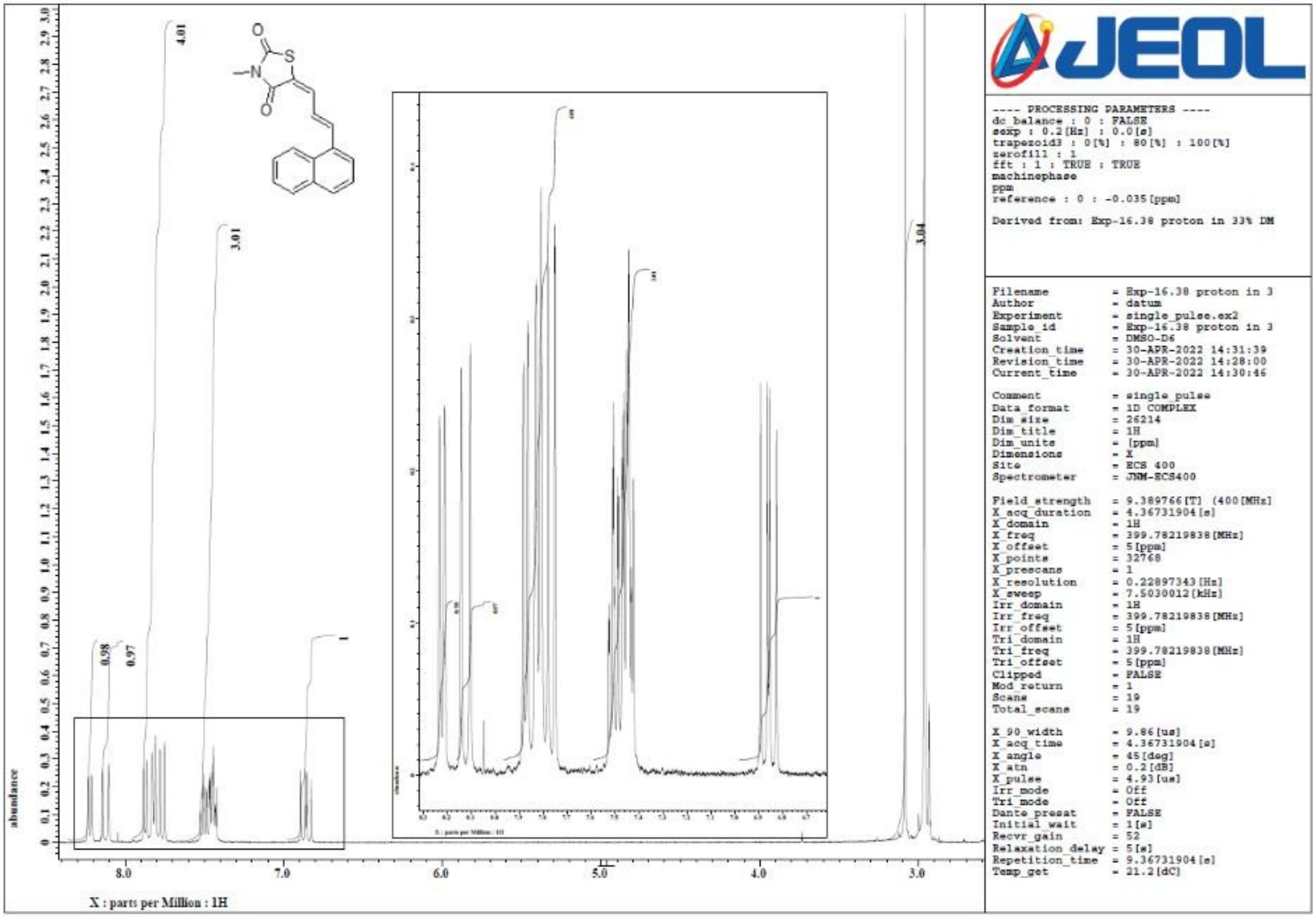
abundance
X : parts per Million : 1H
0.98
0.97
4.01
3.01
1
3.04
JEOL
---- PROCESSING PARAMETERS ----
dc_balance : 0 : FALSE
sexp : 0.2[Hz] : 0.0[s]
trapezoid3 : 0[%] : 80[%] : 100[%]
zerofill : 1
fft : 1 : TRUE : TRUE
machinephase
ppm
reference : 0 : -0.035[ppm]
Derived from: Exp-16.38 proton in 33% DM
Filename = Exp-16.38 proton in 3
Author = datum
Experiment = single_pulse.ex2
Sample_id = Exp-16.38 proton in 3
Solvent = DMSO-D6
Creation_time = 30-APR-2022 14:31:39
Revision_time = 30-APR-2022 14:28:00
Current_time = 30-APR-2022 14:30:46
Comment = single_pulse
Data_format = 1D COMPLEX
Dim_size = 26214
Dim_title = 1H
Dim_units = [ppm]
Dimensions = X
Site = ECS 400
Spectrometer = JNM-ECS400
Field_strength = 9.389766[T] (400[MHz]
X_acq_duration = 4.36731904[s]
X_domain = 1H
X_freq = 399.78219838[MHz]
X_offset = 5[ppm]
X_points = 32768
X_prescans = 1
X_resolution = 0.22897343[Hz]
X_sweep = 7.5030012[kHz]
Irr_domain = 1H
Irr_freq = 399.78219838[MHz]
Irr_offset = 5[ppm]
Tri_domain = 1H
Tri_freq = 399.78219838[MHz]
Tri_offset = 5[ppm]
Clipped = FALSE
Mod_return = 1
Scans = 19
Total_scans = 19
X_90_width = 9.86[us]
X_acq_time = 4.36731904[s]
X_angle = 45[deg]
X_atn = 0.2[dB]
X_pulse = 4.93[us]
Irr_mode = Off
Tri_mode = Off
Dante_presat = FALSE
Initial_wait = 1[s]
Recvr_gain = 52
Relaxation_delay = 5[s]
Repetition_time = 9.36731904[s]
Temp_get = 21.2[dC]

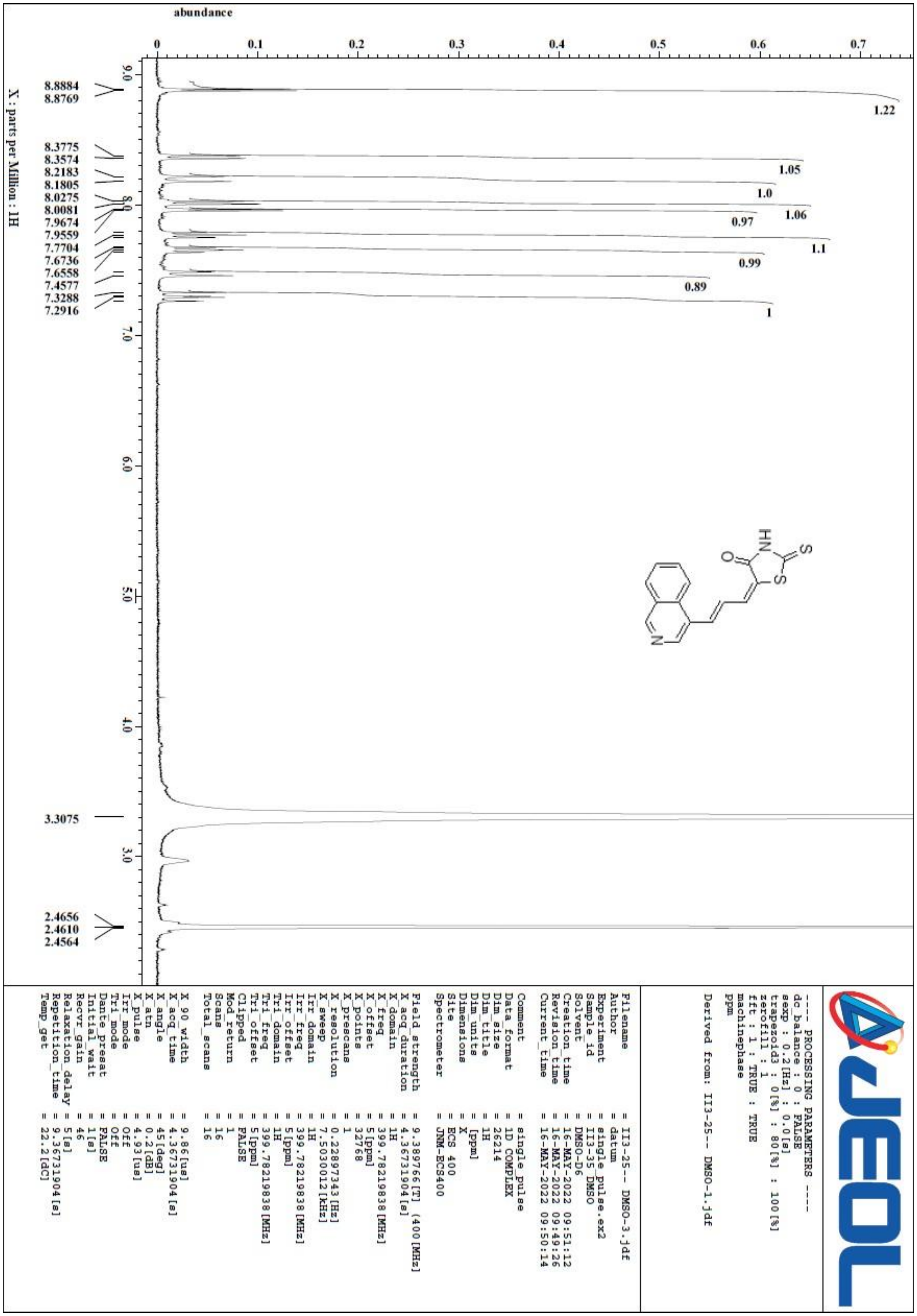

Figure S4. NMR profiles of rhodanine analogs. KCO035, KCO036, KCO230, KCO231, KCO234, KCO235, KCO236, KCO237, KCO238, KCO249, KCO250, KCO251 respectively.

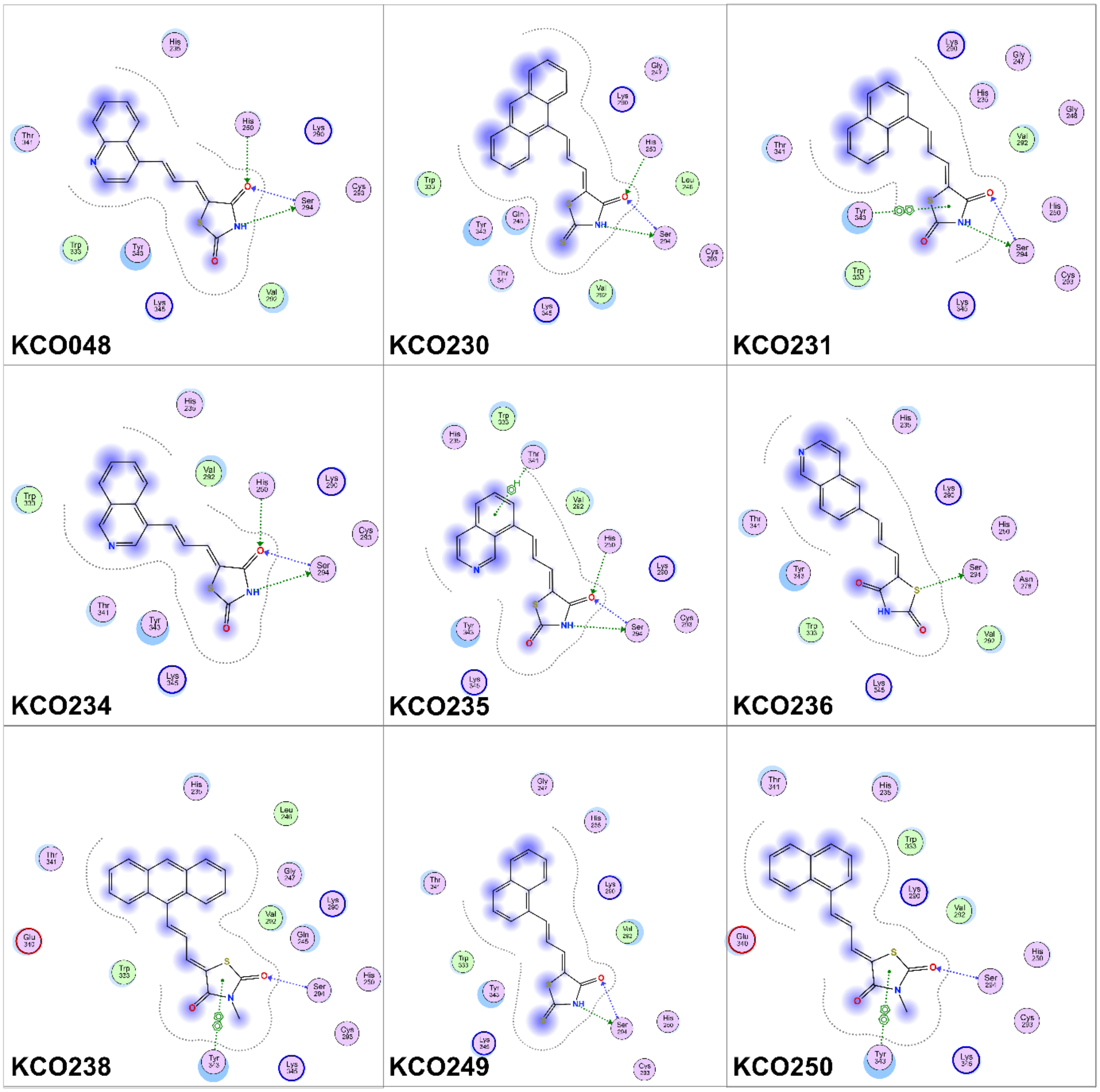

Figure S5. Docking of rhodanine compounds in the SARS-CoV-2 Nsp15 active site. Diagrams showing the interaction maps of rhodanine analogs docked to a rigid SARS-CoV-2 Nsp15 binding site using MOE. The acidic, basic, and polar amino acids are represented by the pink spheres with blue, red, and black outlines, respectively. Hydrophobic residues are presented in green. Blue and green arrows indicate hydrogen bonding to backbone and sidechain atoms, respectively. The fuzzy blue spheres represent the ligand atoms exposed to solvent as determined by the docking process. Light-blue shadows around residues indicate the degree of interaction with ligand atoms. The dotted contour reflects steric. The steric room for methyl substitution is presented by dotted contours.

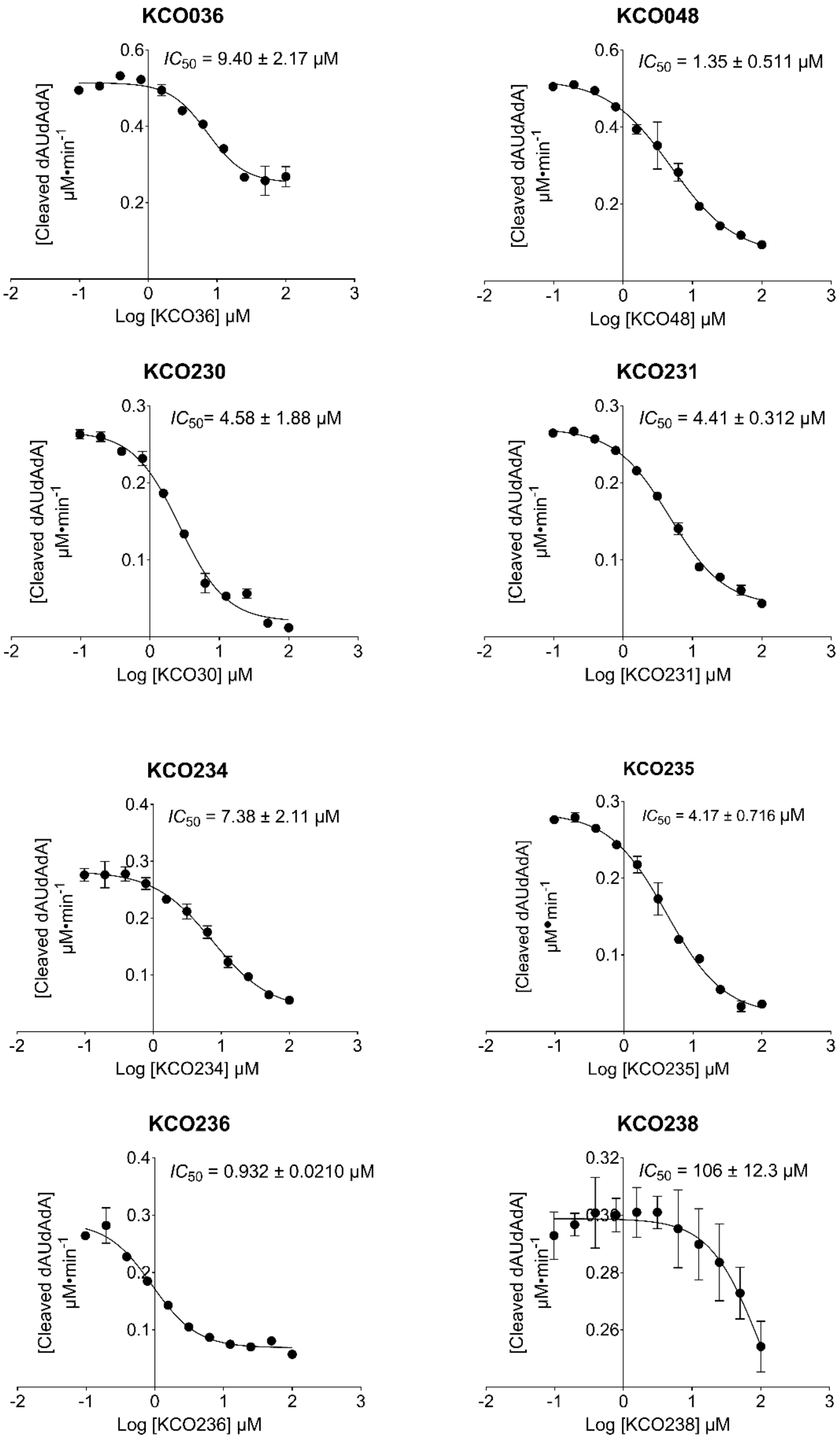
KCO036
IC50 = 9.40 ± 2.17 μM
[Cleaved dAUdAdA] μM•min-1
Log [KCO36] μM
KCO048
IC50 = 1.35 ± 0.511 μM
[Cleaved dAUdAdA] μM•min-1
Log [KCO48] μM
KCO230
IC50= 4.58 ± 1.88 μM
[Cleaved dAUdAdA] μM•min-1
Log [KCO30] μM
KCO231
IC50 = 4.41 ± 0.312 μM
[Cleaved dAUdAdA] μM•min-1
Log [KCO231] μM
KCO234
IC50 = 7.38 ± 2.11 μM
[Cleaved dAUdAdA] μM•min-1
Log [KCO234] μM
KCO235
IC50 = 4.17 ± 0.716 μM
[Cleaved dAUdAdA] μM•min-1
Log [KCO235] μM
KCO236
IC50 = 0.932 ± 0.0210 μM
[Cleaved dAUdAdA] μM•min-1
Log [KCO236] μM
KCO238
IC50 = 106 ± 12.3 μM
[Cleaved dAUdAdA] μM•min-1
Log [KCO238] μM

Fig.S6 cont.

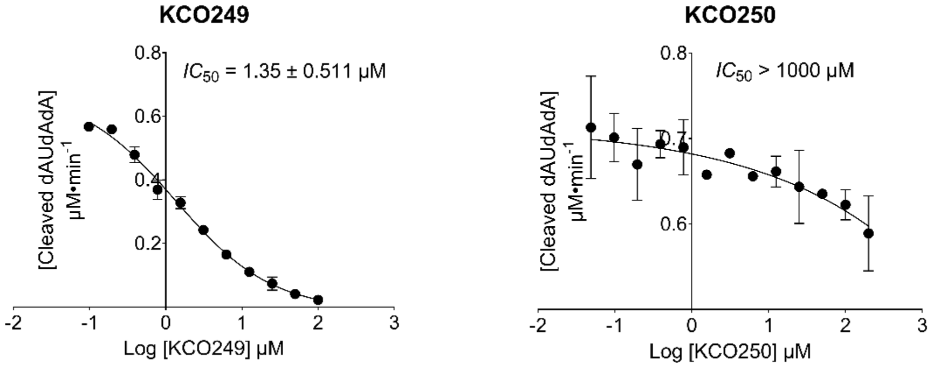

Figure S6. IC_{50} determination using the FRET-based Nsp15 activity assay. Plots showing the change in fluorescence (excitation 495 nm, emission 520 nm) due to the cleavage of 5'-6-FAM-dArUdAdA-6-TAMRA-3' substrate by wild-type SARS-CoV-2 Nsp15. Reactions were allowed to proceed for 30 min at 25°C in the presence of 150 nM of SARS-CoV-2 Nsp15, 1.2 μM substrate, and increasing concentrations of various rhodanine analogs. IC_{50} values (Mean ± SD) were calculated by fitting dose-response curves to log[inhibitor] vs. response--Variable slope curves (four parameters) in GraphPad Prism Y=Bottom + (Top-Bottom)/(1+10^((LogIC50-X)*HillSlope)). Error bars represent the standard deviation of duplicate samples.

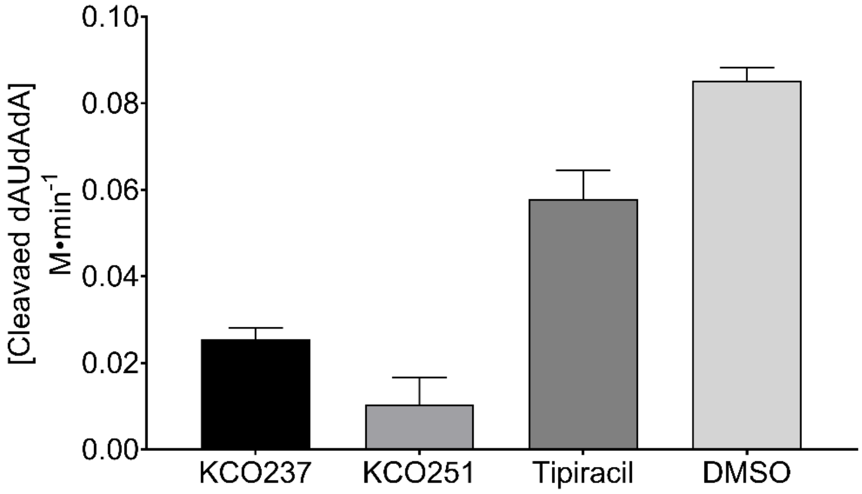

Figure S7. Inhibition of wild-type SARS-CoV-2 Nsp15 activity in HEPES buffer assay conditions. Bar graph showing the change in fluorescence (excitation 495 nm, emission 520 nm) due to the cleavage of 5'-6-FAM-dArUdAdA-6-TAMRA-3' substrate by wild-type SARS-CoV-2 Nsp15. Reactions were allowed to proceed for 30 min at 25°C in presence of 10 nM of SARS-CoV-2 Nsp15, 500 nM substrate in assay buffer (20 mM HEPES-KOH, pH 7.5, 50 mM KCl, 1 mM DTT, 5 mM $MnCl_2$), and 100 μM of tipiracil, KCO237 and KCO251. Error bars represent the standard deviation of duplicate samples.

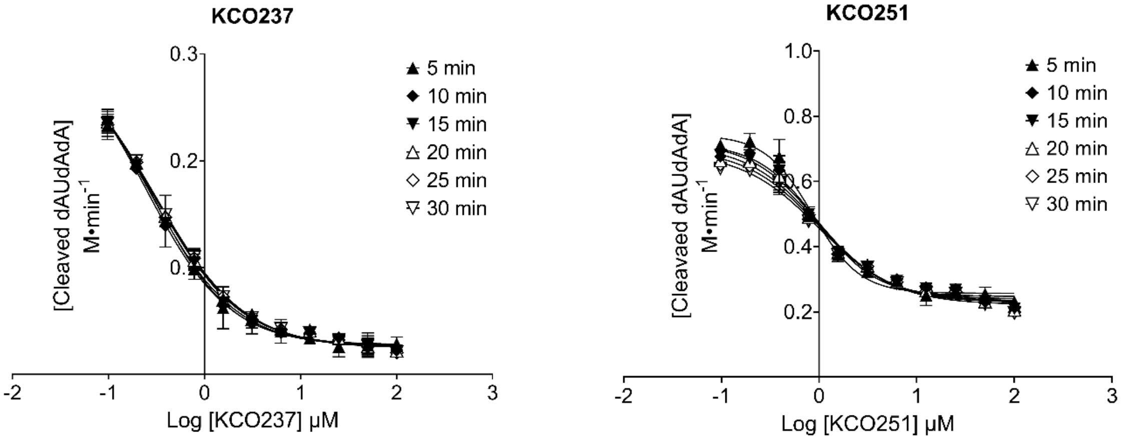

Figure S8. Effect of time on IC_{50}. Plots showing the change in fluorescence (excitation 495 nm, emission 520 nm) due to the cleavage of 5′-6-FAM-dArUdAdA-6-TAMRA-3′ substrate by wild-type SARS-CoV-2 Nsp15. Reactions were allowed to proceed for 30 min at 25°C in the presence of 150 nM of SARS-CoV-2 Nsp15, 1.2 μM substrate, and increasing concentrations of various rhodanine analogs. IC_{50} values were calculated by fitting dose-response curves to log[inhibitor] vs. response--Variable slope curves (four parameters) in GraphPad Prism Y=Bottom + (Top-Bottom)/(1+10^((LogIC50-X)*HillSlope)). Error bars represent the standard deviation of duplicate samples.

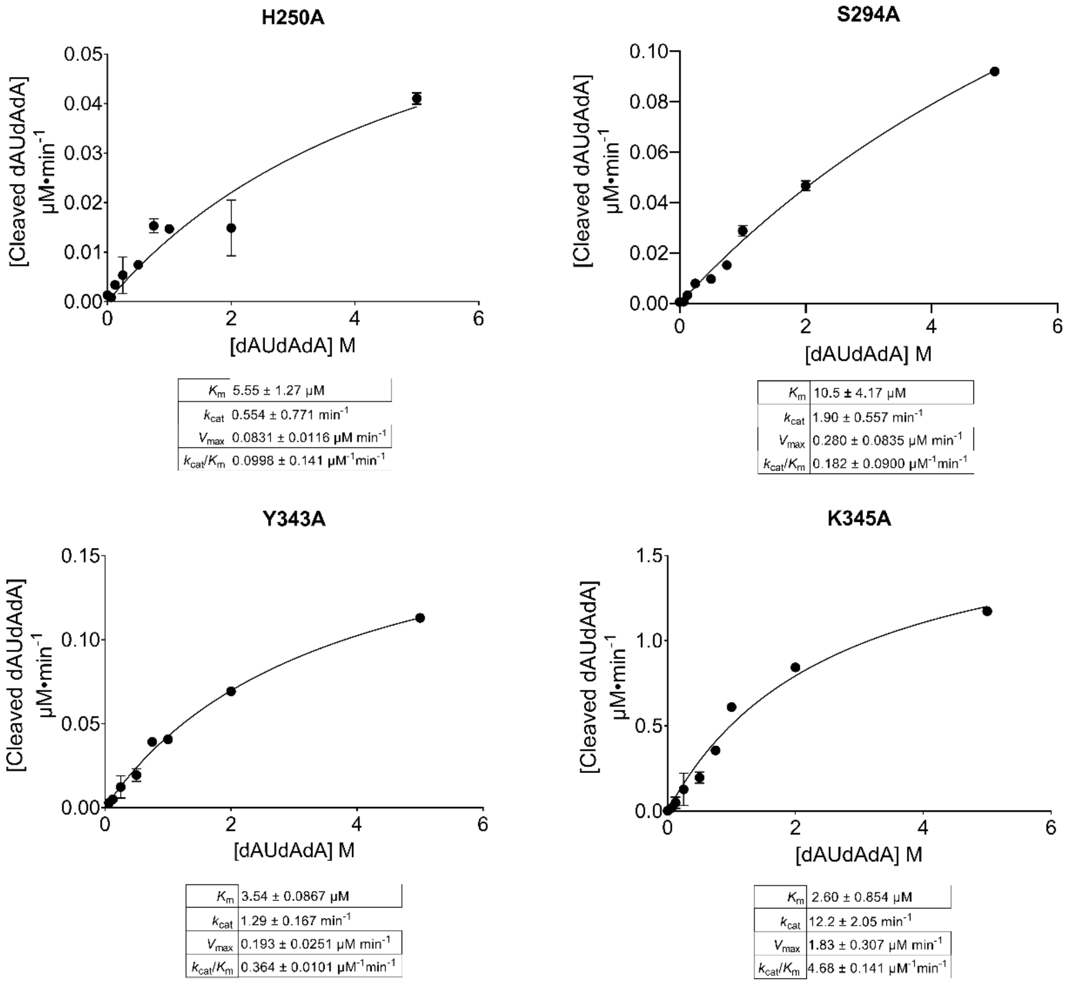

Figure S9. Kinetic parameters of mutant SARS-CoV-2 Nsp15 variants. Plots showing the relationship between mutants SARS-CoV-2 Nsp15 activity rates and the concentration of the substrate 5’-6-FAM-dArUdAdA-6-TAMRA-3’. Curves were fitted to the Michaelis-Menten equation using GraphPad Prism (v = Vmax[S]/(KM + [S])). Error bars represent the standard deviation of duplicate samples.

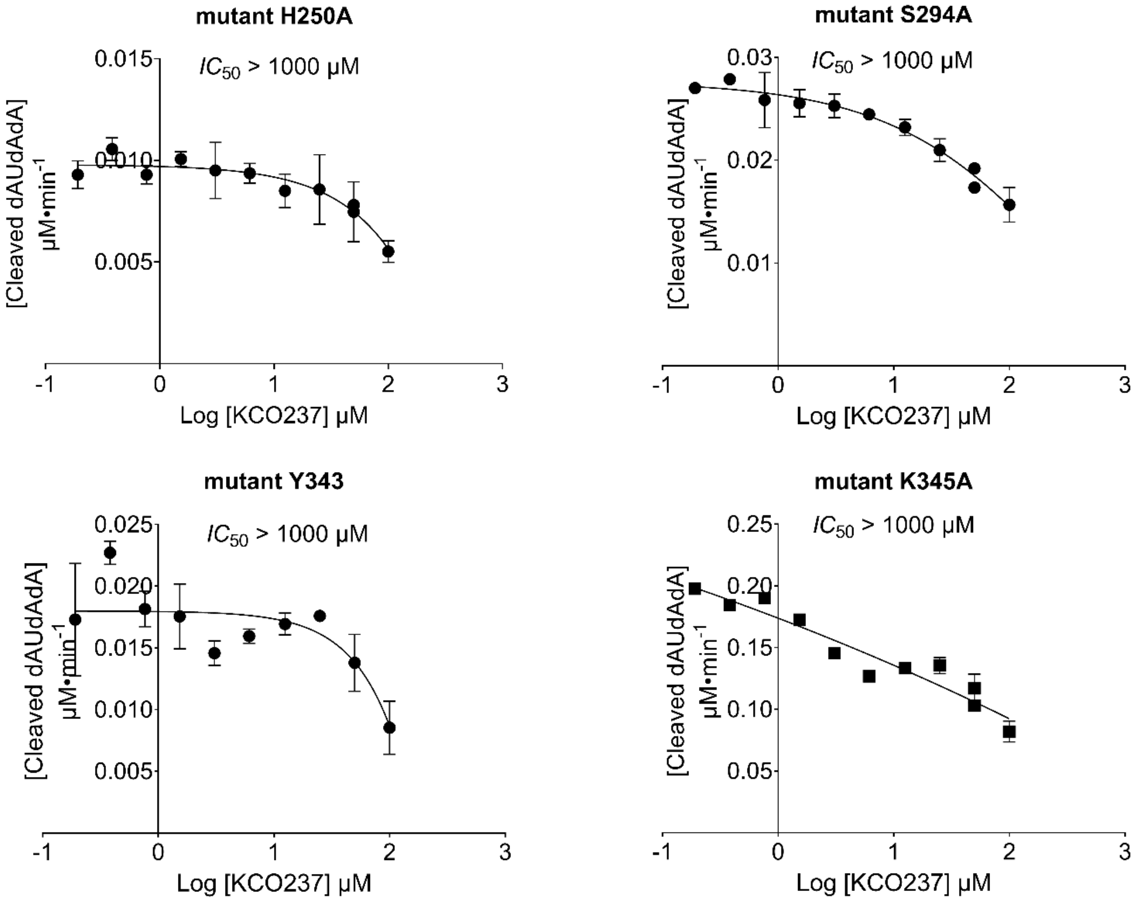

Figure S10. Mutant Nsp15 variants inhibition by KCO237. Plots showing the change in fluorescence (excitation 495 nm, emission 520 nm) due to the cleavage of 5'-6-FAM-dArUdAdA-6-TAMRA-3' substrate by wild-type SARS-CoV-2 Nsp15. Reactions were allowed to proceed for 30 min at 25°C in the presence of 150 nM of SARS-CoV-2 Nsp15, 1.2 µM substrate, and increasing concentrations of KCO237. Points were fitted to a dose-response curves to log[inhibitor] vs. response--Variable slope curves (four parameters) in GraphPad Prism Y=Bottom + (Top-Bottom)/(1+10^((LogIC50-X)*HillSlope)). Error bars represent the standard deviation of duplicate samples.

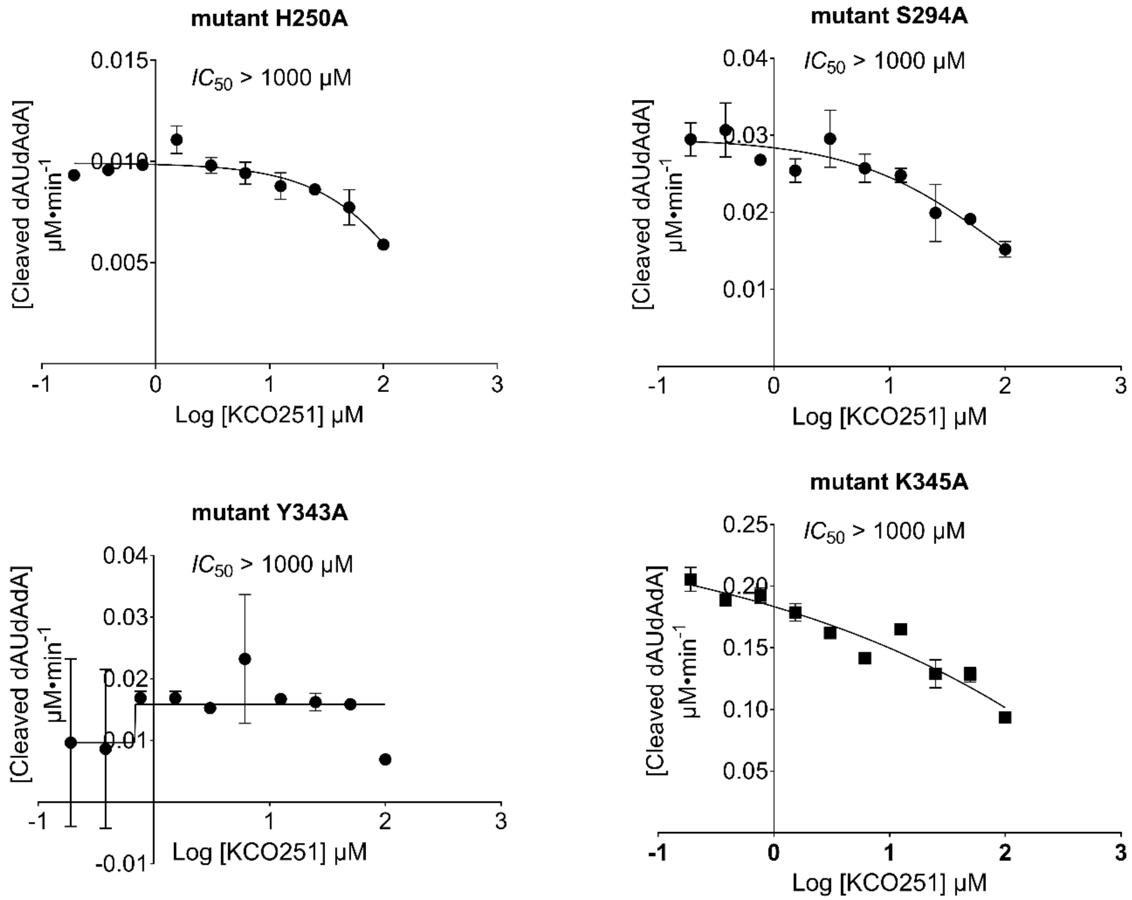

Figure S11. Mutant Nsp15 variants inhibition by KCO251. Plots showing the change in fluorescence (excitation 495 nm, emission 520 nm) due to the cleavage of 5'-6-FAM-dArUdAdA-6-TAMRA-3' substrate by wild-type SARS-CoV-2 Nsp15. Reactions were allowed to proceed for 30 min at 25°C in the presence of 150 nM of SARS-CoV-2 Nsp15, 1.2 μM substrate, and increasing concentrations of KCO251. Points were fitted to a dose-response curves to log[inhibitor] vs. response--Variable slope curves (four parameters) in GraphPad Prism Y=Bottom + (Top-Bottom)/(1+10^((LogIC50-X)*HillSlope)). Error bars represent the standard deviation of duplicate samples.

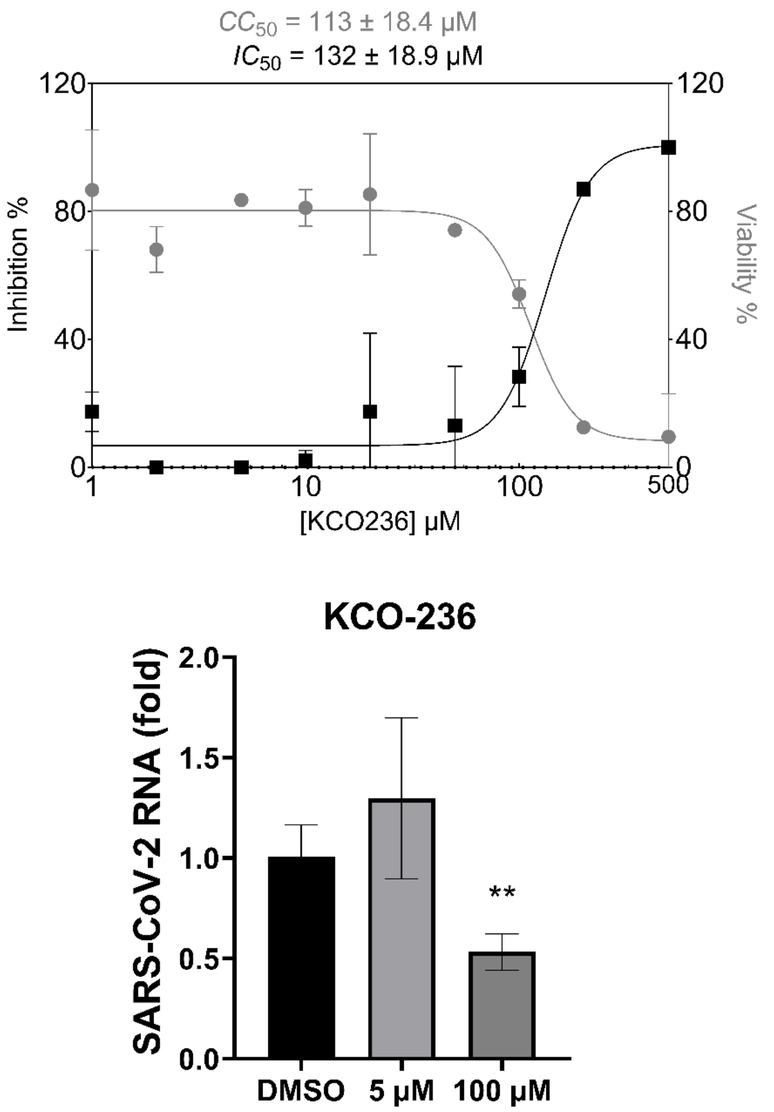

Figure S12. Activity of analog KCO236 in cells. *A,* plots showing the effect of increasing concentrations of Nsp15 inhibitors on viral inhibition (grey curve), and cell viability (black curve) relative to DMSO control sample. To determine viral inhibition, viral titers were measured by plaque assay following a 24 h infection of Vero E6 cells with SARS-CoV-2 (MOI = 0.1) in presence of increasing concentrations the of KCO236. Cell viability was determined by measuring the intracellular concentrations of ATP in presence similar increasing concentrations of KCO236. IC50 and CC50 values (Mean ± SD) were calculated by fitting dose-response curves to [inhibitor] vs. response--Variable slope curves (four parameters) in GraphPad Prism Y=Bottom + (Top-Bottom)/(1+(IC50/X)^HillSlope) in GraphPad Prism. *B,* bar graphs showing the fold of viral gene expression in VERO E6 cells in the absence and presence of different doses of Nsp15 inhibitor. SARS-CoV-2 RNA and human GAPDH mRNA (housekeeping gene) were measured by qRT-PCR following a 24 h infection of Vero E6 cells with SARS-CoV-2 (MOI = 0.1). Viral gene expression fold was calculated using the 2(−ΔΔCT) method. Error bars represent the standard deviation of duplicate samples. ** denotes $p < 0.01$, and *** denotes $p < 0.001$ significance using a one-tailed Student's t test versus the DMSO control.

Table S1

Statistical test for inhibition models of analog KCO237[a]

Null hypothesis	Noncompetitive	Uncompetitive	Competitive
Alternative hypothesis	Mixed	Mixed	Mixed
P value	0.0049	0.0104	<0.0001
Conclusion (alpha = 0.05)	Reject null hypothesis	Reject null hypothesis	Reject null hypothesis
Preferred model	Mixed	Mixed	Mixed
F (DFn, DFd)	8.331 (1, 86)	6.860 (1, 86)	228.6 (1, 86)

[a] Extra sum-of-squares F-test comparison method

Table S2

Statistical test for inhibition models of analog KCO251[a]

Null hypothesis	Noncompetitive	Uncompetitive	Competitive
Alternative hypothesis	Mixed	Mixed	Mixed
P value	0.0077	0.0327	<0.0001
Conclusion (alpha = 0.05)	Reject null hypothesis	Reject null hypothesis	Reject null hypothesis
Preferred model	Mixed	Mixed	Mixed
F (DFn, DFd)	7.44 (1, 86)	4.714 (1, 86)	94.79 (1, 86)

[a] Extra sum-of-squares F-test comparison method

Table S3

Predicted K_i values for a noncompetitive inhibition model[a]

**Compound**	**Predicted K_i (μM)**
KCO035	7.14
KCO036	9.33
KCO048	1.27
KCO230	4.50
KCO231	4.34
KCO234	7.30
KCO235	4.10
KCO236	0.857
KCO237	0.229
KCO238	ND
KCO249	1.28
KCO250	ND
KCO251	0.856

[a] IC50-toKi converter used for calculations (1)

Table S4
Calculated Lipinski's rule of five and Veber's rule for rhodanine analogs[a]

Compound	Lipinski's Rule of Five						Veber's Rule		
	MW	Log P (<5)	No. of HBA (<10)	No. of HBD (<5)	MR (40-130)	Violations	TPSA (<140 Å²)	No. of RB (<10)	Violations
KCO35	331.39	4.08	2	1	103.28	0	71.47	2	0
KCO36	231.27	4.08	2	1	68.27	0	71.47	2	0
KCO48	282.32	2.54	3	1	83.57	0	84.36	2	0
KCO230	347.45	4.63	1	1	110.48	0	86.49	2	0
KCO231	283.34	2.74	2	1	84.94	0	71.47	2	0
KCO234	282.32	2.44	3	1	83.57	0	84.36	2	0
KCO235	282.32	2.45	3	1	83.57	0	84.36	2	0
KCO236	282.32	2.45	3	1	83.57	0	84.36	2	0
KCO237	298.38	3.07	2	1	90.77	0	99.38	2	0
KCO238	345.41	3.07	2	0	108.18	0	62.68	2	0
KCO249	297.39	3.71	1	1	92.98	0	86.49	2	0
KCO250	295.36	3.71	2	0	90.68	0	62.68	2	0
KCO251	298.38	2.97	2	1	90.77	0	99.38	2	0

HBD: hydrogen bond donor
HAD: hydrogen bond acceptor
MR: Molecular refractivity
TPSA: total polar surface area
RB: rotatable bons
[a] SwissADME used for calculations (2)

References:

1. Cer RZ, Mudunuri U, Stephens R, Lebeda FJ. IC50-to-Ki: a web-based tool for converting IC50 to Ki values for inhibitors of enzyme activity and ligand binding. Nucleic Acids Res. 2009;37(Web Server issue):W441-5.
2. Daina A, Michielin O, Zoete V. SwissADME: a free web tool to evaluate pharmacokinetics, drug-likeness and medicinal chemistry friendliness of small molecules. Sci Rep. 2017;7:42717.
